# Individual Variation in Pheromone Response Correlates with Reproductive Traits and Brain Gene Expression in Worker Honey Bees

**DOI:** 10.1371/journal.pone.0009116

**Published:** 2010-02-09

**Authors:** Sarah D. Kocher, Julien F. Ayroles, Eric A. Stone, Christina M. Grozinger

**Affiliations:** 1 Department of Genetics, North Carolina State University, Raleigh, North Carolina, United States of America; 2 Department of Statistics, North Carolina State University, Raleigh, North Carolina, United States of America; 3 Department of Entomology, North Carolina State University, Raleigh, North Carolina, United States of America; 4 W.M. Keck Center for Behavioral Biology, North Carolina State University, Raleigh, North Carolina, United States of America; Centre de Recherches su la Cognition Animale - Centre National de la Recherche Scientifique and Université Paul Sabatier, France

## Abstract

**Background:**

Variation in individual behavior within social groups can affect the fitness of the group as well as the individual, and can be caused by a combination of genetic and environmental factors. However, the molecular factors associated with individual variation in social behavior remain relatively unexplored. We used honey bees (*Apis mellifera*) as a model to examine differences in socially-regulated behavior among individual workers, and used transcriptional profiling to determine if specific gene expression patterns are associated with these individual differences. In honey bees, the reproductive queen produces a pheromonal signal that regulates many aspects of worker behavior and physiology and maintains colony organization.

**Methodology/Principal Findings:**

Here, we demonstrate that there is substantial natural variation in individual worker attraction to queen pheromone (QMP). Furthermore, worker attraction is negatively correlated with ovariole number—a trait associated with reproductive potential in workers. We identified transcriptional differences in the adult brain associated with individual worker attraction to QMP, and identified hundreds of transcripts that are organized into statistically-correlated gene networks and associated with this response.

**Conclusions/Significance:**

Our studies demonstrate that there is substantial variation in worker attraction to QMP among individuals, and that this variation is linked with specific differences in physiology and brain gene expression patterns. This variation in individual response thresholds may reveal underlying variation in queen-worker reproductive conflict, and may mediate colony function and productivity by creating variation in individual task performance.

## Introduction

Behavior is a complex trait, and dramatic individual differences can arise from complex interactions between genotype and environment. The majority of the studies of the genes and molecular pathways associated with behavioral variation have focused on distinct groups of individuals from different genetic backgrounds, individuals in physiologically-distinct behavioral states, or individuals in substantially different environmental contexts [1]. However, there can be significant individual variation among relatively similar individuals within a population. Studies in vertebrates have demonstrated that there is a substantial amount of individual variation in reaction norms [2] and brain gene expression patterns [3]–[5] associated with physiological or life history differences that are adaptive in different environmental contexts. Individual differences in reproductive behaviors in male cichlid and female swordtail fish have been associated with differences in brain expression patterns [6], [7]. Individual variation in behavior can increase the productivity and success of a group as well as playing a role in maximizing individual fitness. However, individual differences in behavior in social groups have not been broadly examined [2], [8]. Here, we examine the molecular and physiological factors associated with individual variation in response to social stimuli in honey bees, one of the best studied models for social behavior.

Individual honey bees vary dramatically in their behavior due to genetic, developmental, and physiological differences. Environmental and developmental factors determine whether female bees develop as facultatively sterile workers or highly-fecund queens; these caste differences are produced by differential nutrition during larval stages, which triggers dramatically different developmental trajectories in the larvae. These nutritional cues are thought to lead to differences in gene methylation and gene expression [9]–[11]. In addition to this reproductive division of labor, there is also an age-related worker division of labor, where individuals transition through a variety of tasks throughout their lifetime, and workers in these behavioral states have significant differences in physiological traits and brain gene expression [12]–[19]. Developmental and environmental factors affect worker task specialization in adulthood. For example, variation in worker ovariole number is correlated with variation in multiple behavioral and physiological traits in the same individuals, including foraging-preference and vitellogenin levels [20], and differences in larval diet can greatly influence the ovariole number [21], [22]. Furthermore, worker-destined larvae reared with high-nutrition diets are more likely to develop their ovaries in the absence of a queen than workers reared in low-nutrition diets [23]. Genotypic differences resulting in behavioral variation have been widely documented as well; one of the most dramatic examples of this is variation in defensive behavior between Africanized and European strains of honey bees [24], [25]. Substantial intracolonial variation in worker physiology and behavior is thought to play an important role in increasing colony function and productivity through task specialization [8], [26], [27]. Indeed, it has been demonstrated that increased genetic variation is important in increasing colony health and fitness [28]–[31]. However, the molecular and physiological factors associated with *individual* variation in performance of the same social task within a colony have not been well-characterized.

In addition to other physical cues, such as food storage levels, chemical communication plays a critical role in colony organization and the regulation of worker social behavior. While there have been many pheromones identified in honey bees, including brood pheromone [32], worker pheromone [33], alarm pheromone [34] and Nasonov pheromone [35], the pheromone produced by the queen is arguably one of the best studied and most important, and regulates many aspects of worker physiology and behavior. Queen pheromone is a complex blend of multiple chemicals, but a five-component subset of these, known as queen mandibular pheromone (QMP), produces many of the effects of a live queen on worker physiology and behavior (reviewed in [36], [37]). Exposure to QMP inhibits worker ovarian development, thereby maintaining the reproductive dominance of the queen. QMP also controls age-related division of labor by reducing the rate at which bees transition from brood care to foraging behavior, inhibits queen replacement, and causes global changes in brain gene expression (reviewed in [36], [37]). QMP also attracts workers to the queen and elicits queen attendance in the form of a *retinue response*, where workers surround, lick, feed, and antennate her, and subsequently spread the pheromone throughout the colony [38]. Pheromones are often considered to be fixed chemical blends that produce stereotyped responses in the receiving individual [39]. However, for pheromones regulating social behaviors in groups of individuals, modulation in responses may be adaptive. Indeed, significant variation in the retinue response to QMP has been found within bee populations [40], and this response is highly heritable [41].

We examined natural variation in individual worker retinue responses within and among colonies of *A.m. carnica* and *A.m. ligustica*, and we correlated this behavioral variation with global brain gene expression patterns and physiological traits. We demonstrated that individual retinue response is negatively correlated with ovariole number – a trait strongly linked to reproductive potential [42] as well as differences in worker behavior and physiology [20]. We used whole-genome transcriptional profiling to identify modules of correlated transcripts expressed in adult worker brains associated with retinue response, and found hundreds of transcriptional differences significantly associated with individual behavioral variation.

## Materials and Methods

### Colony-Level Assays

Colonies were maintained at North Carolina State University in Raleigh, NC according to standard commercial procedures. To determine if there was significant variation in retinue response at the colony-level, we screened 9 colonies headed by single-drone-inseminated queens (SDI; ordered from Glenn Apiaries, Fallbrook, CA) from two different racial lineages of bees: 5 Carniolan colonies (*Apis mellifera carnica*; colonies 1,2,3,7, and 8) and 4 Italian colonies (*Apis mellifera ligustica*; colonies 4,5,6, and 9). We screened two different racial lineages to increase the amount of variation we could observe among colonies. To control for seasonal effects and confirm that the observed variation was consistent throughout the field season, colonies were screened once at the beginning of the season (May 2007) and again at the end (August 2007). To produce bees of a known age, frames containing late-stage pupae were removed from each colony and placed in an incubator (33°C, 50% humidity). Bees were collected 24 hr after eclosion and placed into small (10×10×7 cm) Plexiglas cages (5 cages per colony; 25 bees/cage) in a dark incubator (33°C/50% humidity) and provided with ground pollen, 50% sucrose, and water *ad libitum* as in [15]. QMP (Pherotech International, Delta, British Colombia) was diluted in 1% water/isopropanol. 0.1 queen equivalents of fresh QMP was placed on a glass slide and placed in the cage at the same time every day for a period of 8 days. This quantity of QMP produces similar effects to live queens in young caged bees [15], [43]. This assay has been well established in the literature, and is considered to be strongly representative of the behavior in a natural environment [38], [41], [43]. The assays were conducted under red light beginning 5 minutes after QMP introduction. The observer was blind to the source colony of the workers. The number of bees antennating or licking the pheromone was recorded every 5 minutes for 25 minutes; this was repeated daily on bees 4–8 days old. Bees did not contact a solvent control slide placed in the cage. Colony screens were repeated twice to control for seasonal variation during the course of the experiment. To make our data approximate a normal distribution, the mean frequency of individuals participating in the retinue response was calculated across cages within each colony for each day. This allowed us to use a mixed-model ANOVA without violating assumptions of normality. The data were analyzed using a repeated-measures mixed-model ANOVA in SAS (Cary, NC) with the following model: Y_ghij_  =  μ + colony_g_ + round_h_ + day_i_ + (colony*round)_gh_ + (colony*day)_gi_ + ∼cage_j_(colony_g_) + ε_ghij_ where g indexes the colony, h indexes the time of screen (round 1 or round 2), i indexes the day, and j indexes the cage nested within colony; (colony*round)_gh_ is the fixed colony by round interaction, (colony*day)_gi_ is the fixed colony by day interaction, ∼cage_j_(colony_g_) is a random effect of cage nested in colony, and ε_ghij_ is normally-distributed error. Because individual cages are measured repeatedly in time, a first-order autoregressive model is used to accommodate correlations among measurements from the same cage. Race did not have a significant effect on colony-level retinue response, so this effect was not included in the model.

### Individual Behavioral Assays

To determine if there was significant variation in retinue response among individuals within a colony, individual assays were conducted for all 9 colonies in the same manner as the colony-level assays with two modifications: cages contained only 10 individuals from a single colony, and each individual was uniquely number tagged on her thorax (Opalithplättchen, Endersbach, Germany). Individuals received a score of ‘1’ if they were contacting the slide and a score of ‘0’ if they were not contacting the slide at the time of each observation (5 observations/day, for 5 days). For each colony, the data were analyzed by taking the mean number of responses for an individual each day (for a total of 5 quantitative measurements/individual), using a mixed-model ANOVA in SAS with the following model: y_ijk_  =  μ + day_i_ + ∼cage_j_ + ∼ind_k_(cage_j_) + ∼cage_j_*day_i_ + ε_ijk_, where k indexes the individual, j indexes the cage where the k^th^ individual was housed, and i indexes the day of observation. From these studies, high and low responsive colonies were selected for the subsequent analyses (see results).

### Quantification of Ovariole Number

Worker abdomens were dissected from the same bees scored for retinue response in 6 colonies. The five highest and lowest responding individuals (n = 10 total) were selected from colonies 4, 5, 6 (*A.m. ligustica*), and 8 (*A.m. carnica*), and more individuals were dissected from the high-responding colony (colony 7, *A.m. carnica*; n = 46) and low-responding colony (colony 1, *A.m. carnica*; n = 41) used in the remaining experiments. Abdomens were dissected under RNAlater (Qiagen, Valencia, CA). The number of ovarioles on the left ovary was counted as in [44]. Data points with a Mahalanobis distance greater than 2.5 were excluded from the analysis (6 individuals, JMP software, Cary, NC). We excluded these datapoints as per standard statistical practice, however, the inclusion of these datapoints does not significantly affect the outcome of the analysis (logistic regression, p = 0.0032); the colony effect is still significant (p = 0.0002), but there is also a significant colony*retinue interaction (p = 0.0077). Because there was a natural ranking in the dependent variable (ovariole number), an ordinal logistic regression was used to examine the relationship between ovariole number and retinue response with the following model: Y_ij_ =  μ + retinue_i_ + colony_j_ + retinue_i_*colony_j_ + ε_ij_, where Y_ij_ is the number of ovarioles for each individual, retinue_i_ is the mean frequency of the retinue response averaged over all 5 days of observation, and colony_j_ represents the source colony of each individual.

### Brain Transcriptional Profiling

To identify genes associated with retinue response, we selected the six highest and six lowest responding individuals from highest- and lowest-responding colonies of the same racial lineage (*A.m. carnica*) with retinue responses that were stable over time and contained significant individual variation. RNA from individual brains of the six highest and six lowest responding workers from both colonies was extracted and individually hybridized to whole genome microarrays (supplied by the Robinson laboratory, University of Illinois, Urbana-Champaign). We used adult brains because we were interested in describing the transcriptional profiles associated with individual retinue response behavior. This produced four different groups for comparison: (1) high colony, high responder; (2) high colony, low responder; (3) low colony, high responder; and (4) low colony, low responder. There were six individuals in each group and two technical replicates for each individual, hybridized to a total of 24 microarrays in a loop design (Table S1). Data are available from ArrayExpress (http://www.ebi.ac.uk/microarray-as/ae/), MIAMEXPRESS #56429.

All features with an intensity less than the median array background intensity (300) were removed from the analysis. Transcripts with less than ten measurements (out of a possible 24) were also removed. Data were analyzed using a mixed-model ANOVA approach [45]–[47] implemented in SAS (Cary, NC). All data were log-transformed, and subsequently normalized using a mixed-model ANOVA with the following model: Y_lmn_  =  μ + dye_l_ + array_m_ + block_n_ + dye_l_*array_m_ + ε_lmn_, where Y_lmn_ is expression, dye_l_ and block_n_ (which estimates the print-tip effect on the oligonucleotide arrays) are fixed effects, and array_m_ and its interactions are random effects. Detection of significance for differential expression on residuals was performed using a mixed-model ANOVA with the model: Y_ijklm_  =  μ + behavior_i_(colony_j_) + colony_j_ + spot_k_ + dye_l_ + array_m_ + dye_l_*array_m_ + ε_ijklm_, where Y_ijklm_ is the residual from the previous model, behavior_i_ is individual behavior, and colony_j_ represents colony-level behavior. Behavior_i_, colony_j_, spot_k_, and dye_l_ are fixed effects, and array_m_ and dye_l_*array_m_ are random effects. P-values were corrected for multiple testing using a false discovery rate (FDR) adjustment. The FDR adjusts the p-value such that a specified proportion of the genes are likely to be false positives [48]. We chose an FDR threshold of <0.01 (proc MULTTEST, SAS), suggesting that only 1% of the significant transcripts should be false positives. These genes represent the genes that are significantly associated with variation in individual retinue response within a colony. Clustering analysis of behavioral groups (Hh, Hl, Lh, and Ll) was performed in R using the heatmap function, and bootstrap values were obtained for each node using the pvclust package [49], n = 10,000). Distance was calculated using the Ward method on a correlation-based dissimilarity matrix.

### Identification of Modules of Correlated Transcripts

Correlations between transcripts were calculated as in [50]. Because MMC requires balanced data, we excluded transcripts with any missing datapoints, leaving 662/960 transcripts associated with retinue response. A clustering method designed to elicit transcriptional modules from gene expression profiles, modulated modularity clustering (MMC; [51]), was used to construct putative transcriptional modules from the remaining transcripts associated with retinue response. MMC produces modules of correlated transcripts which can be interpreted as gene networks that are often biologically-meaningful [50], [51].

### Analysis of Significant Gene Lists

GO analysis was conducted on the significantly regulated transcripts using the DAVID functional gene annotation tool [52]. Significant genes were compared to previous studies that have identified candidate genes associated with various traits (see results). The number of overlapping genes was evaluated using a two-tailed Fisher Exact Test to determine if there were significantly more or less genes represented on both lists than expected by chance.

## Results

### Colony-Level Assays

**Figure 1:**
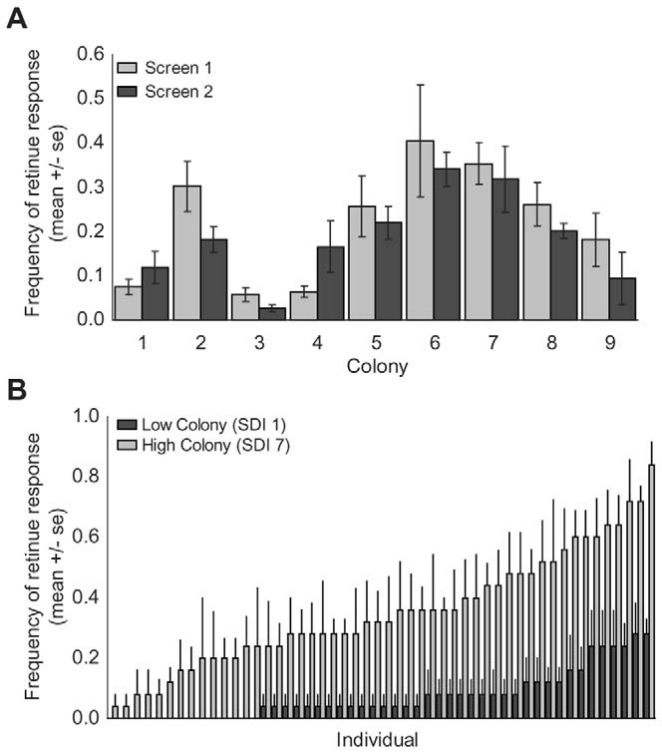
Retinue response varies among colonies and among individuals within a colony. **A**. 9 SDI colonies were screened for retinue response. There were strong differences among colonies in mean retinue response frequency (p<0.0001). There was a significant effect of day on colony response (p = 0.003), but no significant effect of the time of the screen (p = 0.09), cage (p = 0.14), or any significant colony by day (p = 0.10) or colony by screen (p = 0.42) interaction. **B**. Based on the results of the previous screens, two *A.m. carnica* colonies were selected for the subsequent molecular and physiological analyses. The previous retinue bioassay was modified to measure individual variation in response to QMP. There were significant individual differences in retinue response frequency among individuals in both colonies (p<0.001).

To increase the amount of variation that we could observe among colonies, nine single-drone inseminated (SDI) colonies from two different racial lineages of honey bees (*A.m. ligustica* and *A.m. carnica*) were screened in May and August 2007. There were significant differences in retinue response among these colonies (repeated-measures ANOVA, p<0.0001; Figure 1a). The effect of day was significant (p = 0.003). There was no significant effect of round (p = 0.09), nor were the effects of cage nested in colony (p = 0.14), the colony by day interaction (p = 0.10), or the colony by round interaction (p = 0.42) significant (Figure 1a). There was no significant effect of racial lineage on retinue response (ANOVA, p = 0.19, data not shown).

### Individual Assays

Individuals within each of the colonies were marked with a unique number tag and were monitored over several days for retinue response. There were significant differences among individuals within all colonies except one (ANOVA, p<0.005). The exception, colony 3, was composed only of individuals with a very low retinue response score. For the subsequent microarray analyses, we selected individuals from the highest- and lowest-responding colonies of the same racial lineage (*A.m. carnica*) with retinue responses that were stable over time and contained significant individual variation (highest =  colony 7, lowest = colony 1). Mean individual retinue scores for individuals from these two colonies are depicted in Figure 1b.

### Retinue Response Is Negatively Correlated with Ovariole Number

**Figure 2:**
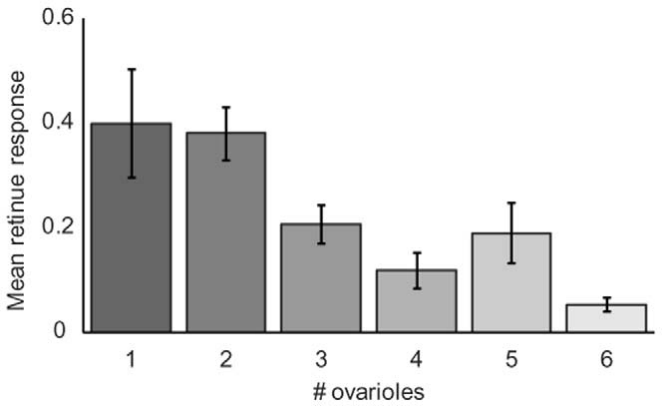
Retinue response is negatively correlated with ovariole number. Ovarioles were dissected and counted from the left, ventral ovary from individual workers from six colonies. A logistic regression demonstrated that there is a strong negative correlation between retinue response and ovariole number (p = 0.0014). There was also a significant colony effect (p<0.0001), but no significant interaction (p = 0.11). A bar graph depicts the average retinue response based on the number of ovarioles comprising the left, ventral ovary.

We examined six colonies for individual differences in ovariole number. There was a significant negative correlation between individual retinue response and ovariole number (Figure 2; logistic regression, p = 0.0014) and a significant colony effect (p<0.0001), but no significant colony*retinue interaction (p = 0.11).

### Transcripts Associated with Individual Variation in Retinue Response

**Figure 3:**
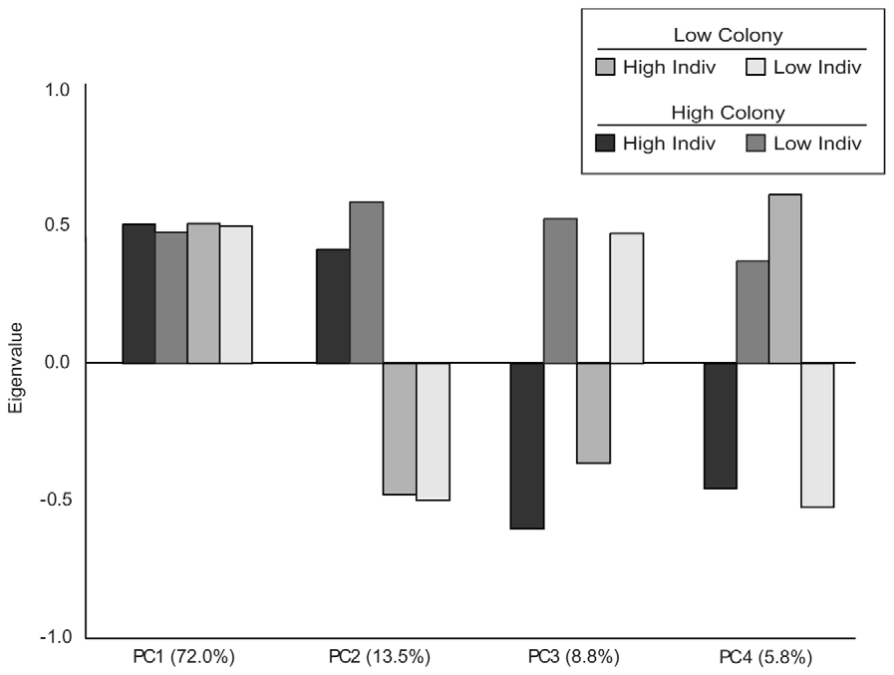
Brain gene expression patterns associated with individual and colony-level differences. Principal component analysis indicates that the primary variance in the dataset is associated with variation in expression among genes, and accounts for 72% of the observed variance (Figure 3, PC1). Furthermore, a large proportion of the variance in transcript abundance is associated with colony-level behavioral differences (PC2, 13.5%) and differences in individual pheromone response (PC3, 8.8%). The final principal component appears to be associated with the interaction between individual and colony-level behaviors, and represents 5.5% of the observed transcriptional variation.

8,000/13,439 transcripts on whole-genome microarrays were expressed in our samples and included in the data analysis. There were 960 transcripts differentially expressed between high and low responders within each of the two colonies with an FDR<0.01 (Table S2). Hierarchical clustering demonstrates the individual behavioral groups cluster based on colony-level differences, with bootstrap values of 100 at both nodes (Figure S1). We used the residuals from our mixed-model normalization ANOVA to conduct a principal component analysis. The results indicate that the primary variance in the dataset (Figure 3, PC1, 72%) is associated with variation in expression levels among genes and shows no clear pattern associated with each behavioral group; this result is unsurprising given that we did not normalize the data among genes. Furthermore, a large proportion of the variance in transcript abundance (PC2, 13.5%) is associated with behavioral differences among colonies, and 8.8% of the variance (PC3) is attributable to differences in individual behavior (Figure 3). Finally, PC4 represents 5.8% of the variance, and appears to be associated with colony-specific behavioral differences in pheromone responsiveness.

Previous studies have demonstrated that colony environment and indirect genetic effects on brain gene expression can be abundant [53], and thus the colony-level effects were accounted for in the statistical model. The 960 significant transcripts associated with retinue response were significantly different among individuals within each colony, and not necessarily associated with individual variation in retinue response among individuals between both colonies. In other words, the magnitude and direction of the transcriptional differences associated with high and low retinue response may have varied between colonies (see methods for detailed description of the analysis). However, across both colonies, the expression levels of all 960 significantly-regulated transcripts were positively correlated (r = 0.23, p<0.0001). 360 transcripts were significantly upregulated in high vs. low responders in both colonies, while 141 were significantly downregulated (Table S4). This represents a significantly greater overlap than expected by chance (one-tailed Fisher Exact test, p = 0.0009), and suggests that these 501 transcripts are consistently regulated regardless of differences in genetic background (though hierarchical clustering still demonstrates that individuals cluster based on colony-level differences; data not shown). The overall gene ontology (GO) biological processes for all 960 significant transcripts are shown in Table S4.

### Identification of Gene Networks Associated with Retinue Response

**Figure 4:**
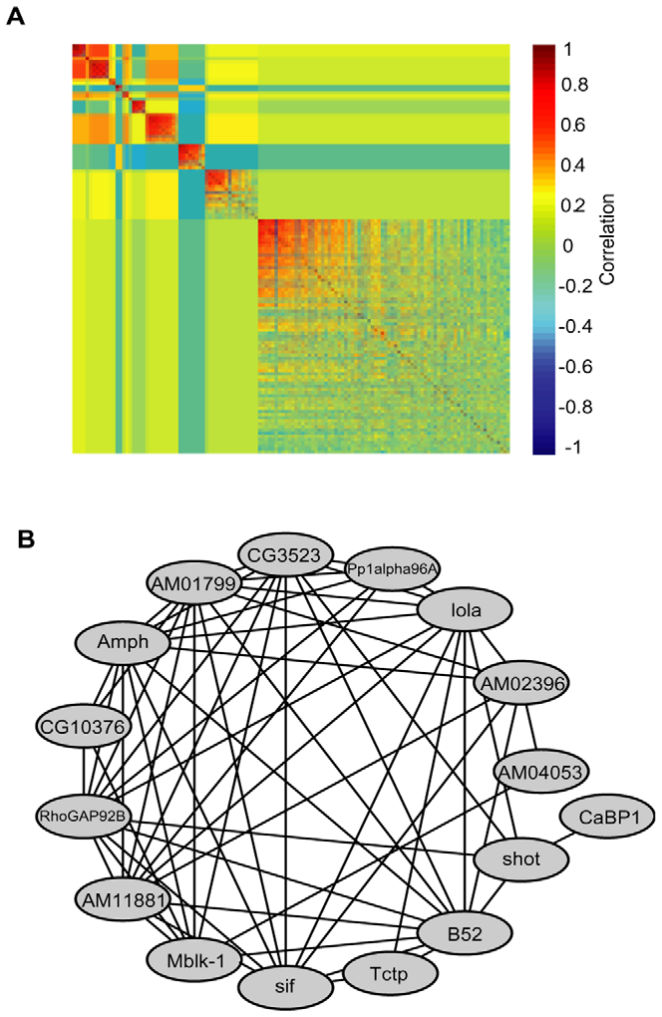
Gene networks associated with retinue response. Gene networks were constructed based on a gene-gene correlation matrix. The colors on the off-diagonal represent the average cross-module absolute correlations. GO analysis identified unique biological or molecular functions for many of these gene networks (Table S3). **A**. From the 960 genes associated with retinue response, 12 genetic modules were identified using MMC with an average |r| = 0.37. **B**. Retinue response module 1 is a statistically-predicted gene network associated with retinue response that contains an overrepresentation of genes involved in axonogenesis. Each node depicts one of the transcripts associated with module 1, and each line represents a statistical correlation between the connected transcripts.

A matrix of pairwise correlations among significant transcripts was constructed in an attempt to elucidate the genetic networks underlying variation in individual retinue response. Modulated modularity clustering (MMC; [51]) was used to identify separable modules of highly correlated transcripts. MMC produces modules of correlated transcripts which can be interpreted as gene networks that are often biologically meaningful [50], [51]. The retinue response genes were partitioned into twelve transcriptional modules with an average |r| = 0.37 (Figure 4a). GO analysis identified unique biological or molecular functions for each gene network; the transcripts, their corresponding modules, and the functional categories for which the modules are enriched are listed in Table S5. Overall, differences in retinue response were correlated with transcripts involved in multiple processes that could alter neural network structure and neural physiology (such as axonogenesis, neuron development, ion channel activity, cell signaling pathways, vesicle mediated transport, and chromatin-mediated regulation of transcription, such as SNF-2 related genes). We selected the first module (associated with axonogenesis) for graphical representation in figure 4b. Each node represents a transcript associated with the axonogenesis gene network, and the lines connecting any two nodes represent a strong statistical correlation between transcripts.

### Comparisons to Previous Studies

Significantly regulated transcripts were compared to previous studies that identified candidate genes associated with various traits in honey bees, including: QMP exposure [15], nursing/foraging behavior [13], methoprene (a juvenile hormone analog) treatment [12], worker ovary activation [14], and pollen hoarding [24]. We compared our list of 960 transcripts associated with retinue response to these previously published studies (Table S6). Significant over- or under-representation was determined using a two-tailed Fisher's Exact Test (Table S6). Several transcripts were represented in more than one gene list; these are listed in Table S7. See the discussion for more detailed coverage of these results.

## Discussion

Variation in how individuals within a social group respond to specific stimuli can lead to both individual and group fitness benefits [26], [54]. Here, we used a cohort of same aged worker bees from a limited genetic background that was reared together under the same environmental conditions, and demonstrated that there is substantial individual variation in response to a pheromone produced by the queen to regulate worker division of labor and individual reproduction. These behavioral differences are also associated with variation in ovariole number, a physiological trait that is set during late larval development. These results suggest that factors that affect larval development – either genetic or environmental – can substantially alter adult worker behavior. Furthermore, the significant differences in brain gene expression demonstrate that high vs low responding bees are indeed in different physiological states, either due to genetic factors, developmental cues that results in fixed physiological differences into adulthood, or environmental differences in QMP exposure. Notably, this phenotypic variation is likely to be even more dramatic in less managed populations of bees. In our studies, variation was reduced because we used offspring of queens mated with a single drone and colonies were maintained in a single apiary, while under natural conditions, queens will mate with many males (10–12 on average; [55]) and are subjected to greater environmental variance.

There are several possible explanations for the maintenance of this variation. For sex pheromones, any deviation from the optimal pheromonal blend or behavioral response may have fitness costs for both males and females [39]; however, for pheromonal signals that regulate other aspects of social behavior, modulation in pheromone responses may be less problematic or even potentially beneficial. Previous work has demonstrated that individual differences in response thresholds to social cues can optimize colony performance [8], [26], [27]. Within social insects, variation in traits such as the initial onset of foraging or sucrose response may increase colony productivity and success [8], [26], [27]. A similar argument could be made for variation in response to queen pheromone. Queen pheromone affects a variety of tasks in the hive, including the transition from nursing to foraging [56] and queen rearing [57]. Variation in sensitivity to queen pheromone could therefore mediate variation in many different social behaviors, and thus, variation in queen pheromone response may play an important role in optimizing colony performance.

Differences in retinue response scores may also translate to alternative behavioral strategies for maximizing individual fitness. Our results suggest that workers with the highest reproductive potential (e.g. the greatest number of ovarioles) avoid the queen, while those with lower reproductive potential are attracted to her. These observations lead to a model in which workers with high reproductive potential are primed to activate their ovaries under queenless conditions, while those with low reproductive potential cooperate with the queen and rear new queens under queenless conditions. In queenless conditions, only a subset of young worker bees will activate their ovaries, and these can come from specific patrilines within the colonies, suggesting that genetic factors regulate the laying worker phenotype [58]–[61]. Interestingly, in *A.m. capensis*, workers that are likely to become reproductively active are indeed more likely to avoid the queen [62]. Furthermore, in the absence of a queen, high-responding bees are more likely to engage in new queen rearing than low-responding individuals [63].

How are retinue response and ovariole number linked at the physiological level? Ovariole number is determined during larval development. It is possible that QMP may regulate ovary development during late larval instar stages, and that bees with higher levels of responsiveness develop fewer ovarioles. This scenario would suggest that larvae can detect QMP. There is no evidence for this in honey bees, though in bumble bees (*Bombus terrestris*), it appears that caste differentiation during larval stages may be regulated by queen pheromone [64]. Alternatively, developmental factors could cause variation in ovariole number, which leads to differences in adult physiology that, in turn, alter retinue response. The dramatic differences in ovariole number observed between queens and workers are regulated by juvenile hormone levels [65] and transcriptional changes in genes associated with metabolic processes [9], [11]. Furthermore, both direct and indirect genetic factors [44], [66] as well as environmental factors, such as nutrition [21]–[23], regulate ovary development in workers. Physiological differences between workers with high vs. low ovariole number could lead to altered processing of the pheromone signals. It is likely this change in processing would occur in the central brain rather than in the peripheral sensory system. Comparisons of the expression and sequence of the 9-ODA responsive pheromone receptor (AmOR11, [67]) revealed no differences in the high and low responding bees from our study (Kocher and Grozinger, unpublished data). Furthermore, previous comparisons of nurses (which are attracted to queen pheromone) and foragers (which are not) revealed no differences in peripheral detection [68]. The observed phenotypic correlation between retinue response and ovariole number suggests that there is a molecular link between these traits. For example, ovariole number may directly influence brain gene expression in such a way to alter behavioral responses to the pheromone. While this could not be directly tested with the current data set, studies are currently underway to test this hypothesis using linkage mapping and physiological manipulations.

We also conducted a set of comparative studies to determine if transcripts associated with individual retinue response behavior were associated with other behavioral or physiological states in honey bees. Of these comparisons, the only significant biases were associated with nursing/foraging behavior and methoprene treatment gene lists. Since forager bees have a lower retinue response compared to young nurse-age bees [68], [69], it is reasonable to assume that the transcriptional profiles of high responders may appear more “nurse-like” than “forager-like.” Indeed, among high responders, fewer genes upregulated in nurses were downregulated in high responders than would be expected by chance; similarly, fewer genes upregulated in foragers were upregulated in high responders. Juvenile hormone levels are higher in foragers than nurses, and treatment with methoprene accelerates the transition to foraging [70]. Furthermore, juvenile hormone levels are decreased by QMP [56]. Consistent with these regulatory pathways, genes upregulated by methoprene were statistically unlikely to be upregulated in the presumably “nurse-like” high responders (p = 0.007). There was no significant overlap among genes associated with QMP exposure [15] or worker ovary activation [14]. However, the lack of overlap is not necessarily surprising given that in contrast to the previous studies, all the bees from the present study were exposed to QMP and none had activated their ovaries (Kocher, personal observation).

### Summary

There is substantial variation in retinue response in adult workers that appears to be associated with physiological traits linked to reproductive potential in honey bees. These traits are likely to be determined by a combination of environmental and genetic factors that shift physiological parameters during development and result in altered behavioral response thresholds in adults. Variation in individual response thresholds may reveal underlying variation in queen-worker reproductive conflict. Natural variation in honey bee pheromone response appears to be widespread [40], and this variation may be potentially adaptive because it could mediate colony function and productivity by creating variation in individual task performance. There appears to be robust modulation in both pheromone production by the queen [47], [71], [72] and worker responses to this pheromone, demonstrating that this behavior is part of an interacting phenotype, and suggesting that the chemical communication process between queens and workers may represent a dialog, rather than a simple, static signal-response system.

## Supporting Information

Table S1 Hybridization scheme. All samples were hybridized using a loop design incorporating dye-swaps.
(0.06 MB DOC)

Table S2 960 significantly-regulated transcripts for retinue response. There were 960 genes that were significantly associated with retinue response at FDR<0.01. \The first column contains the transcript identifier associated with the microarray, and the subsequent columns are the corresponding honey bee predicted gene names (GB names) and fly orthologs (the flybase identifiers) if available.
(0.91 MB DOC)

Table S3 Transcripts consistently regulated across both colonies. 360 transcripts were consistently up- or down-regulated in high responding individuals in both colonies.
(0.63 MB DOC)

Table S4 Biological processes associated with retinue response. Gene ontology analysis of transcripts associated with retinue response.
(0.03 MB DOC)

Table S5 Retinue response modules. Statistical gene networks predicted by MMC for retinue response. Each module was assigned an average degree of correlation among transcripts (avg degree), and each transcript received a degree of correlation between itself and the remaining transcripts from that module (degree). Gene ontologies associated with each module are also included in this table.
(0.64 MB DOC)

Table S6 Comparative genomic analysis. The 960 transcripts associated with retinue response were compared to previously published studies that identified sets of transcripts associated with other behavioral or physiological traits in workers. Because some of the 960 transcripts were up-regulated in high-responders of one colony and down-regulated in high-responders of the other colony, there is some overlap between gene lists. Overall, these patterns suggest that high-responding individuals have brain transcriptional profiles more similar to nurse bees than to forager bees.
(0.04 MB DOC)

Table S7 Comparative studies gene lists. Significantly-regulated transcripts in this study were compared to previously published expression studies in honey bees. The list of overlapping transcripts are included in this file.
(1.40 MB DOC)

Figure S1 Hierarchical clustering. Hierarchical clustering of the 960 significant retinue response genes (FDR<0.01) reveals that behavioral groups cluster based primarily on colony-level differences and not on individual retinue response behavior.
(1.19 MB TIF)
