# Supplementary material for: Individual Variation in Pheromone Response Correlates with Reproductive Traits and Brain Gene Expression in Worker Honey Bees
Source: PLoS One. 2010 Feb 9;5(2):e9116. doi: 10.1371/journal.pone.0009116 (PMC2817734; doi:10.1371/journal.pone.0009116)
Supplement: Table S1 — Hybridization scheme. All samples were hybridized using a loop design incorporating dye-swaps. (0.06 MB DOC) [file pone.0009116.s001.doc]

| Array | Group | Dye |
| --- | --- | --- |
| **1** | HH1 | Cy3 |
| **1** | HL1 | Cy5 |
| **2** | HL1 | Cy3 |
| **2** | LL1 | Cy5 |
| **3** | LL1 | Cy3 |
| **3** | LH1 | Cy5 |
| **4** | LH1 | Cy3 |
| **4** | HH2 | Cy5 |
| **5** | HH2 | Cy3 |
| **5** | HL2 | Cy5 |
| **6** | HL2 | Cy3 |
| **6** | LL2 | Cy5 |
| **7** | LL2 | Cy3 |
| **7** | LH2 | Cy5 |
| **8** | LH2 | Cy3 |
| **8** | HH3 | Cy5 |
| **9** | HH3 | Cy3 |
| **9** | HL3 | Cy5 |
| **10** | HL3 | Cy3 |
| **10** | LL3 | Cy5 |
| **11** | LL3 | Cy3 |
| **11** | LH3 | Cy5 |
| **12** | LH3 | Cy3 |
| **12** | HH4 | Cy5 |
| **13** | HH4 | Cy3 |
| **13** | HL4 | Cy5 |
| **14** | HL4 | Cy3 |
| **14** | LL4 | Cy5 |
| **15** | LL4 | Cy3 |
| **15** | LH4 | Cy5 |
| **16** | LH4 | Cy3 |
| **16** | HH5 | Cy5 |
| **17** | HH5 | Cy3 |
| **17** | HL5 | Cy5 |
| **18** | HL5 | Cy3 |
| **18** | LL5 | Cy5 |
| **19** | LL5 | Cy3 |
| **19** | LH5 | Cy5 |
| **20** | LH5 | Cy3 |
| **20** | HH6 | Cy5 |
| **21** | HH6 | Cy3 |
| **21** | HL6 | Cy5 |
| **22** | HL6 | Cy3 |
| **22** | LL6 | Cy5 |
| **23** | LL6 | Cy3 |
| **23** | LH6 | Cy5 |
| **24** | LH6 | Cy3 |
| **24** | HH1 | Cy5 |

**S1. Hybridization Scheme.** All samples were hybridized using a loop design incorporating dye-swaps.
