# Supplementary material for: Individual Variation in Pheromone Response Correlates with Reproductive Traits and Brain Gene Expression in Worker Honey Bees
Source: PLoS One. 2010 Feb 9;5(2):e9116. doi: 10.1371/journal.pone.0009116 (PMC2817734; doi:10.1371/journal.pone.0009116)
Supplement: Table S2 — 960 significantly-regulated transcripts for retinue response. There were 960 genes that were significantly associated with retinue response at FDR<0.01. \The first column contains the transcript identifier associated with the microarray, and the subsequent columns are the corresponding honey bee predicted gene names (GB names) and fly orthologs (the flybase identifiers) if available. (0.91 MB DOC) [file pone.0009116.s002.doc]

| **AM** | **GB** | **XM** | **FlyBaseID** | **Gene Name** |
| --- | --- | --- | --- | --- |
| AM00021 |  |  |  |  |
| AM00021R |  |  |  |  |
| AM00045 |  |  |  |  |
| AM00070 |  |  |  |  |
| AM00073 |  |  |  |  |
| AM00109 |  |  |  |  |
| AM00148 |  |  |  |  |
| AM00159 |  |  |  |  |
| AM00244 | GB15899 |  | FBgn0041621 | Or82a |
| AM00333 | GB16692 | XM_396566.3 | FBgn0039805 | CG12045 |
| AM00351 | GB13473 | NM_001011642.1 | FBgn0035544 | CG15021 |
| AM00352 | GB13473 | NM_001011613.1 | FBgn0035544 | CG15021 |
| AM00353 | GB13473 | XM_001123211.1 | FBgn0035544 | CG15021 |
| AM00354 | GB17782 | NM_001011642.1 | FBgn0035544 | CG15021 |
| AM00355 | GB17782 | NM_001011642.1 | FBgn0035544 | CG15021 |
| AM00356 | GB17782 | XM_001123211.1 | FBgn0035544 | CG15021 |
| AM00357 | GB17782 | NM_001011613.1 | FBgn0035544 | CG15021 |
| AM00358 | GB13473-RB | NM_001011613.1 | FBgn0035544 | CG15021 |
| AM00359 |  |  |  |  |
| AM00360 | GB17782 | NM_001011642.1 | FBgn0035544 | CG15021 |
| AM00366 |  |  |  |  |
| AM00378 |  |  |  |  |
| AM00391R |  |  |  |  |
| AM00408 |  |  |  |  |
| AM00413R |  |  |  |  |
| AM00417R |  |  |  |  |
| AM00429 |  |  |  |  |
| AM00429R |  |  |  |  |
| AM00439R |  |  |  |  |
| AM00446 |  |  |  |  |
| AM00459R |  |  |  |  |
| AM00463R |  |  |  |  |
| AM00464 |  |  |  |  |
| AM00464R |  |  |  |  |
| AM00496 |  |  |  |  |
| AM00523 |  |  |  |  |
| AM00529 |  |  |  |  |
| AM00539R |  |  |  |  |
| AM00558 |  |  |  |  |
| AM00597 |  |  |  |  |
| AM00598 |  |  |  |  |
| AM00612 |  |  |  |  |
| AM00626R |  |  |  |  |
| AM00647R |  |  |  |  |
| AM00683 |  |  |  |  |
| AM00688 |  |  |  |  |
| AM00705 |  |  |  |  |
| AM00717R |  |  |  |  |
| AM00733 |  |  |  |  |
| AM00737 |  |  |  |  |
| AM00749 |  |  |  |  |
| AM00757R | GB12929 | XM_392548.3 | FBgn0003036 | para |
| AM00761R |  |  |  |  |
| AM00763 |  |  |  |  |
| AM00787 |  |  |  |  |
| AM00823 |  |  |  |  |
| AM00826 |  |  |  |  |
| AM00830 |  |  |  |  |
| AM00859 |  |  |  |  |
| AM00894 |  |  |  |  |
| AM00904 |  |  |  |  |
| AM00951 |  |  |  |  |
| AM00954 |  |  |  |  |
| AM00969 |  |  |  |  |
| AM01004 |  |  |  |  |
| AM01005 |  |  |  |  |
| AM01033 |  |  |  |  |
| AM01037 |  |  |  |  |
| AM01043 |  |  |  |  |
| AM01048 |  |  |  |  |
| AM01053 |  |  |  |  |
| AM01093 |  |  |  |  |
| AM01108 |  |  |  |  |
| AM01112 |  |  |  |  |
| AM01138 | GB15745 |  | FBgn0036975 | CG5618 |
| AM01159 |  |  |  |  |
| AM01171 |  |  |  |  |
| AM01177 |  |  |  |  |
| AM01178 |  |  |  |  |
| AM01193 |  |  |  |  |
| AM01196 |  |  |  |  |
| AM01226 |  |  |  |  |
| AM01228 |  |  |  |  |
| AM01232 |  |  |  |  |
| AM01246 |  |  |  |  |
| AM01249 |  |  |  |  |
| AM01252 |  |  |  |  |
| AM01255 |  |  |  |  |
| AM01296 |  |  |  |  |
| AM01304 |  |  |  |  |
| AM01313 |  |  |  |  |
| AM01324 |  |  |  |  |
| AM01334 |  |  |  |  |
| AM01335 |  |  |  |  |
| AM01340 |  |  |  |  |
| AM01365 |  |  |  |  |
| AM01368 |  |  |  |  |
| AM01391 |  |  |  |  |
| AM01394 |  |  |  |  |
| AM01403 |  |  |  |  |
| AM01416 |  |  |  |  |
| AM01423 |  |  |  |  |
| AM01427 |  |  |  |  |
| AM01447 |  |  |  |  |
| AM01474 |  |  |  |  |
| AM01490 | GB10237 | XR_014928.1 | FBgn0019652 | sif |
| AM01493 |  |  |  |  |
| AM01508 |  |  |  |  |
| AM01515 |  |  |  |  |
| AM01519 |  |  |  |  |
| AM01524 |  |  |  |  |
| AM01529 |  |  |  |  |
| AM01542 |  |  |  |  |
| AM01552 |  |  |  |  |
| AM01558 |  |  |  |  |
| AM01596 |  |  |  |  |
| AM01607 |  |  |  |  |
| AM01619 |  |  |  |  |
| AM01631 |  |  |  |  |
| AM01634 |  |  |  |  |
| AM01636 |  |  |  |  |
| AM01646 |  |  |  |  |
| AM01667 |  |  |  |  |
| AM01669 |  |  |  |  |
| AM01686 |  |  |  |  |
| AM01713 |  |  |  |  |
| AM01722 |  |  |  |  |
| AM01735 |  |  |  |  |
| AM01737 |  |  |  |  |
| AM01760 |  |  |  |  |
| AM01763 |  |  |  |  |
| AM01766 |  |  |  |  |
| AM01776 |  |  |  |  |
| AM01798 |  |  |  |  |
| AM01799 |  |  |  |  |
| AM01804 |  | NM_001011629.1 | FBgn0013948 | Eip93F |
| AM01818 |  |  |  |  |
| AM01833 |  |  |  |  |
| AM01838 |  |  |  |  |
| AM01845 |  |  |  |  |
| AM01867 |  |  |  |  |
| AM01877 |  |  |  |  |
| AM01880 |  |  |  |  |
| AM01881 |  |  |  |  |
| AM01889 |  |  |  |  |
| AM01892 |  |  |  |  |
| AM01901 |  |  |  |  |
| AM01911 |  |  |  |  |
| AM01920 |  | XM_001119924.1 | FBgn0001297 | kay |
| AM01921 |  |  |  |  |
| AM01939 |  |  |  |  |
| AM01973 |  |  |  |  |
| AM01974 |  |  |  |  |
| AM01977 |  |  |  |  |
| AM01979 |  |  |  |  |
| AM01986 |  |  |  |  |
| AM01994 |  |  |  |  |
| AM01996 |  |  |  |  |
| AM02006 |  |  |  |  |
| AM02013 |  | XM_001121750.1 | FBgn0031879 | SP1070 |
| AM02021 | GB15792 | XM_391860.3 | FBgn0004587 | B52 |
| AM02061 |  |  |  |  |
| AM02062 |  |  |  |  |
| AM02070 |  |  |  |  |
| AM02110 |  |  |  |  |
| AM02115 |  |  |  |  |
| AM02118 |  |  |  |  |
| AM02120 |  |  |  |  |
| AM02125 |  |  |  |  |
| AM02128 |  |  |  |  |
| AM02132 |  | NM_001011629.1 | FBgn0013948 | Eip93F |
| AM02135 |  |  |  |  |
| AM02144 |  |  |  |  |
| AM02150 |  |  |  |  |
| AM02154 |  |  |  |  |
| AM02158 |  |  |  |  |
| AM02160 |  |  |  |  |
| AM02161 |  |  |  |  |
| AM02162 |  |  |  |  |
| AM02189 |  |  |  |  |
| AM02196 |  |  |  |  |
| AM02201 |  |  |  |  |
| AM02224 |  |  |  |  |
| AM02239 |  |  |  |  |
| AM02247 |  |  |  |  |
| AM02259 |  |  |  |  |
| AM02265 |  |  |  |  |
| AM02281 |  |  |  |  |
| AM02315 |  |  |  |  |
| AM02318 |  |  |  |  |
| AM02324 |  |  |  |  |
| AM02333 |  |  |  |  |
| AM02366 |  |  |  |  |
| AM02367 |  |  |  |  |
| AM02377 |  |  |  |  |
| AM02387 |  |  |  |  |
| AM02396 |  |  |  |  |
| AM02398 |  |  |  |  |
| AM02412 |  |  |  |  |
| AM02418 |  |  |  |  |
| AM02419 |  |  |  |  |
| AM02439 |  |  |  |  |
| AM02442 |  |  |  |  |
| AM02481 |  |  |  |  |
| AM02488 |  |  |  |  |
| AM02496 |  |  |  |  |
| AM02522 |  |  |  |  |
| AM02530 |  |  |  |  |
| AM02537 |  |  |  |  |
| AM02540 |  |  |  |  |
| AM02542 |  |  |  |  |
| AM02560 |  |  |  |  |
| AM02567 |  | NM_001011629.1 | FBgn0013948 | Eip93F |
| AM02585 |  |  |  |  |
| AM02588 |  |  |  |  |
| AM02617 |  |  |  |  |
| AM02625 |  |  |  |  |
| AM02626 | GB19418 | NM_001011574.1 | FBgn0001112 | Gld |
| AM02635 |  |  |  |  |
| AM02669 | GB10041 | XM_001119847.1 | FBgn0031990 | CG8552 |
| AM02687 |  |  |  |  |
| AM02706 | GB10078 | XM_396244.1 | FBgn0033046 | CG14470 |
| AM02720 | GB10092 | XM_395179.2 | FBgn0027321 | l(1)G0060 |
| AM02725 | GB10097 | XM_624071.2 | FBgn0036685 | CG6664 |
| AM02742 | GB10114 | XM_393807.3 | FBgn0043364 | cbt |
| AM02746 | GB10118 | XM_391857.2 | FBgn0040297 | Nhe2 |
| AM02757 | GB10294 | XM_001121693.1 | FBgn0032192 | CG5731 |
| AM02762 | GB10134 | XM_001121120.1 | FBgn0003638 | su(w[a]) |
| AM02764 | GB10136 | XM_001122160.1 | FBgn0060296 | pain |
| AM02766 | GB10138 | XM_392309.2 | FBgn0016920 | nompC |
| AM02768 | GB10140 | XM_395276.3 | FBgn0037305 | CG12173 |
| AM02814 | GB10187 |  | FBgn0003175 | px |
| AM02832 | GB10205 | XM_393414.3 | FBgn0002478 | l(3)IX-14 |
| AM02834 | GB10207 | XM_392642.2 | FBgn0037674 | Vps16A |
| AM02855 | GB10228 | XM_001121130.1 | FBgn0029975 | CG1444 |
| AM02863 | GB10236 | XM_001122939.1 | FBgn0022786 | Hira |
| AM02864 | GB10237 | XR_014928.1 | FBgn0019652 | sif |
| AM02872 | GB10245 | XM_001120581.1 | FBgn0039767 | CG2218 |
| AM02897 | GB30104 | XM_001122389.1 | FBgn0035917 | CG6416 |
| AM02910 | GB10280 | XM_393616.3 | FBgn0024728 | Slip1 |
| AM02926 | GB10296 | XM_393619.2 | FBgn0038296 | CG6752 |
| AM02936 | GB10306 | XM_395966.2 | FBgn0032934 | CG8679 |
| AM02954 | GB10324 |  | FBgn0052529 | CG32529 |
| AM02967 | GB10337 | XM_392578.3 | FBgn0017645 | Ank2 |
| AM02992 | GB10362 | XM_001120922.1 | FBgn0038402 | Fer2 |
| AM03005 | GB10376 | XM_001120777.1 | FBgn0036741 | CG7510 |
| AM03021 | GB10393 | XM_001122723.1 | FBgn0032793 | CG10189 |
| AM03024 | GB10396 | XM_393222.3 | FBgn0030141 | Gga |
| AM03065 | GB10437 |  | FBgn0004509 | Fur1 |
| AM03091 | GB10464 |  | FBgn0039357 | CG4743 |
| AM03092 | GB10465 |  | FBgn0036141 | srt |
| AM03116 | GB10489 | XM_394381.3 | FBgn0032946 | nrv3 |
| AM03126 | GB10500 | XR_014888.1 | FBgn0021874 | Nle |
| AM03128 | GB10502 |  | FBgn0052937 | CG32937 |
| AM03132 |  |  |  |  |
| AM03136 | GB10509 | XM_396657.3 | FBgn0027335 | l(1)G0003 |
| AM03176 | GB10549 |  | FBgn0003513 | ss |
| AM03181 | GB10554 | XM_397338.2 | FBgn0031255 | BBS8 |
| AM03187 | GB10560 | NM_001011628.1 | FBgn0000557 | Ef1alpha100E |
| AM03198 | GB10571 |  | FBgn0030627 | gce |
| AM03208 | GB10581 | XM_625119.2 | FBgn0023536 | CG3156 |
| AM03211 |  |  |  |  |
| AM03234 | GB10607 | XM_624381.2 | FBgn0028421 | Kap3 |
| AM03236 | GB10608 | XM_393376.3 | FBgn0017572 | Mo25 |
| AM03248 | GB10622 | NM_001011599.1 | FBgn0039896 | yellow-h |
| AM03251 | GB10625 | XM_392434.3 | FBgn0034389 | Mctp |
| AM03283 | GB10656 |  | FBgn0020309 | crol |
| AM03284 | GB10657 | XM_392432.3 | FBgn0015808 | ScpX |
| AM03295 | GB10669 | XM_001122174.1 | FBgn0034743 | RpS16 |
| AM03333 | GB10708 | XM_397526.3 | FBgn0052055 | CG32055 |
| AM03347 |  |  |  |  |
| AM03370 | GB10745 | XR_015068.1 | FBgn0052103 | CG32103 |
| AM03391 | GB10766 | XM_625068.2 | FBgn0000464 | Lar |
| AM03396 | GB10771 | XM_394922.3 | FBgn0010053 | Jheh1 |
| AM03409 | GB10784 | XM_396337.2 | FBgn0034371 | SP2637 |
| AM03420 | GB10795 | XM_392277.3 | FBgn0033717 | CG8839 |
| AM03421 | GB10796 | XM_625214.1 | FBgn0028693 | Rpn12 |
| AM03434 | GB10809 | XM_395880.3 | FBgn0036446 | CG9384 |
| AM03440 | GB10815 | XM_624489.2 | FBgn0032955 | CG2201 |
| AM03457 | GB10832 | XM_393699.3 | FBgn0031673 | CG31650 |
| AM03470 | GB10845 | XM_393167.3 | FBgn0024734 | PRL-1 |
| AM03480 |  |  |  |  |
| AM03485 |  |  |  |  |
| AM03494 | GB10869 | NM_001011600.1 | FBgn0002565 | Lsp2 |
| AM03506 | GB10881 | XM_001121324.1 | FBgn0031498 | CG17260 |
| AM03512 | GB10887 | XM_001121440.1 | FBgn0045035 | tefu |
| AM03528 | GB10903 | NM_001011587.1 | FBgn0002626 | RpL32 |
| AM03530 | GB10905 | XM_397272.3 | FBgn0015032 | Cyp4c3 |
| AM03560 | GB10935 | XM_393997.1 | FBgn0030346 | CG11802 |
| AM03617 | GB10995 | XM_001121420.1 | FBgn0053205 | CG33205 |
| AM03618 | GB10995 | XM_001121420.1 | FBgn0053205 | CG33205 |
| AM03623 | GB10999 | XM_392117.3 | FBgn0034537 | DMAP1 |
| AM03624 |  |  |  |  |
| AM03628 | GB30096 | XM_624473.2 | FBgn0010762 | simj |
| AM03644 | GB11020 | XM_395466.2 | FBgn0036031 | CG6761 |
| AM03646 | GB11022 | NM_001014429.1 | FBgn0039896 | yellow-h |
| AM03647 | GB11023 | XM_392731.3 | FBgn0027571 | CG3523 |
| AM03648 | GB11024 | XM_625104.2 | FBgn0031736 | CG11030 |
| AM03731 | GB11107 | XM_393409.3 | FBgn0027568 | CG5366 |
| AM03764 | GB30057 | XM_001121077.1 | FBgn0036495 | CG33259 |
| AM03765 | GB11141 |  | FBgn0005654 | lat |
| AM03767 | GB11143 | XM_624383.1 | FBgn0024994 | Csat |
| AM03777 | GB11153 | XM_001121327.1 | FBgn0038257 | smp-30 |
| AM03792 | GB11168 |  | FBgn0003499 | sr |
| AM03795 | GB11171 | XM_623605.2 | FBgn0020503 | CLIP-190 |
| AM03811 | GB11187 | XM_624300.1 | FBgn0025716 | Bap55 |
| AM03820 | GB11197 |  | FBgn0013813 | Dhc98D |
| AM03822 | GB11199 | XM_001120593.1 | FBgn0013988 | Strn-Mlck |
| AM03826 | GB11203 | XM_001120648.1 | FBgn0036064 | Ard1 |
| AM03843 | GB11220 | XM_624755.2 | FBgn0037533 | CG2791 |
| AM03848 | GB11225 | XM_623712.2 | FBgn0029737 | CG6903 |
| AM03854 | GB11231 | XM_394846.2 | FBgn0052495 | CG32495 |
| AM03879 | GB11256 | XM_001122900.1 | FBgn0032029 | CG17292 |
| AM03883 | GB11260 | XM_393370.3 | FBgn0028690 | Rpn5 |
| AM03884 | GB11261 | XM_393332.3 | FBgn0039348 | CG4673 |
| AM03885 | GB11262 | XM_001120044.1 | FBgn0032013 | Scgalpha |
| AM03899 | GB11276 |  | FBgn0051183 | CG31183 |
| AM03911 | GB11288 | XR_014873.1 | FBgn0004242 | syt |
| AM03972 |  |  |  |  |
| AM03980 | GB11359 | XM_001120625.1 | FBgn0027083 | Aats-met |
| AM03982 | GB11361 | XM_623084.2 | FBgn0031645 | CG3036 |
| AM04023 | GB11402 |  | FBgn0024273 | WASp |
| AM04025 | GB11404 | XM_001120829.1 | FBgn0003744 | trc |
| AM04029 | GB11408 | XM_393472.3 | FBgn0043884 | mask |
| AM04053 |  |  |  |  |
| AM04060 |  |  |  |  |
| AM04066 | GB11445 | XM_396120.2 | FBgn0033252 | CG12769 |
| AM04072 | GB11451 | XM_624637.2 | FBgn0032731 | CG10641 |
| AM04073 | GB11452 | XM_396755.3 | FBgn0053208 | MICAL |
| AM04098 | GB11477 | XM_623860.1 | FBgn0040211 | hgo |
| AM04102 | GB11481 |  | FBgn0027499 | CG12340 |
| AM04113 | GB11493 |  | FBgn0037487 | CG14608 |
| AM04117 | GB11497 | XM_397449.2 | FBgn0035895 | Unr |
| AM04133 | GB11513 | XM_001121033.1 | FBgn0036340 | SRm160 |
| AM04138 | GB30227_2 | XM_623959.2 | FBgn0000261 | Cat |
| AM04186 | GB11566 | XM_623754.2 | FBgn0004102 | oc |
| AM04187 | GB11567 | XM_623735.2 | FBgn0024754 | Flo |
| AM04196 | GB11576 | XM_624016.2 | FBgn0031977 | CG7380 |
| AM04205 |  |  |  |  |
| AM04229 | GB11609 | XM_394021.1 | FBgn0039352 | CG5053 |
| AM04245 | GB11625 | XM_394067.2 | FBgn0028679 | Sema-5c |
| AM04256 | GB11636 | XM_395139.1 | FBgn0017549 | Ric |
| AM04269 | GB11649 | XM_392475.3 | FBgn0053080 | CG33080 |
| AM04283 | GB11663 | XM_624858.1 | FBgn0020392 | Nmt |
| AM04289 | GB11669 |  | FBgn0034248 | CG14483 |
| AM04309 | GB11689 | XR_014995.1 | FBgn0011300 | babo |
| AM04363 | GB11744 | XM_396117.3 | FBgn0039541 | CG12876 |
| AM04409 |  |  |  |  |
| AM04423 | GB11804 | XM_623873.2 | FBgn0027592 | MED15 |
| AM04427 | GB11808 | XM_393986.3 | FBgn0036240 | CG6928 |
| AM04434 | GB11815 | XM_393006.3 | FBgn0035147 | CG12030 |
| AM04435 |  |  |  |  |
| AM04436 | GB11817 | XM_001120108.1 | FBgn0020762 | Atet |
| AM04439 | GB11820 | XM_393343.3 | FBgn0016672 | Ipp |
| AM04456 |  |  |  |  |
| AM04460 | GB11841 | XM_001123352.1 | FBgn0026438 | Eaat2 |
| AM04461 | GB11842 | XM_392438.3 | FBgn0035677 | CG13293 |
| AM04467 | GB11848 | XM_623072.2 | FBgn0029003 | mab-2 |
| AM04469 | GB11850 | XM_393443.3 | FBgn0036335 | mRpL20 |
| AM04470 | GB11851 | XM_001122102.1 | FBgn0032296 | CG6729 |
| AM04481 | GB11862 | XM_001120733.1 | FBgn0032400 | CG6770 |
| AM04487 | GB11868 | XM_392276.3 | FBgn0037072 | Rab26 |
| AM04500 | GB11882 | XM_393277.2 | FBgn0029937 | CG8300 |
| AM04541 | GB11924 |  | FBgn0002521 | pho |
| AM04550 | GB11933 | XM_392880.3 | FBgn0032597 | CG17904 |
| AM04584 | GB11967 | XM_001121588.1 | FBgn0034271 | CG4996 |
| AM04599 | GB11982 | XM_001120920.1 | FBgn0032811 | CG10268 |
| AM04600 | GB11983 | XM_393561.3 | FBgn0012037 | Ance |
| AM04607 | GB11990 |  | FBgn0029850 | CG14446 |
| AM04609 | GB11992 | XM_001121180.1 | FBgn0034982 | CG4065 |
| AM04610 | GB11993 | XM_001121119.1 | FBgn0034360 | CG10927 |
| AM04618 | GB12001 | XM_623485.2 | FBgn0051666 | CG31666 |
| AM04620 | GB12003 | XM_001122998.1 | FBgn0037794 | CG6254 |
| AM04621 | GB12004 | XM_001122040.1 | FBgn0001291 | Jra |
| AM04626 | GB12009 | XM_001122675.1 | FBgn0030941 | wgn |
| AM04639 |  |  |  |  |
| AM04650 | GB12033 | XM_394765.3 | FBgn0052687 | CG32687 |
| AM04676 | GB12059 | XM_001122049.1 | FBgn0031781 | Arc-p20 |
| AM04702 | GB12085 |  | FBgn0020439 | fau |
| AM04707 | GB12090 | XM_624246.2 | FBgn0038224 | CG3321 |
| AM04711 | GB12094 | XM_001121196.1 | FBgn0005630 | lola |
| AM04712 | GB12094 | XM_001121196.1 | FBgn0005630 | lola |
| AM04713 | GB12094 | XM_001121196.1 | FBgn0005630 | lola |
| AM04714 | GB12094 | XM_001121196.1 | FBgn0005630 | lola |
| AM04734 | GB12113 | XM_623722.2 | FBgn0004363 | porin |
| AM04739 | GB12118 | XM_394555.3 | FBgn0038950 | CG5382 |
| AM04828 |  |  |  |  |
| AM04830 | GB12210 | XM_001122336.1 | FBgn0038492 | CG4090 |
| AM04832 | GB30215 | XM_001119924.1 | FBgn0001297 | kay |
| AM04837 | GB12218 | XM_001122184.1 | FBgn0053864 | His1:CG33864 |
| AM04843 | GB12224 | XM_395190.3 | FBgn0024983 | CG4293 |
| AM04865 |  |  |  |  |
| AM04880 | GB30566 |  | FBgn0037836 | CG14692 |
| AM04906 | GB12287 | XM_396705.2 | FBgn0000180 | bib |
| AM04926 | GB12307 | XM_392283.3 | FBgn0035953 | CG5087 |
| AM04943 | GB12324 | XM_394225.3 | FBgn0014024 | Rnp4F |
| AM04953 | GB12334 | XR_015063.1 | FBgn0051064 | CG31064 |
| AM04954 | GB12335 | XM_393488.3 | FBgn0032197 | CG5694 |
| AM04958 | GB12339 | XM_394432.3 | FBgn0011817 | nmo |
| AM04964 | GB12346 | XM_392132.3 | FBgn0031816 | CG16947 |
| AM04975 | GB12357 | XM_394795.2 | FBgn0029157 | ssh |
| AM04976 | GB12358 | XM_001121733.1 | FBgn0034564 | CG9344 |
| AM04978 | GB12360 | XM_393904.3 | FBgn0017558 | Pdk |
| AM04979 | GB12361 | XM_001119826.1 | FBgn0032518 | RpL24 |
| AM04987 | GB12369 |  | FBgn0038282 | dpr9 |
| AM04989 | GB12371 | XM_394313.3 | FBgn0025814 | Mgstl |
| AM05014 | GB12396 | XM_623946.2 | FBgn0000455 | Dip-C |
| AM05036 | GB12419 | XM_625017.2 | FBgn0024332 | Mcm3 |
| AM05067 |  |  |  |  |
| AM05089 | GB12472 | XM_001122127.1 | FBgn0020309 | crol |
| AM05111 | GB12494 |  | FBgn0028878 | CG15269 |
| AM05112 | GB12495 | XM_625286.2 | FBgn0033603 | CG13214 |
| AM05132 | GB12515 | XM_392635.3 | FBgn0038055 | trus |
| AM05145 | GB12529 | NM_001040263.1 | FBgn0031148 | CG1753 |
| AM05153 | GB12537 | XM_001122033.1 | FBgn0038540 | CG14321 |
| AM05168 | GB12553 |  | FBgn0039924 | CG17471 |
| AM05177 | GB12561 | XM_001123021.1 | FBgn0033714 | garz |
| AM05183 | GB12567 | XM_624222.2 | FBgn0027348 | bgm |
| AM05189 | GB12573 | XM_393545.3 | FBgn0026708 | l(1)G0030 |
| AM05196 | GB12580 | XM_393494.2 | FBgn0030442 | CG15720 |
| AM05202 | GB12586 | XM_623828.2 | FBgn0014002 | Pdi |
| AM05215 |  |  |  |  |
| AM05255 |  |  |  |  |
| AM05319 | GB12704 | XM_001121319.1 | FBgn0037536 | CG2698 |
| AM05338 | GB12723 | XM_396382.2 | FBgn0023527 | CG3071 |
| AM05345 | GB12730 |  | FBgn0039347 | CG5071 |
| AM05351 | GB12736 | XM_001122401.1 | FBgn0039385 | CG5913 |
| AM05356 | GB12741 | XM_623081.2 | FBgn0012036 | Aldh |
| AM05361 | GB12746 | XM_392803.3 | FBgn0052133 | CG32133 |
| AM05394 | GB12779 | XM_394467.1 | FBgn0027936 | vih |
| AM05395 | GB12780 | XM_396405.2 | FBgn0023521 | CG3587 |
| AM05426 | GB12811 | XM_393452.3 | FBgn0003065 | CG2150 |
| AM05446 | GB12832 |  | FBgn0035254 | CG7974 |
| AM05454 | GB12840 | XM_001121454.1 | FBgn0003498 | sqd |
| AM05460 | GB12846 |  | FBgn0052048 | CG32048 |
| AM05467 | GB12853 | XM_392099.3 | FBgn0015609 | CadN |
| AM05480 | GB12866 | XM_392690.3 | FBgn0010348 | Arf79F |
| AM05484 | GB12871 | XM_624586.2 | FBgn0000253 | Cam |
| AM05497 | GB12884 | XM_392202.3 | FBgn0035142 | CG17090 |
| AM05524 | GB12911 | XM_001121342.1 | FBgn0040334 | Tsp3A |
| AM05534 | GB12922 | XM_001119846.1 | FBgn0061209 | His2B:CG17949 |
| AM05570 | GB12957 | XM_392116.3 | FBgn0038492 | CG4090 |
| AM05602 | GB12991 | XM_624164.2 | FBgn0001197 | His2Av |
| AM05608 | GB12997 | XR_015099.1 | FBgn0035950 | CG5288 |
| AM05613 | GB13002 | XM_396099.2 | FBgn0029853 | CG3781 |
| AM05614 | GB13003 | XM_393066.3 | FBgn0005648 | Pabp2 |
| AM05615 | GB13004 | XM_392381.3 | FBgn0037303 | CG12163 |
| AM05627 | GB13016 | XM_394454.2 | FBgn0031275 | GABA-B-R3 |
| AM05638 |  |  |  |  |
| AM05655 | GB13045 | XM_395766.3 | FBgn0039349 | CG4685 |
| AM05658 | GB13048 | XM_624089.2 | FBgn0038811 | CG4159 |
| AM05710 | GB13102 | XM_392585.3 | FBgn0031991 | CG8506 |
| AM05726 | GB13118 | XM_001120264.1 | FBgn0010278 | Ssrp |
| AM05728 | GB13120 | XM_392335.3 | FBgn0036485 | FucTA |
| AM05741 | GB13133 | XM_001120919.1 | FBgn0026314 | Ugt35b |
| AM05764 | GB13155 | XM_396831.2 | FBgn0030884 | CG6847 |
| AM05827 | GB13219 | XM_001120341.1 | FBgn0041607 | asparagine-synthetase |
| AM05893 | GB13286 | XM_624563.2 | FBgn0039115 | CG10214 |
| AM05897 | GB13290 | XR_015057.1 | FBgn0039257 | tnc |
| AM05926 | GB13322 | XM_396686.2 | FBgn0034057 | CG8314 |
| AM05928 | GB13324 | XM_395013.3 | FBgn0010391 | Gtp-bp |
| AM05929 | GB13325 | XM_001120200.1 | FBgn0011695 | PebIII |
| AM05931 | GB13327 | XM_396514.3 | FBgn0003041 | pbl |
| AM05934 | GB13330 | XM_001122158.1 | FBgn0038818 | Nep4 |
| AM05946 |  |  |  |  |
| AM05950 | GB13346 |  | FBgn0038747 | RhoGAP92B |
| AM05960 |  |  |  |  |
| AM05971 | GB13368 | XM_393423.3 | FBgn0030737 | CG9914 |
| AM05998 | GB13395 | XM_392802.3 | FBgn0028475 | CG10221 |
| AM05999 | GB13396 |  | FBgn0030576 | CG15890 |
| AM06028 | GB13424 | XM_397443.3 | FBgn0011704 | RnrS |
| AM06045 | GB13441 | XM_623817.2 | FBgn0040295 | Ogt |
| AM06051 | GB13447 |  | FBgn0051224 | CG31224 |
| AM06062 | GB13458 | XM_624158.2 | FBgn0025117 | und |
| AM06086 | GB13483 | XM_393042.3 | FBgn0051550 | CG31550 |
| AM06095 | GB13493 | NM_001011606.1 | FBgn0014019 | Rh5 |
| AM06098 | GB13496 | XM_394683.3 | FBgn0034357 | Rgk2 |
| AM06099 | GB13497 | XM_394801.3 | FBgn0026620 | tacc |
| AM06103 | GB13501 | XM_001122557.1 | FBgn0034909 | CG4797 |
| AM06117 | GB13515 | XM_394615.3 | FBgn0038745 | CG4538 |
| AM06118 | GB13516 | XM_393425.2 | FBgn0025593 | Glut1 |
| AM06128 | GB13526 | XM_001120056.1 | FBgn0037172 | CG6914 |
| AM06135 | GB13534 | XM_001121402.1 | FBgn0031300 | CG4644 |
| AM06137 | GB13536 | XM_624340.2 | FBgn0037643 | CG11963 |
| AM06152 | GB13551 | XM_396168.3 | FBgn0035086 | CG12851 |
| AM06176 | GB13575 | XM_001120885.1 | FBgn0025615 | torp4a |
| AM06178 | GB13577 | XM_392075.3 | FBgn0026086 | Adar |
| AM06192 | GB13592 |  | FBgn0010235 | Klc |
| AM06194 | GB13594 | NM_001011651.1 | FBgn0013348 | TpnC41C |
| AM06203 | GB13603 | XM_392582.3 | FBgn0036137 | CG7628 |
| AM06204 | GB13604 | XM_393623.3 | FBgn0027575 | GABA-B-R2 |
| AM06206 |  |  |  |  |
| AM06220 | GB13621 | XM_623633.2 | FBgn0052250 | CG32250 |
| AM06224 | GB13625 |  | FBgn0037238 | CG1090 |
| AM06255 | GB13657 | XM_393596.3 | FBgn0039214 | CG5794 |
| AM06267 | GB13669 |  | FBgn0030940 | CG15040 |
| AM06271 | GB13673 | XM_394743.2 | FBgn0030387 | Pkcdelta |
| AM06280 | GB13682 | XM_001122632.1 | FBgn0032633 | CG6860 |
| AM06281 | GB13683 | XM_392535.3 | FBgn0040206 | krz |
| AM06298 | GB13700 | XM_001122561.1 | FBgn0002741 | Mhc |
| AM06300 | GB13702 | XM_394417.3 | FBgn0034735 | CG4610 |
| AM06315 | GB13717 | XM_001122365.1 | FBgn0034695 | CG13503 |
| AM06342 | GB13743 | XM_001121853.1 | FBgn0032397 | Tom70 |
| AM06363 | GB13764 | XM_393750.3 | FBgn0034602 | CG15658 |
| AM06368 | GB13769 | XM_001121781.1 | FBgn0039555 | mRpS22 |
| AM06370 | GB13771 | XM_623889.2 | FBgn0050296 | CG30296 |
| AM06388 | GB13789 | NM_001011622.1 | FBgn0039896 | yellow-h |
| AM06389 | GB13790 |  | FBgn0028872 | CG18095 |
| AM06391 | GB13792 | XM_624751.2 | FBgn0004797 | mdy |
| AM06400 | GB13801 | XM_001122146.1 | FBgn0038651 | CG14299 |
| AM06422 | GB13823 | XM_623262.2 | FBgn0039920 | CG11360 |
| AM06436 | GB13837 | XM_623602.1 | FBgn0035026 | CG12252 |
| AM06452 | GB13853 | XM_394154.3 | FBgn0024242 | Dys |
| AM06462 |  |  |  |  |
| AM06507 | GB13909 |  | FBgn0035429 | CG12017 |
| AM06516 | GB13918 | XM_624993.2 | FBgn0032285 | CG17108 |
| AM06524 | GB13926 |  | FBgn0004569 | argos |
| AM06525 | GB13927 | XM_625208.2 | FBgn0000662 | fl(2)d |
| AM06589 |  |  |  |  |
| AM06625 | GB14026 | XM_001119961.1 | FBgn0027108 | inx2 |
| AM06645 | GB30234 | XM_624189.2 | FBgn0005658 | Ets65A |
| AM06650 | GB14051 | XM_393736.2 | FBgn0031824 | CG9547 |
| AM06656 | GB14057 | XM_396289.2 | FBgn0031252 | CG13690 |
| AM06657 |  |  |  |  |
| AM06676 | GB14074 | XM_001121228.1 | FBgn0037656 | CG11986 |
| AM06712 |  |  |  |  |
| AM06718 | GB14118 | XM_393542.3 | FBgn0024555 | flfl |
| AM06762 | GB14162 | XM_624216.2 | FBgn0030788 | CG4756 |
| AM06807 | GB14208 | XM_624177.2 | FBgn0052627 | CG32627 |
| AM06852 | GB14253 | XM_394497.3 | FBgn0036500 | CG7275 |
| AM06872 | GB14273 | XM_001120035.1 | FBgn0002932 | neur |
| AM06886 | GB14286 |  | FBgn0031273 | CG2839 |
| AM06892 | GB14292 | XM_393053.2 | FBgn0052600 | dpr8 |
| AM06941 | GB14342 | XM_624197.2 | FBgn0040337 | CG3021 |
| AM06954 | GB14356 | XM_393087.3 | FBgn0034372 | Gint3 |
| AM06972 | GB14374 | XM_001120576.1 | FBgn0015276 | Pcmt |
| AM06980 | GB14382 | XM_624192.2 | FBgn0013997 | Nrx-IV |
| AM07015 | GB14417 | XM_001122499.1 | FBgn0034245 | CG14482 |
| AM07023 |  |  |  |  |
| AM07032 | GB14434 | XM_397411.3 | FBgn0030608 | Lsd-2 |
| AM07033 | GB14435 | XM_001120006.1 | FBgn0011296 | l(2)efl |
| AM07063 |  |  |  |  |
| AM07086 | GB14488 | XM_393828.2 | FBgn0040207 | kat80 |
| AM07127 | GB14529 | XM_392429.3 | FBgn0003138 | Ptp61F |
| AM07128 | GB14529 | XM_392429.3 | FBgn0003138 | Ptp61F |
| AM07140 | GB14541 | XM_393011.3 | FBgn0052393 | dikar |
| AM07154 | GB14556 | XM_623782.2 | FBgn0035364 | CG14950 |
| AM07169 | GB14572 | XM_392549.3 | FBgn0030234 | CG15211 |
| AM07186 | GB14589 | XM_001121843.1 | FBgn0039648 | CG14515 |
| AM07189 | GB14592 | XM_624536.2 | FBgn0033917 | CG8503 |
| AM07205 | GB14608 | XM_623717.2 | FBgn0032221 | CG5375 |
| AM07274 | GB14677 | XM_395924.2 | FBgn0039241 | CG11089 |
| AM07281 | GB14684 |  | FBgn0051522 | CG31522 |
| AM07292 | GB14695 | XM_397223.3 | FBgn0030230 | Rph |
| AM07299 | GB14702 | XM_001119932.1 | FBgn0038588 | CG7156 |
| AM07307 | GB14710 | XM_393931.1 | FBgn0038738 | CG4572 |
| AM07337 | GB14740 | XM_625249.2 | FBgn0032638 | CG6639 |
| AM07346 | GB14749 | XM_392524.3 | FBgn0024320 | NPC1 |
| AM07360 | GB14765 | XM_396662.1 | FBgn0026147 | CG16833 |
| AM07367 | GB14772 | XM_396908.3 | FBgn0003501 | Src64B |
| AM07368 | GB14773 | XM_392567.3 | FBgn0052663 | CG32663 |
| AM07389 | GB14794 | XM_396616.2 | FBgn0028992 | sds22 |
| AM07405 | GB14810 | XM_001120586.1 | FBgn0022787 | Hel89B |
| AM07409 | GB14814 | XM_001121233.1 | FBgn0042132 | CG18809 |
| AM07411 | GB14816 | XM_001122257.1 | FBgn0034096 | CG7786 |
| AM07424 | GB14829 | XM_391846.3 | FBgn0035981 | CG4452 |
| AM07470 | GB14876 | XM_625025.2 | FBgn0035037 | CG3579 |
| AM07474 | GB14880 | XR_014875.1 | FBgn0040002 | CG17683 |
| AM07482 | GB14888 | NM_001011579.1 | FBgn0039896 | yellow-h |
| AM07488 | GB14894 | XM_395088.3 | FBgn0053002 | mRpL27 |
| AM07512 | GB14921 |  | FBgn0025639 | Suv4-20 |
| AM07520 | GB14929 | XM_623051.2 | FBgn0052066 | CG32066 |
| AM07541 | GB14950 | XM_393674.2 | FBgn0001301 | kel |
| AM07546 | GB14955 | XM_623701.2 | FBgn0028658 | adat |
| AM07549 | GB14958 | XR_015059.1 | FBgn0020615 | SelD |
| AM07563 | GB14971 |  | FBgn0051072 | Lerp |
| AM07565 |  |  |  |  |
| AM07579 | GB14988 | XM_623305.2 | FBgn0051712 | CG31712 |
| AM07583 | GB14992 | XM_001122036.1 | FBgn0050197 | CG30197 |
| AM07591 | GB15000 | XM_396801.3 | FBgn0010851 | sgl |
| AM07607 | GB15016 | XM_393090.3 | FBgn0001218 | Hsc70-3 |
| AM07609 | GB15018 | XM_001120243.1 | FBgn0029167 | Hml |
| AM07624 | GB15033 | XR_015023.1 | FBgn0052705 | CG32705 |
| AM07631 | GB15040 | XM_391902.3 | FBgn0052131 | CG32131 |
| AM07675 |  |  |  |  |
| AM07677 | GB15086 | XM_393352.3 | FBgn0040286 | SC35 |
| AM07682 | GB15091 | XM_397552.3 | FBgn0004870 | bab1 |
| AM07683 | GB15092 | XM_397284.3 | FBgn0036928 | Tom20 |
| AM07697 | GB15106 | XM_001120136.1 | FBgn0034539 | CG11159 |
| AM07707 | GB15117 | XM_392463.3 | FBgn0000303 | Cha |
| AM07753 | GB15163 | XM_001123301.1 | FBgn0032713 | CG17323 |
| AM07790 | GB15201 | XM_001120484.1 | FBgn0039296 | CG10420 |
| AM07800 |  |  |  |  |
| AM07823 |  |  |  |  |
| AM07826 | GB15237 | XM_394796.3 | FBgn0083975 | CG34139 |
| AM07837 | GB15248 | XM_001121921.1 | FBgn0041174 | Vhl |
| AM07846 | GB15257 | XM_625193.2 | FBgn0039003 | wfs1 |
| AM07851 | GB15262 | XM_393729.2 | FBgn0083945 | CG34109 |
| AM07863 |  |  |  |  |
| AM07868 | GB15279 | XM_001120221.1 | FBgn0030940 | CG15040 |
| AM07873 | GB15283 | XM_001122489.1 | FBgn0035720 | CG10077 |
| AM07875 | GB15285 | XM_395028.3 | FBgn0050497 | CG30497 |
| AM07894 | GB15304 | XM_396744.3 | FBgn0033886 | CG13349 |
| AM07912 | GB15324 | XM_625100.2 | FBgn0033392 | CG8027 |
| AM07921 | GB15333 | XM_624887.2 | FBgn0042094 | Adk3 |
| AM07931 | GB15343 | XM_624929.1 | FBgn0038196 | CG9922 |
| AM07946 | GB15358 |  | FBgn0040913 | CG15785 |
| AM07969 | GB15381 | XM_001122064.1 | FBgn0052677 | CG32677 |
| AM07975 | GB15387 |  | FBgn0001280 | janA |
| AM07978 | GB15390 | XM_001122757.1 | FBgn0030940 | CG15040 |
| AM07979 | GB15391 | XM_001121069.1 | FBgn0030839 | CG5613 |
| AM08024 | GB15437 | XM_624452.2 | FBgn0033837 | CG17034 |
| AM08041 | GB15454 |  | FBgn0035497 | CG14995 |
| AM08047 | GB15460 | NM_001040206.1 | FBgn0034470 | Obp56d |
| AM08067 | GB15481 | XM_396160.3 | FBgn0031640 | CG11926 |
| AM08088 | GB15503 | XM_392071.3 | FBgn0003279 | RpL4 |
| AM08140 | GB15555 | XM_001120138.1 | FBgn0034223 | CG6522 |
| AM08191 | GB15607 |  | FBgn0030619 | CG15029 |
| AM08222 | GB15638 | XM_394206.3 | FBgn0030805 | wus |
| AM08227 | GB15643 | XM_391982.3 | FBgn0033288 | pdm3 |
| AM08245 | GB15662 | XM_001120613.1 | FBgn0030073 | CG10962 |
| AM08247 | GB15664 | XM_396777.3 | FBgn0004167 | kst |
| AM08255 | GB15672 |  | FBgn0036316 | CG10960 |
| AM08260 | GB15677 | XM_392392.3 | FBgn0039776 | PH4alphaEFB |
| AM08262 | GB15679 | XM_394755.3 | FBgn0028408 | Rep2 |
| AM08292 | GB15709 | XM_625205.2 | FBgn0011712 | Snap |
| AM08306 | GB15723 | XM_624240.2 | FBgn0030789 | Rrp45 |
| AM08327 | GB15745 | XM_392052.3 | FBgn0036975 | CG5618 |
| AM08345 | GB15763 | XM_001122793.1 | FBgn0032042 | CG13398 |
| AM08374 | GB15792 | XM_391860.3 | FBgn0004587 | B52 |
| AM08407 | GB15825 | XM_001122550.1 | FBgn0033951 | CG10139 |
| AM08433 | GB15851 | XM_392264.2 | FBgn0052447 | CG32447 |
| AM08466 |  |  |  |  |
| AM08483 | GB15902 | XM_624680.2 | FBgn0025186 | ari-2 |
| AM08490 | GB15909 | XM_395295.3 | FBgn0005427 | ewg |
| AM08493 | GB15912 | XM_624982.2 | FBgn0039647 | CG14509 |
| AM08515 | GB15934 | XM_001122451.1 | FBgn0034520 | CG13424 |
| AM08555 | GB15974 | XM_623761.2 | FBgn0010825 | Gug |
| AM08562 | GB15981 |  | FBgn0023518 | trr |
| AM08572 | GB15990 | NM_001011636.1 | FBgn0037913 | CG6783 |
| AM08593 | GB16012 | XR_014871.1 | FBgn0003244 | rg |
| AM08625 | GB16044 | XM_001120488.1 | FBgn0037142 | CG14562 |
| AM08641 | GB16060 | XM_394552.3 | FBgn0037736 | CG12950 |
| AM08642 | GB30579_2 |  | FBgn0019990 | Gcn2 |
| AM08652 | GB16071 | XM_397451.3 | FBgn0020510 | Abi |
| AM08681 | GB16101 | XM_001122140.1 | FBgn0053229 | CG33229 |
| AM08740 | GB16162 | XM_394745.3 | FBgn0031992 | CG8498 |
| AM08746 | GB16168 | XM_001121359.1 | FBgn0039257 | tnc |
| AM08796 | GB16216 | XM_395372.3 | FBgn0035833 | CG7565 |
| AM08843 | GB16263 | XM_393341.3 | FBgn0027356 | Amph |
| AM08874 | GB16294 | XM_624795.2 | FBgn0034603 | Glycogenin |
| AM08883 | GB16303 | XM_623791.2 | FBgn0051450 | mRpS18A |
| AM08895 | GB16315 | XM_394997.3 | FBgn0037202 | Ssl1 |
| AM08914 |  |  |  |  |
| AM08915 | GB16335 | XM_394187.3 | FBgn0016685 | Nlp |
| AM08926 | GB16346 | XM_623068.2 | FBgn0003204 | ras |
| AM08934 | GB16354 | XM_392446.3 | FBgn0033624 | CG12384 |
| AM08944 | GB16363 | XM_392491.3 | FBgn0036943 | CG7323 |
| AM08952 | GB16371 | XM_623438.1 | FBgn0033570 | CG7712 |
| AM08958 | GB16377 | NM_001011597.1 | FBgn0026438 | Eaat2 |
| AM08960 | GB16379 | XM_396628.3 | FBgn0011725 | twin |
| AM08981 | GB16400 | XM_392069.3 | FBgn0015331 | abs |
| AM08987 | GB16406 | XM_001122973.1 | FBgn0030854 | CG8289 |
| AM08989 | GB16408 | XM_392523.3 | FBgn0033765 | nemy |
| AM08990 | GB16409 | XM_001122322.1 | FBgn0031537 | sec5 |
| AM08991 | GB16412 | XM_395299.3 | FBgn0037874 | Tctp |
| AM08992 | GB16413 | XM_624876.2 | FBgn0032746 | CG10470 |
| AM08998 | GB30339 | XM_396866.3 | FBgn0015234 | HLH106 |
| AM09002 | GB16423 | XM_393927.3 | FBgn0000986 | Fs(2)Ket |
| AM09018 | GB16439 | XM_395716.3 | FBgn0032600 | CG17912 |
| AM09023 | GB16444 | XM_001122942.1 | FBgn0034420 | CG10737 |
| AM09028 | GB16449 | XM_001120474.1 | FBgn0037339 | Pi4KIIalpha |
| AM09038 | GB16459 | NM_001011601.1 | FBgn0039896 | yellow-h |
| AM09052 | GB30393 | XM_001121930.1 | FBgn0013325 | RpL11 |
| AM09067 |  |  |  |  |
| AM09082 | GB16503 | XM_394173.3 | FBgn0051619 | CG31619 |
| AM09116 | GB16537 | XM_394723.3 | FBgn0035720 | CG10077 |
| AM09121 | GB16542 | XM_396017.2 | FBgn0038504 | Sur-8 |
| AM09157 |  |  |  |  |
| AM09197 | GB16619 | XM_393860.3 | FBgn0027570 | Nep2 |
| AM09209 | GB30075 |  | FBgn0039728 | CG7896 |
| AM09211 | GB16633 | XM_392959.3 | FBgn0002989 | okr |
| AM09212 |  |  |  |  |
| AM09241 | GB16663 | XM_394258.3 | FBgn0036309 | CG10971 |
| AM09264 | GB16685 | XM_395490.2 | FBgn0039489 | CG5880 |
| AM09265 |  |  |  |  |
| AM09271 | GB16692 | XM_396566.3 | FBgn0039805 | CG12045 |
| AM09275 | GB16696 |  | FBgn0038531 | CG14325 |
| AM09279 | GB16700 | XM_001120196.1 | FBgn0033450 | CG12924 |
| AM09388 | GB16811 | XM_392605.3 | FBgn0026238 | gus |
| AM09441 | GB16864 | XM_393806.3 | FBgn0028479 | CG4389 |
| AM09503 | GB16926 | XM_623934.1 | FBgn0015287 | RfC40 |
| AM09504 | GB16927 | XM_397488.3 | FBgn0034797 | nahoda |
| AM09506 |  |  |  |  |
| AM09514 | GB16937 | XM_394995.3 | FBgn0013987 | MAPk-Ak2 |
| AM09529 |  |  |  |  |
| AM09533 | GB16956 | XM_394081.2 | FBgn0000447 | Dhod |
| AM09534 | GB30112 | XM_393799.3 | FBgn0035935 | mfr |
| AM09536 | GB16959 | XM_624679.2 | FBgn0010226 | GstS1 |
| AM09547 | GB16970 | XM_392595.3 | FBgn0025574 | Pli |
| AM09557 | GB16980 | XM_395330.2 | FBgn0031360 | CG31937 |
| AM09567 | GB16990 | XM_624379.2 | FBgn0024698 | cpsf |
| AM09580 |  |  |  |  |
| AM09601 | GB17025 | XM_623780.1 | FBgn0083228 | Frq2 |
| AM09624 |  |  |  |  |
| AM09636 | GB17060 | XM_392005.3 | FBgn0030364 | CG15735 |
| AM09642 | GB17066 | XM_392396.3 | FBgn0035039 | CG3608 |
| AM09644 | GB17068 | XM_393237.3 | FBgn0037323 | CG2663 |
| AM09645 | GB17069 | XM_392477.3 | FBgn0042693 | PP2A-B' |
| AM09654 | GB30070_2 |  | FBgn0036558 | mib1 |
| AM09659 | GB17084 | XM_001120036.1 | FBgn0000636 | Fas3 |
| AM09662 | GB17087 | XM_393098.1 | FBgn0034501 | CG13868 |
| AM09663 | GB17088 | XM_396435.3 | FBgn0030692 | mRpS30 |
| AM09669 | GB17094 |  | FBgn0029749 | CG15786 |
| AM09674 | GB17099 | XM_395912.3 | FBgn0001978 | stc |
| AM09692 | GB17117 | XM_001120160.1 | FBgn0042713 | Gpi1 |
| AM09693 | GB17118 | XM_001121986.1 | FBgn0032202 | CG18619 |
| AM09698 | GB17123 | XM_394069.3 | FBgn0031224 | CG11454 |
| AM09699 |  |  |  |  |
| AM09708 | GB17133 | XM_001121970.1 | FBgn0000036 | nAcRalpha-96Aa |
| AM09724 | GB17149 | XM_001121890.1 | FBgn0034083 | lbk |
| AM09733 | GB17158 | XM_624101.2 | FBgn0034089 | CG8446 |
| AM09749 | GB17174 | XM_392950.2 | FBgn0034997 | CG3376 |
| AM09755 | GB17180 | XM_001123067.1 | FBgn0032457 | CG15483 |
| AM09781 | GB17206 | XM_624554.2 | FBgn0039756 | CG9743 |
| AM09784 | GB17209 | XM_397270.3 | FBgn0040475 | SH3PX1 |
| AM09789 | GB17214 | XM_397224.2 | FBgn0010470 | Fkbp13 |
| AM09799 | GB17224 | XM_391881.3 | FBgn0035888 | CG7120 |
| AM09801 |  |  |  |  |
| AM09829 | GB17254 | XM_392070.3 | FBgn0032151 | nAcRalpha-30D |
| AM09834 | GB17259 | XM_623301.1 | FBgn0030444 | CG18646 |
| AM09861 | GB17286 | XM_624893.2 | FBgn0011272 | RpL13 |
| AM09862 | GB17287 | XM_397223.3 | FBgn0030230 | Rph |
| AM09876 | GB17301 | XM_623065.2 | FBgn0051140 | CG31140 |
| AM09881 | GB17306 | XM_394216.3 | FBgn0030873 | CG15814 |
| AM09888 | GB17313 |  | FBgn0030109 | CG12121 |
| AM09918 | GB17343 | XM_391845.3 | FBgn0020245 | ttv |
| AM09933 | GB17360 | XM_001121251.1 | FBgn0029152 | Mkrn1 |
| AM09938 | GB17365 | XM_397429.3 | FBgn0003429 | slo |
| AM09945 |  |  |  |  |
| AM09950 | GB17377 | XM_624453.2 | FBgn0037298 | CG2604 |
| AM09953 | GB17380 | XM_393141.3 | FBgn0014163 | fax |
| AM09977 | GB17404 | XM_624878.1 | FBgn0010504 | kermit |
| AM09981 | GB17409 |  | FBgn0036945 | CG6981 |
| AM10037 | GB17466 | XM_625233.2 | FBgn0030034 | CG10555 |
| AM10040 | GB17469 |  | FBgn0013733 | shot |
| AM10041 | GB17470 | XM_625190.1 | FBgn0026582 | CG9418 |
| AM10070 | GB17499 | NM_001010975.1 | FBgn0003360 | sesB |
| AM10092 |  |  |  |  |
| AM10104 | GB17533 | XM_623603.2 | FBgn0034390 | CG15093 |
| AM10107 | GB17536 |  | FBgn0039215 | CG6695 |
| AM10109 |  |  |  |  |
| AM10119 | GB17548 | XM_624867.1 | FBgn0029648 | CG3603 |
| AM10123 | GB17552 | XM_623714.2 | FBgn0028434 | Ercc1 |
| AM10124 | GB17553 | XM_393995.3 | FBgn0037011 | CG4858 |
| AM10135 | GB17563 | XM_392982.3 | FBgn0016070 | smg |
| AM10137 | GB17565 |  | FBgn0051064 | CG31064 |
| AM10148 | GB17576 |  | FBgn0053017 | CG33017 |
| AM10160 |  |  |  |  |
| AM10161 | GB17590 | XM_623846.2 | FBgn0028863 | CG4587 |
| AM10162 | GB17591 | XM_624946.2 | FBgn0003277 | RpII215 |
| AM10164 |  |  |  |  |
| AM10178 | GB17607 |  | FBgn0027864 | Ogg1 |
| AM10179 |  |  |  |  |
| AM10180 |  |  |  |  |
| AM10186 |  |  |  |  |
| AM10193 | GB17619 | XM_624722.2 | FBgn0028969 | deltaCOP |
| AM10238 | GB17664 | XM_392261.3 | FBgn0027600 | CG4778 |
| AM10252 | GB17678 | XM_393929.3 | FBgn0031257 | CG4133 |
| AM10260 | GB17687 | XM_394316.3 | FBgn0016794 | dos |
| AM10281 | GB17708 | XM_001122173.1 | FBgn0031575 | CG3980 |
| AM10298 | GB17725 | XM_623662.2 | FBgn0036570 | CG5222 |
| AM10302 |  |  |  |  |
| AM10312 | GB17739 | XM_396612.3 | FBgn0032702 | CG10376 |
| AM10313 | GB17740 | XM_394711.3 | FBgn0037622 | CG8202 |
| AM10351 | GB17778 | XM_392081.3 | FBgn0033160 | CG11107 |
| AM10373 | GB17801 | XM_397517.3 | FBgn0037734 | CG9448 |
| AM10397 | GB17826 | XM_393723.3 | FBgn0015789 | Rab10 |
| AM10433 | GB17862 | XM_624898.2 | FBgn0030327 | FucT6 |
| AM10436 | GB17865 | XM_393204.3 | FBgn0039505 | CG5934 |
| AM10438 | GB17867 | XM_396828.3 | FBgn0052698 | CG32698 |
| AM10446 | GB17875 | XM_001120077.1 | FBgn0011695 | PebIII |
| AM10453 | GB17882 | XM_392165.3 | FBgn0083962 | CG34126 |
| AM10471 | GB17900 | XM_395852.3 | FBgn0036169 | CG6128 |
| AM10494 | GB17923 | XM_624349.1 | FBgn0051108 | CG31108 |
| AM10504 | GB17931 | XM_623663.2 | FBgn0034145 | CG5065 |
| AM10510 | GB17937 | XM_396062.3 | FBgn0030320 | CG2247 |
| AM10518 | GB17945 | XM_623854.2 | FBgn0004198 | ct |
| AM10529 | GB17956 | XM_392644.3 | FBgn0053525 | CG33525 |
| AM10534 |  |  |  |  |
| AM10535 | GB17962 | XM_392032.3 | FBgn0052638 | CG32638 |
| AM10555 | GB17982 | XM_392419.3 | FBgn0052372 | CG32372 |
| AM10585 | GB18012 | XM_001121306.1 | FBgn0030528 | CG11095 |
| AM10606 | GB18033 | XM_001120644.1 | FBgn0015930 | dpld |
| AM10618 |  |  |  |  |
| AM10628 | GB18056 | XM_394545.3 | FBgn0031322 | CG5001 |
| AM10638 |  |  |  |  |
| AM10679 | GB18109 | XM_624350.2 | FBgn0036182 | CG6084 |
| AM10778 |  |  |  |  |
| AM10787 | GB18221 |  | FBgn0034654 | CG10306 |
| AM10808 | GB18242 | XM_623115.2 | FBgn0001205 | Hmgcr |
| AM10849 | GB18284 | XM_392699.3 | FBgn0010338 | alpha-Man-I |
| AM10854 | GB18289 |  | FBgn0030940 | CG15040 |
| AM10888 |  |  |  |  |
| AM10893 |  |  |  |  |
| AM10908 | GB18344 | XM_001120799.1 | FBgn0037636 | CG9821 |
| AM10931 | GB18367 |  | FBgn0037465 | CG1105 |
| AM10939 | GB18375 | XM_001120538.1 | FBgn0028992 | sds22 |
| AM10977 | GB18414 | XM_393751.3 | FBgn0000024 | Ace |
| AM10982 | GB18419 | XM_397407.2 | FBgn0038501 | CG5319 |
| AM10998 | GB18435 | XM_394243.3 | FBgn0035101 | p130CAS |
| AM11024 | GB18461 | XM_001121766.1 | FBgn0031401 | CG7082 |
| AM11030 |  |  |  |  |
| AM11033 | GB18471 | XM_001120276.1 | FBgn0032223 | GATAd |
| AM11063 | GB18501 | XM_395981.3 | FBgn0025678 | CaBP1 |
| AM11076 | GB18514 |  | FBgn0038341 | CG14869 |
| AM11092 | GB18531 | XM_624972.1 | FBgn0037613 | Cks85A |
| AM11125 |  |  |  |  |
| AM11154 | GB18593 |  | FBgn0037807 | CG6293 |
| AM11156 |  |  |  |  |
| AM11198 | GB18637 | XM_393085.3 | FBgn0011570 | cpb |
| AM11202 |  |  |  |  |
| AM11203 | GB18642 | XM_625293.2 | FBgn0015390 | futsch |
| AM11228 | GB18667 | XM_001119959.1 | FBgn0030038 | CG1440 |
| AM11260 | GB18699 | XM_392656.3 | FBgn0033657 | CG8271 |
| AM11293 | GB30378 | XM_001123170.1 | FBgn0003450 | snk |
| AM11315 | GB18755 | XM_394981.3 | FBgn0004176 | gammaTub23C |
| AM11318 | GB18758 | XM_001121502.1 | FBgn0026433 | Grip128 |
| AM11325 | GB18765 | XM_394739.2 | FBgn0032217 | CG4972 |
| AM11353 | GB18794 | XM_623632.2 | FBgn0038601 | CG18600 |
| AM11364 | GB18806 | XM_001120186.1 | FBgn0053862 | His2A:CG33862 |
| AM11373 | GB18815 | XM_392943.3 | FBgn0003134 | Pp1alpha-96A |
| AM11376 | GB18818 | XM_623990.2 | FBgn0039734 | Tace |
| AM11403 | GB18846 | XM_393310.3 | FBgn0039876 | CG2126 |
| AM11405 |  |  |  |  |
| AM11412 | GB30326 | XM_001120439.1 | FBgn0035445 | CG12014 |
| AM11416 | GB18859 | XM_395547.3 | FBgn0034049 | CG8291 |
| AM11418 | GB18861 | XM_395100.2 | FBgn0036376 | CG10743 |
| AM11450 | GB30325 | XM_001120490.1 | FBgn0039338 | XNP |
| AM11459 | GB18902 | XM_392256.3 | FBgn0013303 | Nca |
| AM11471 | GB18914 | XM_001121927.1 | FBgn0031755 | CG9050 |
| AM11475 | GB18918 |  | FBgn0004606 | zfh1 |
| AM11495 | GB18938 | XM_396698.3 | FBgn0030672 | CG9281 |
| AM11498 | GB18942 |  | FBgn0053513 | Nmdar2 |
| AM11517 |  |  |  |  |
| AM11527 | GB18971 | XM_394701.3 | FBgn0028341 | l(1)G0232 |
| AM11529 | GB18973 | XM_624640.2 | FBgn0025186 | ari-2 |
| AM11537 | GB18981 | XM_392838.3 | FBgn0011655 | Med |
| AM11541 | GB18985 | XM_001121704.1 | FBgn0027509 | CG7261 |
| AM11565 |  |  |  |  |
| AM11573 | GB19017 | NM_001011608.1 | FBgn0032381 | CG14934 |
| AM11586 | GB19031 |  | FBgn0032209 | Hand |
| AM11602 | GB19047 | XM_001123243.1 | FBgn0036648 | CG4098 |
| AM11608 | GB19053 | XM_623498.2 | FBgn0039175 | CG5706 |
| AM11630 | GB19075 | XM_001120140.1 | FBgn0038198 | CG3153 |
| AM11637 | GB19082 | XM_393965.3 | FBgn0003517 | sta |
| AM11638 | GB19083 | XM_395625.3 | FBgn0031905 | CG5155 |
| AM11656 | GB19102 | XM_394508.3 | FBgn0037120 | CG11247 |
| AM11658 | GB19104 |  | FBgn0030715 | Or13a |
| AM11705 | GB19151 | XM_393358.3 | FBgn0035049 | Mmp1 |
| AM11745 | GB19191 | XM_624159.1 | FBgn0025391 | Scgdelta |
| AM11759 | GB19205 | XM_393767.3 | FBgn0037708 | CG9386 |
| AM11791 | GB19237 | XR_014971.1 | FBgn0025704 | CG2165 |
| AM11792 | GB19238 | XM_001121957.1 | FBgn0038149 | CG9796 |
| AM11812 | GB19258 | XM_001120053.1 | FBgn0042085 | Bap170 |
| AM11838 | GB19284 | XM_001120336.1 | FBgn0005639 | mxc |
| AM11865 | GB19312 | XM_001120612.1 | FBgn0037299 | CG1115 |
| AM11881 |  |  |  |  |
| AM11885 |  |  |  |  |
| AM11901 | GB19348 |  | FBgn0020503 | CLIP-190 |
| AM11902 | GB19349 | XM_001122684.1 | FBgn0041723 | rho-5 |
| AM11926 | GB19373 | XM_395694.3 | FBgn0037580 | DppIII |
| AM11934 | GB19381 | XM_392998.3 | FBgn0033813 | CG12765 |
| AM11940 | GB19387 | XM_623400.2 | FBgn0001186 | Hex-A |
| AM11945 | GB30228 | NM_001011616.1 | FBgn0010385 | Def |
| AM11951 | GB19398 | XM_624245.2 | FBgn0034129 | CG15925 |
| AM11970 | GB19418 | NM_001011574.1 | FBgn0001112 | Gld |
| AM11972 | GB19420 | XM_393429.2 | FBgn0002938 | ninaC |
| AM11977 | GB19425 | XM_623763.2 | FBgn0024352 | Hop |
| AM12008 | GB19457 |  | FBgn0000575 | emc |
| AM12011 | GB19460 | XM_623339.2 | FBgn0000064 | Ald |
| AM12012 | GB19460 | XM_623339.2 | FBgn0000064 | Ald |
| AM12020 |  |  |  |  |
| AM12033 | GB19481 | XR_014890.1 | FBgn0017566 | ND75 |
| AM12037 | GB19485 | XM_396554.2 | FBgn0052486 | CG32486 |
| AM12039 |  |  |  |  |
| AM12043 | GB19491 | XR_014891.1 | FBgn0016081 | fry |
| AM12055 | GB19503 | XM_623127.2 | FBgn0001219 | Hsc70-4 |
| AM12071 | GB19519 | XM_001122612.1 | FBgn0034978 | CG3257 |
| AM12078 | GB19526 | XM_393201.3 | FBgn0040390 | CG14047 |
| AM12083 | GB19531 | XM_392515.3 | FBgn0037357 | sec23 |
| AM12095 |  |  |  |  |
| AM12097 |  |  |  |  |
| AM12100 | GB19548 | XM_001121152.1 | FBgn0001311 | kkv |
| AM12149 | GB19596 | XM_392541.3 | FBgn0083982 | brp |
| AM12155 | GB19603 | XM_001122650.1 | FBgn0038912 | CG6656 |
| AM12156 | GB19604 | XM_392765.3 | FBgn0000568 | Eip75B |
| AM12159 | GB19607 | XM_396056.3 | FBgn0025800 | Smox |
| AM12205 | GB19656 | XM_393110.3 | FBgn0023528 | CG2924 |
| AM12207 | GB19658 | XM_001122296.1 | FBgn0031708 | CG7382 |
| AM12218 | GB19670 | XM_392390.2 | FBgn0038516 | CG5840 |
| AM12231 | GB19683-RB | XM_392752.3 | FBgn0010435 | emp |
| AM12251 |  |  |  |  |
| AM12256 | GB19709 | XM_392664.3 | FBgn0039900 | Syt7 |
| AM12288 | GB19741 | XM_001119855.1 | FBgn0034858 | eIF2B-delta |
| AM12297 |  |  |  |  |
| AM12312 | GB19765 | XM_624269.1 | FBgn0029846 | Ca-alpha1T |
| AM12318 | GB19771 | XM_395580.2 | FBgn0033738 | CG8830 |
| AM12340 | GB19793 | XM_393495.3 | FBgn0016031 | lama |
| AM12344 | GB19797 | XM_001119981.1 | FBgn0000473 | Cyp6a2 |
| AM12353 | GB19806 | XM_393362.3 | FBgn0050118 | CG30118 |
| AM12358 | GB19811 | NM_001011640.1 | FBgn0039203 | CG13618 |
| AM12382 | GB19835 | XM_624607.1 | FBgn0032612 | CG13282 |
| AM12390 | GB19843 | XM_624145.2 | FBgn0036768 | CG7402 |
| AM12393 | GB19846 | XM_394370.1 | FBgn0038485 | CG5255 |
| AM12400 | GB19853 | XM_393827.3 | FBgn0013753 | Bgb |
| AM12401 | GB19854 | XM_395316.2 | FBgn0030966 | CG7280 |
| AM12403 | GB19856 | XM_392730.3 | FBgn0051265 | CG31265 |
| AM12405 | GB19858 | XM_001120751.1 | FBgn0039584 | beat-VI |
| AM12422 | GB19875 | XM_001122114.1 | FBgn0034364 | CG5493 |
| AM12457 | GB19910 | XM_001122246.1 | FBgn0052158 | CG32158 |
| AM12492 | GB19945 | XM_001120543.1 | FBgn0010452 | trn |
| AM12518 | GB19971 |  | FBgn0004618 | gl |
| AM12553 | GB20006 | XM_001122124.1 | FBgn0041161 | blue |
| AM12558 | GB20011 | XM_001122854.1 | FBgn0035715 | CG10103 |
| AM12562 | GB20015 | XM_001122447.1 | FBgn0033869 | CG6305 |
| AM12583 | GB20036 | XM_392154.3 | FBgn0004242 | syt |
| AM12597 | GB20050 | XM_393179.3 | FBgn0039163 | CG5515 |
| AM12608 | GB20060 |  | FBgn0039749 | CG11498 |
| AM12614 | GB30368 | XM_001122810.1 | FBgn0037954 | glo |
| AM12616 | GB20068 | XM_624923.2 | FBgn0032846 | CG10721 |
| AM12617 | GB20069 | XM_001121334.1 | FBgn0027617 | CG5808 |
| AM12637 | GB20089 | XM_393378.3 | FBgn0052654 | CG32654 |
| AM12656 | GB20109 | XM_395903.3 | FBgn0039623 | CG1951 |
| AM12672 | GB20125 | XM_623695.2 | FBgn0037652 | CG11980 |
| AM12691 | GB20144 | XM_393278.3 | FBgn0039008 | CG6972 |
| AM12699 |  |  |  |  |
| AM12723 |  |  |  |  |
| AM12743 |  |  |  |  |
| AM12746 | GB30328 |  | FBgn0030291 | CG1738 |
| AM12756 | GB30320 |  | FBgn0045495 | Gr28b |
| AM12765 |  |  |  |  |
| AM12768 | GB30101 | XM_394261.3 | FBgn0025741 | plexA |
| AM12781 | GB30362 | XR_014951.1 | FBgn0002565 | Lsp2 |
| AM12801 | GB14865 | NM_001011592.1 | FBgn0029508 | Tsp42Ea |
| AM12811 | GB16999 | XM_624012.1 | FBgn0039354 | Lgr3 |
| AM12813 |  |  |  |  |
| AM12840 |  |  |  |  |
| AM12843R |  |  |  |  |
| AM12844 |  |  |  |  |
| AM12844R |  |  |  |  |
| AM12845 |  |  |  |  |
| AM12845R |  |  |  |  |
| AM12850R |  |  |  |  |
| AM12851R | GB30268 | XM_624659.2 | FBgn0010226 | GstS1 |
| AM12852R |  |  |  |  |
| AM12864 |  |  |  |  |
| AM12864R |  |  |  |  |
| AM12877R |  |  |  |  |
| AM12899 |  |  |  |  |

**S2. 960 Significantly-regulated transcripts for retinue response.** There were 960 genes that were significantly associated with retinue response at FDR<0.01. The first column contains the transcript identifier associated with the microarray, and the subsequent columns are the corresponding honey bee predicted gene names (GB names) and fly orthologs (the flybase identifiers) if available.
