# Supplementary material for: Individual Variation in Pheromone Response Correlates with Reproductive Traits and Brain Gene Expression in Worker Honey Bees
Source: PLoS One. 2010 Feb 9;5(2):e9116. doi: 10.1371/journal.pone.0009116 (PMC2817734; doi:10.1371/journal.pone.0009116)
Supplement: Table S3 — Transcripts consistently regulated across both colonies. 360 transcripts were consistently up- or down-regulated in high responding individuals in both colonies. (0.63 MB DOC) [file pone.0009116.s004.doc]

|  | **AM** | **GB** | **XM** | **FlyBaseID** | **Gene Name** |
| --- | --- | --- | --- | --- | --- |
| **High>low** | AM12746 | GB30328 |  | FBgn0030291 | CG1738 |
|  | AM12691 | GB20144 | XM_393278.3 | FBgn0039008 | CG6972 |
|  | AM12637 | GB20089 | XM_393378.3 | FBgn0052654 | CG32654 |
|  | AM12617 | GB20069 | XM_001121334.1 | FBgn0027617 | CG5808 |
|  | AM12616 | GB20068 | XM_624923.2 | FBgn0032846 | CG10721 |
|  | AM12614 | GB30368 | XM_001122810.1 | FBgn0037954 | glo |
|  | AM12608 | GB20060 |  | FBgn0039749 | CG11498 |
|  | AM12558 | GB20011 | XM_001122854.1 | FBgn0035715 | CG10103 |
|  | AM12492 | GB19945 | XM_001120543.1 | FBgn0010452 | trn |
|  | AM12457 | GB19910 | XM_001122246.1 | FBgn0052158 | CG32158 |
|  | AM12405 | GB19858 | XM_001120751.1 | FBgn0039584 | beat-VI |
|  | AM12403 | GB19856 | XM_392730.3 | FBgn0051265 | CG31265 |
|  | AM12401 | GB19854 | XM_395316.2 | FBgn0030966 | CG7280 |
|  | AM12390 | GB19843 | XM_624145.2 | FBgn0036768 | CG7402 |
|  | AM12358 | GB19811 | NM_001011640.1 | FBgn0039203 | CG13618 |
|  | AM12353 | GB19806 | XM_393362.3 | FBgn0050118 | CG30118 |
|  | AM12318 | GB19771 | XM_395580.2 | FBgn0033738 | CG8830 |
|  | AM12256 | GB19709 | XM_392664.3 | FBgn0039900 | Syt7 |
|  | AM12207 | GB19658 | XM_001122296.1 | FBgn0031708 | CG7382 |
|  | AM12205 | GB19656 | XM_393110.3 | FBgn0023528 | CG2924 |
|  | AM12083 | GB19531 | XM_392515.3 | FBgn0037357 | sec23 |
|  | AM12078 | GB19526 | XM_393201.3 | FBgn0040390 | CG14047 |
|  | AM12033 | GB19481 | XR_014890.1 | FBgn0017566 | ND75 |
|  | AM12008 | GB19457 |  | FBgn0000575 | emc |
|  | AM11970 | GB19418 | NM_001011574.1 | FBgn0001112 | Gld |
|  | AM11951 | GB19398 | XM_624245.2 | FBgn0034129 | CG15925 |
|  | AM11926 | GB19373 | XM_395694.3 | FBgn0037580 | DppIII |
|  | AM11901 | GB19348 |  | FBgn0020503 | CLIP-190 |
|  | AM11792 | GB19238 | XM_001121957.1 | FBgn0038149 | CG9796 |
|  | AM11759 | GB19205 | XM_393767.3 | FBgn0037708 | CG9386 |
|  | AM11656 | GB19102 | XM_394508.3 | FBgn0037120 | CG11247 |
|  | AM11630 | GB19075 | XM_001120140.1 | FBgn0038198 | CG3153 |
|  | AM11608 | GB19053 | XM_623498.2 | FBgn0039175 | CG5706 |
|  | AM11541 | GB18985 | XM_001121704.1 | FBgn0027509 | CG7261 |
|  | AM11498 | GB18942 |  | FBgn0053513 | Nmdar2 |
|  | AM11475 | GB18918 |  | FBgn0004606 | zfh1 |
|  | AM11450 | GB30325 | XM_001120490.1 | FBgn0039338 | XNP |
|  | AM11418 | GB18861 | XM_395100.2 | FBgn0036376 | CG10743 |
|  | AM11416 | GB18859 | XM_395547.3 | FBgn0034049 | CG8291 |
|  | AM11412 | GB30326 | XM_001120439.1 | FBgn0035445 | CG12014 |
|  | AM11364 | GB18806 | XM_001120186.1 | FBgn0053862 | His2A:CG33862 |
|  | AM11325 | GB18765 | XM_394739.2 | FBgn0032217 | CG4972 |
|  | AM11318 | GB18758 | XM_001121502.1 | FBgn0026433 | Grip128 |
|  | AM11260 | GB18699 | XM_392656.3 | FBgn0033657 | CG8271 |
|  | AM11203 | GB18642 | XM_625293.2 | FBgn0015390 | futsch |
|  | AM11033 | GB18471 | XM_001120276.1 | FBgn0032223 | GATAd |
|  | AM10982 | GB18419 | XM_397407.2 | FBgn0038501 | CG5319 |
|  | AM10977 | GB18414 | XM_393751.3 | FBgn0000024 | Ace |
|  | AM10939 | GB18375 | XM_001120538.1 | FBgn0028992 | sds22 |
|  | AM10931 | GB18367 |  | FBgn0037465 | CG1105 |
|  | AM10808 | GB18242 | XM_623115.2 | FBgn0001205 | Hmgcr |
|  | AM10555 | GB17982 | XM_392419.3 | FBgn0052372 | CG32372 |
|  | AM10510 | GB17937 | XM_396062.3 | FBgn0030320 | CG2247 |
|  | AM10504 | GB17931 | XM_623663.2 | FBgn0034145 | CG5065 |
|  | AM10471 | GB17900 | XM_395852.3 | FBgn0036169 | CG6128 |
|  | AM10438 | GB17867 | XM_396828.3 | FBgn0052698 | CG32698 |
|  | AM10436 | GB17865 | XM_393204.3 | FBgn0039505 | CG5934 |
|  | AM10433 | GB17862 | XM_624898.2 | FBgn0030327 | FucT6 |
|  | AM10373 | GB17801 | XM_397517.3 | FBgn0037734 | CG9448 |
|  | AM10351 | GB17778 | XM_392081.3 | FBgn0033160 | CG11107 |
|  | AM10313 | GB17740 | XM_394711.3 | FBgn0037622 | CG8202 |
|  | AM10281 | GB17708 | XM_001122173.1 | FBgn0031575 | CG3980 |
|  | AM10238 | GB17664 | XM_392261.3 | FBgn0027600 | CG4778 |
|  | AM10193 | GB17619 | XM_624722.2 | FBgn0028969 | deltaCOP |
|  | AM10162 | GB17591 | XM_624946.2 | FBgn0003277 | RpII215 |
|  | AM10161 | GB17590 | XM_623846.2 | FBgn0028863 | CG4587 |
|  | AM10148 | GB17576 |  | FBgn0053017 | CG33017 |
|  | AM10137 | GB17565 |  | FBgn0051064 | CG31064 |
|  | AM10123 | GB17552 | XM_623714.2 | FBgn0028434 | Ercc1 |
|  | AM10107 | GB17536 |  | FBgn0039215 | CG6695 |
|  | AM10041 | GB17470 | XM_625190.1 | FBgn0026582 | CG9418 |
|  | AM09981 | GB17409 |  | FBgn0036945 | CG6981 |
|  | AM09953 | GB17380 | XM_393141.3 | FBgn0014163 | fax |
|  | AM09918 | GB17343 | XM_391845.3 | FBgn0020245 | ttv |
|  | AM09888 | GB17313 |  | FBgn0030109 | CG12121 |
|  | AM09799 | GB17224 | XM_391881.3 | FBgn0035888 | CG7120 |
|  | AM09784 | GB17209 | XM_397270.3 | FBgn0040475 | SH3PX1 |
|  | AM09755 | GB17180 | XM_001123067.1 | FBgn0032457 | CG15483 |
|  | AM09724 | GB17149 | XM_001121890.1 | FBgn0034083 | lbk |
|  | AM09708 | GB17133 | XM_001121970.1 | FBgn0000036 | nAcRalpha-96Aa |
|  | AM09692 | GB17117 | XM_001120160.1 | FBgn0042713 | Gpi1 |
|  | AM09674 | GB17099 | XM_395912.3 | FBgn0001978 | stc |
|  | AM09669 | GB17094 |  | FBgn0029749 | CG15786 |
|  | AM09663 | GB17088 | XM_396435.3 | FBgn0030692 | mRpS30 |
|  | AM09642 | GB17066 | XM_392396.3 | FBgn0035039 | CG3608 |
|  | AM09636 | GB17060 | XM_392005.3 | FBgn0030364 | CG15735 |
|  | AM09557 | GB16980 | XM_395330.2 | FBgn0031360 | CG31937 |
|  | AM09441 | GB16864 | XM_393806.3 | FBgn0028479 | CG4389 |
|  | AM09275 | GB16696 |  | FBgn0038531 | CG14325 |
|  | AM09241 | GB16663 | XM_394258.3 | FBgn0036309 | CG10971 |
|  | AM09211 | GB16633 | XM_392959.3 | FBgn0002989 | okr |
|  | AM09209 | GB30075 |  | FBgn0039728 | CG7896 |
|  | AM09197 | GB16619 | XM_393860.3 | FBgn0027570 | Nep2 |
|  | AM09082 | GB16503 | XM_394173.3 | FBgn0051619 | CG31619 |
|  | AM09028 | GB16449 | XM_001120474.1 | FBgn0037339 | Pi4KIIalpha |
|  | AM09002 | GB16423 | XM_393927.3 | FBgn0000986 | Fs(2)Ket |
|  | AM08990 | GB16409 | XM_001122322.1 | FBgn0031537 | sec5 |
|  | AM08987 | GB16406 | XM_001122973.1 | FBgn0030854 | CG8289 |
|  | AM08952 | GB16371 | XM_623438.1 | FBgn0033570 | CG7712 |
|  | AM08895 | GB16315 | XM_394997.3 | FBgn0037202 | Ssl1 |
|  | AM08874 | GB16294 | XM_624795.2 | FBgn0034603 | Glycogenin |
|  | AM08796 | GB16216 | XM_395372.3 | FBgn0035833 | CG7565 |
|  | AM08740 | GB16162 | XM_394745.3 | FBgn0031992 | CG8498 |
|  | AM08641 | GB16060 | XM_394552.3 | FBgn0037736 | CG12950 |
|  | AM08625 | GB16044 | XM_001120488.1 | FBgn0037142 | CG14562 |
|  | AM08572 | GB15990 | NM_001011636.1 | FBgn0037913 | CG6783 |
|  | AM08562 | GB15981 |  | FBgn0023518 | trr |
|  | AM08490 | GB15909 | XM_395295.3 | FBgn0005427 | ewg |
|  | AM08483 | GB15902 | XM_624680.2 | FBgn0025186 | ari-2 |
|  | AM08345 | GB15763 | XM_001122793.1 | FBgn0032042 | CG13398 |
|  | AM08306 | GB15723 | XM_624240.2 | FBgn0030789 | Rrp45 |
|  | AM08260 | GB15677 | XM_392392.3 | FBgn0039776 | PH4alphaEFB |
|  | AM08140 | GB15555 | XM_001120138.1 | FBgn0034223 | CG6522 |
|  | AM08041 | GB15454 |  | FBgn0035497 | CG14995 |
|  | AM07979 | GB15391 | XM_001121069.1 | FBgn0030839 | CG5613 |
|  | AM07975 | GB15387 |  | FBgn0001280 | janA |
|  | AM07969 | GB15381 | XM_001122064.1 | FBgn0052677 | CG32677 |
|  | AM07921 | GB15333 | XM_624887.2 | FBgn0042094 | Adk3 |
|  | AM07894 | GB15304 | XM_396744.3 | FBgn0033886 | CG13349 |
|  | AM07868 | GB15279 | XM_001120221.1 | FBgn0030940 | CG15040 |
|  | AM07846 | GB15257 | XM_625193.2 | FBgn0039003 | wfs1 |
|  | AM07790 | GB15201 | XM_001120484.1 | FBgn0039296 | CG10420 |
|  | AM07609 | GB15018 | XM_001120243.1 | FBgn0029167 | Hml |
|  | AM07607 | GB15016 | XM_393090.3 | FBgn0001218 | Hsc70-3 |
|  | AM07579 | GB14988 | XM_623305.2 | FBgn0051712 | CG31712 |
|  | AM07563 | GB14971 |  | FBgn0051072 | Lerp |
|  | AM07549 | GB14958 | XR_015059.1 | FBgn0020615 | SelD |
|  | AM07546 | GB14955 | XM_623701.2 | FBgn0028658 | adat |
|  | AM07512 | GB14921 |  | FBgn0025639 | Suv4-20 |
|  | AM07488 | GB14894 | XM_395088.3 | FBgn0053002 | mRpL27 |
|  | AM07474 | GB14880 | XR_014875.1 | FBgn0040002 | CG17683 |
|  | AM07411 | GB14816 | XM_001122257.1 | FBgn0034096 | CG7786 |
|  | AM07405 | GB14810 | XM_001120586.1 | FBgn0022787 | Hel89B |
|  | AM07389 | GB14794 | XM_396616.2 | FBgn0028992 | sds22 |
|  | AM07360 | GB14765 | XM_396662.1 | FBgn0026147 | CG16833 |
|  | AM07299 | GB14702 | XM_001119932.1 | FBgn0038588 | CG7156 |
|  | AM07292 | GB14695 | XM_397223.3 | FBgn0030230 | Rph |
|  | AM07274 | GB14677 | XM_395924.2 | FBgn0039241 | CG11089 |
|  | AM07205 | GB14608 | XM_623717.2 | FBgn0032221 | CG5375 |
|  | AM07169 | GB14572 | XM_392549.3 | FBgn0030234 | CG15211 |
|  | AM07154 | GB14556 | XM_623782.2 | FBgn0035364 | CG14950 |
|  | AM06980 | GB14382 | XM_624192.2 | FBgn0013997 | Nrx-IV |
|  | AM06954 | GB14356 | XM_393087.3 | FBgn0034372 | Gint3 |
|  | AM06941 | GB14342 | XM_624197.2 | FBgn0040337 | CG3021 |
|  | AM06852 | GB14253 | XM_394497.3 | FBgn0036500 | CG7275 |
|  | AM06807 | GB14208 | XM_624177.2 | FBgn0052627 | CG32627 |
|  | AM06762 | GB14162 | XM_624216.2 | FBgn0030788 | CG4756 |
|  | AM06718 | GB14118 | XM_393542.3 | FBgn0024555 | flfl |
|  | AM06676 | GB14074 | XM_001121228.1 | FBgn0037656 | CG11986 |
|  | AM06645 | GB30234 | XM_624189.2 | FBgn0005658 | Ets65A |
|  | AM06516 | GB13918 | XM_624993.2 | FBgn0032285 | CG17108 |
|  | AM06436 | GB13837 | XM_623602.1 | FBgn0035026 | CG12252 |
|  | AM06391 | GB13792 | XM_624751.2 | FBgn0004797 | mdy |
|  | AM06389 | GB13790 |  | FBgn0028872 | CG18095 |
|  | AM06370 | GB13771 | XM_623889.2 | FBgn0050296 | CG30296 |
|  | AM06342 | GB13743 | XM_001121853.1 | FBgn0032397 | Tom70 |
|  | AM06300 | GB13702 | XM_394417.3 | FBgn0034735 | CG4610 |
|  | AM06267 | GB13669 |  | FBgn0030940 | CG15040 |
|  | AM06224 | GB13625 |  | FBgn0037238 | CG1090 |
|  | AM06178 | GB13577 | XM_392075.3 | FBgn0026086 | Adar |
|  | AM06135 | GB13534 | XM_001121402.1 | FBgn0031300 | CG4644 |
|  | AM06117 | GB13515 | XM_394615.3 | FBgn0038745 | CG4538 |
|  | AM06099 | GB13497 | XM_394801.3 | FBgn0026620 | tacc |
|  | AM06028 | GB13424 | XM_397443.3 | FBgn0011704 | RnrS |
|  | AM05999 | GB13396 |  | FBgn0030576 | CG15890 |
|  | AM05998 | GB13395 | XM_392802.3 | FBgn0028475 | CG10221 |
|  | AM05929 | GB13325 | XM_001120200.1 | FBgn0011695 | PebIII |
|  | AM05926 | GB13322 | XM_396686.2 | FBgn0034057 | CG8314 |
|  | AM05897 | GB13290 | XR_015057.1 | FBgn0039257 | tnc |
|  | AM05893 | GB13286 | XM_624563.2 | FBgn0039115 | CG10214 |
|  | AM05827 | GB13219 | XM_001120341.1 | FBgn0041607 | asparagine-synthetase |
|  | AM05764 | GB13155 | XM_396831.2 | FBgn0030884 | CG6847 |
|  | AM05726 | GB13118 | XM_001120264.1 | FBgn0010278 | Ssrp |
|  | AM05615 | GB13004 | XM_392381.3 | FBgn0037303 | CG12163 |
|  | AM05614 | GB13003 | XM_393066.3 | FBgn0005648 | Pabp2 |
|  | AM05613 | GB13002 | XM_396099.2 | FBgn0029853 | CG3781 |
|  | AM05602 | GB12991 | XM_624164.2 | FBgn0001197 | His2Av |
|  | AM05570 | GB12957 | XM_392116.3 | FBgn0038492 | CG4090 |
|  | AM05524 | GB12911 | XM_001121342.1 | FBgn0040334 | Tsp3A |
|  | AM05460 | GB12846 |  | FBgn0052048 | CG32048 |
|  | AM05454 | GB12840 | XM_001121454.1 | FBgn0003498 | sqd |
|  | AM05446 | GB12832 |  | FBgn0035254 | CG7974 |
|  | AM05394 | GB12779 | XM_394467.1 | FBgn0027936 | vih |
|  | AM05361 | GB12746 | XM_392803.3 | FBgn0052133 | CG32133 |
|  | AM05338 | GB12723 | XM_396382.2 | FBgn0023527 | CG3071 |
|  | AM05319 | GB12704 | XM_001121319.1 | FBgn0037536 | CG2698 |
|  | AM05202 | GB12586 | XM_623828.2 | FBgn0014002 | Pdi |
|  | AM05177 | GB12561 | XM_001123021.1 | FBgn0033714 | garz |
|  | AM05153 | GB12537 | XM_001122033.1 | FBgn0038540 | CG14321 |
|  | AM05145 | GB12529 | NM_001040263.1 | FBgn0031148 | CG1753 |
|  | AM05132 | GB12515 | XM_392635.3 | FBgn0038055 | trus |
|  | AM05089 | GB12472 | XM_001122127.1 | FBgn0020309 | crol |
|  | AM04987 | GB12369 |  | FBgn0038282 | dpr9 |
|  | AM04979 | GB12361 | XM_001119826.1 | FBgn0032518 | RpL24 |
|  | AM04978 | GB12360 | XM_393904.3 | FBgn0017558 | Pdk |
|  | AM04964 | GB12346 | XM_392132.3 | FBgn0031816 | CG16947 |
|  | AM04954 | GB12335 | XM_393488.3 | FBgn0032197 | CG5694 |
|  | AM04953 | GB12334 | XR_015063.1 | FBgn0051064 | CG31064 |
|  | AM04926 | GB12307 | XM_392283.3 | FBgn0035953 | CG5087 |
|  | AM04880 | GB30566 |  | FBgn0037836 | CG14692 |
|  | AM04843 | GB12224 | XM_395190.3 | FBgn0024983 | CG4293 |
|  | AM04837 | GB12218 | XM_001122184.1 | FBgn0053864 | His1:CG33864 |
|  | AM04676 | GB12059 | XM_001122049.1 | FBgn0031781 | Arc-p20 |
|  | AM04620 | GB12003 | XM_001122998.1 | FBgn0037794 | CG6254 |
|  | AM04599 | GB11982 | XM_001120920.1 | FBgn0032811 | CG10268 |
|  | AM04584 | GB11967 | XM_001121588.1 | FBgn0034271 | CG4996 |
|  | AM04550 | GB11933 | XM_392880.3 | FBgn0032597 | CG17904 |
|  | AM04541 | GB11924 |  | FBgn0002521 | pho |
|  | AM04500 | GB11882 | XM_393277.2 | FBgn0029937 | CG8300 |
|  | AM04487 | GB11868 | XM_392276.3 | FBgn0037072 | Rab26 |
|  | AM04470 | GB11851 | XM_001122102.1 | FBgn0032296 | CG6729 |
|  | AM04467 | GB11848 | XM_623072.2 | FBgn0029003 | mab-2 |
|  | AM04460 | GB11841 | XM_001123352.1 | FBgn0026438 | Eaat2 |
|  | AM04439 | GB11820 | XM_393343.3 | FBgn0016672 | Ipp |
|  | AM04436 | GB11817 | XM_001120108.1 | FBgn0020762 | Atet |
|  | AM04427 | GB11808 | XM_393986.3 | FBgn0036240 | CG6928 |
|  | AM04269 | GB11649 | XM_392475.3 | FBgn0053080 | CG33080 |
|  | AM04245 | GB11625 | XM_394067.2 | FBgn0028679 | Sema-5c |
|  | AM04229 | GB11609 | XM_394021.1 | FBgn0039352 | CG5053 |
|  | AM04196 | GB11576 | XM_624016.2 | FBgn0031977 | CG7380 |
|  | AM04187 | GB11567 | XM_623735.2 | FBgn0024754 | Flo |
|  | AM04117 | GB11497 | XM_397449.2 | FBgn0035895 | Unr |
|  | AM04102 | GB11481 |  | FBgn0027499 | CG12340 |
|  | AM04073 | GB11452 | XM_396755.3 | FBgn0053208 | MICAL |
|  | AM04072 | GB11451 | XM_624637.2 | FBgn0032731 | CG10641 |
|  | AM04066 | GB11445 | XM_396120.2 | FBgn0033252 | CG12769 |
|  | AM04025 | GB11404 | XM_001120829.1 | FBgn0003744 | trc |
|  | AM04023 | GB11402 |  | FBgn0024273 | WASp |
|  | AM03980 | GB11359 | XM_001120625.1 | FBgn0027083 | Aats-met |
|  | AM03911 | GB11288 | XR_014873.1 | FBgn0004242 | syt |
|  | AM03884 | GB11261 | XM_393332.3 | FBgn0039348 | CG4673 |
|  | AM03854 | GB11231 | XM_394846.2 | FBgn0052495 | CG32495 |
|  | AM03795 | GB11171 | XM_623605.2 | FBgn0020503 | CLIP-190 |
|  | AM03765 | GB11141 |  | FBgn0005654 | lat |
|  | AM03731 | GB11107 | XM_393409.3 | FBgn0027568 | CG5366 |
|  | AM03644 | GB11020 | XM_395466.2 | FBgn0036031 | CG6761 |
|  | AM03628 | GB30096 | XM_624473.2 | FBgn0010762 | simj |
|  | AM03623 | GB10999 | XM_392117.3 | FBgn0034537 | DMAP1 |
|  | AM03618 | GB10995 | XM_001121420.1 | FBgn0053205 | CG33205 |
|  | AM03560 | GB10935 | XM_393997.1 | FBgn0030346 | CG11802 |
|  | AM03530 | GB10905 | XM_397272.3 | FBgn0015032 | Cyp4c3 |
|  | AM03512 | GB10887 | XM_001121440.1 | FBgn0045035 | tefu |
|  | AM03506 | GB10881 | XM_001121324.1 | FBgn0031498 | CG17260 |
|  | AM03434 | GB10809 | XM_395880.3 | FBgn0036446 | CG9384 |
|  | AM03421 | GB10796 | XM_625214.1 | FBgn0028693 | Rpn12 |
|  | AM03420 | GB10795 | XM_392277.3 | FBgn0033717 | CG8839 |
|  | AM03370 | GB10745 | XR_015068.1 | FBgn0052103 | CG32103 |
|  | AM03295 | GB10669 | XM_001122174.1 | FBgn0034743 | RpS16 |
|  | AM03284 | GB10657 | XM_392432.3 | FBgn0015808 | ScpX |
|  | AM03283 | GB10656 |  | FBgn0020309 | crol |
|  | AM03251 | GB10625 | XM_392434.3 | FBgn0034389 | Mctp |
|  | AM03236 | GB10608 | XM_393376.3 | FBgn0017572 | Mo25 |
|  | AM03234 | GB10607 | XM_624381.2 | FBgn0028421 | Kap3 |
|  | AM03181 | GB10554 | XM_397338.2 | FBgn0031255 | BBS8 |
|  | AM03126 | GB10500 | XR_014888.1 | FBgn0021874 | Nle |
|  | AM03092 | GB10465 |  | FBgn0036141 | srt |
|  | AM03024 | GB10396 | XM_393222.3 | FBgn0030141 | Gga |
|  | AM03021 | GB10393 | XM_001122723.1 | FBgn0032793 | CG10189 |
|  | AM03005 | GB10376 | XM_001120777.1 | FBgn0036741 | CG7510 |
|  | AM02954 | GB10324 |  | FBgn0052529 | CG32529 |
|  | AM02936 | GB10306 | XM_395966.2 | FBgn0032934 | CG8679 |
|  | AM02910 | GB10280 | XM_393616.3 | FBgn0024728 | Slip1 |
|  | AM02897 | GB30104 | XM_001122389.1 | FBgn0035917 | CG6416 |
|  | AM02863 | GB10236 | XM_001122939.1 | FBgn0022786 | Hira |
|  | AM02834 | GB10207 | XM_392642.2 | FBgn0037674 | Vps16A |
|  | AM02814 | GB10187 |  | FBgn0003175 | px |
|  | AM02746 | GB10118 | XM_391857.2 | FBgn0040297 | Nhe2 |
|  | AM02720 | GB10092 | XM_395179.2 | FBgn0027321 | l(1)G0060 |
|  | AM02706 | GB10078 | XM_396244.1 | FBgn0033046 | CG14470 |
|  | AM02669 | GB10041 | XM_001119847.1 | FBgn0031990 | CG8552 |
|  | AM02626 | GB19418 | NM_001011574.1 | FBgn0001112 | Gld |
|  | AM00070 |  |  |  |  |
|  | AM00109 |  |  |  |  |
|  | AM00417R |  |  |  |  |
|  | AM00429R |  |  |  |  |
|  | AM00539R |  |  |  |  |
|  | AM00597 |  |  |  |  |
|  | AM00598 |  |  |  |  |
|  | AM00626R |  |  |  |  |
|  | AM00688 |  |  |  |  |
|  | AM00717R |  |  |  |  |
|  | AM00737 |  |  |  |  |
|  | AM00749 |  |  |  |  |
|  | AM00787 |  |  |  |  |
|  | AM00830 |  |  |  |  |
|  | AM00859 |  |  |  |  |
|  | AM00894 |  |  |  |  |
|  | AM00904 |  |  |  |  |
|  | AM00951 |  |  |  |  |
|  | AM00954 |  |  |  |  |
|  | AM01004 |  |  |  |  |
|  | AM01033 |  |  |  |  |
|  | AM01037 |  |  |  |  |
|  | AM01108 |  |  |  |  |
|  | AM01226 |  |  |  |  |
|  | AM01232 |  |  |  |  |
|  | AM01324 |  |  |  |  |
|  | AM01391 |  |  |  |  |
|  | AM01416 |  |  |  |  |
|  | AM01447 |  |  |  |  |
|  | AM01474 |  |  |  |  |
|  | AM01515 |  |  |  |  |
|  | AM01524 |  |  |  |  |
|  | AM01529 |  |  |  |  |
|  | AM01607 |  |  |  |  |
|  | AM01722 |  |  |  |  |
|  | AM01737 |  |  |  |  |
|  | AM01763 |  |  |  |  |
|  | AM01838 |  |  |  |  |
|  | AM01880 |  |  |  |  |
|  | AM01901 |  |  |  |  |
|  | AM01911 |  |  |  |  |
|  | AM01921 |  |  |  |  |
|  | AM01977 |  |  |  |  |
|  | AM01986 |  |  |  |  |
|  | AM01994 |  |  |  |  |
|  | AM02006 |  |  |  |  |
|  | AM02118 |  |  |  |  |
|  | AM02150 |  |  |  |  |
|  | AM02154 |  |  |  |  |
|  | AM02158 |  |  |  |  |
|  | AM02160 |  |  |  |  |
|  | AM02247 |  |  |  |  |
|  | AM02281 |  |  |  |  |
|  | AM02333 |  |  |  |  |
|  | AM02367 |  |  |  |  |
|  | AM02418 |  |  |  |  |
|  | AM02442 |  |  |  |  |
|  | AM02488 |  |  |  |  |
|  | AM02617 |  |  |  |  |
|  | AM03211 |  |  |  |  |
|  | AM03347 |  |  |  |  |
|  | AM03972 |  |  |  |  |
|  | AM05067 |  |  |  |  |
|  | AM05960 |  |  |  |  |
|  | AM06589 |  |  |  |  |
|  | AM06712 |  |  |  |  |
|  | AM07565 |  |  |  |  |
|  | AM07863 |  |  |  |  |
|  | AM08466 |  |  |  |  |
|  | AM09529 |  |  |  |  |
|  | AM09580 |  |  |  |  |
|  | AM09801 |  |  |  |  |
|  | AM09945 |  |  |  |  |
|  | AM10092 |  |  |  |  |
|  | AM10160 |  |  |  |  |
|  | AM10179 |  |  |  |  |
|  | AM10638 |  |  |  |  |
|  | AM10778 |  |  |  |  |
|  | AM11030 |  |  |  |  |
|  | AM11125 |  |  |  |  |
|  | AM11156 |  |  |  |  |
|  | AM11565 |  |  |  |  |
|  | AM12020 |  |  |  |  |
|  | AM12095 |  |  |  |  |
|  | AM12097 |  |  |  |  |
|  | AM12297 |  |  |  |  |
|  | AM12699 |  |  |  |  |
|  | AM12723 |  |  |  |  |
|  | AM12813 |  |  |  |  |
| ***Low > High*** | AM00021 |  |  |  |  |
|  | AM00021R |  |  |  |  |
|  | AM00148 |  |  |  |  |
|  | AM00244 | GB15899 |  | FBgn0041621 | Or82a |
|  | AM00333 | GB16692 | XM_396566.3 | FBgn0039805 | CG12045 |
|  | AM00351 | GB13473 | NM_001011642.1 | FBgn0035544 | CG15021 |
|  | AM00352 | GB13473 | NM_001011613.1 | FBgn0035544 | CG15021 |
|  | AM00353 | GB13473 | XM_001123211.1 | FBgn0035544 | CG15021 |
|  | AM00354 | GB17782 | NM_001011642.1 | FBgn0035544 | CG15021 |
|  | AM00355 | GB17782 | NM_001011642.1 | FBgn0035544 | CG15021 |
|  | AM00356 | GB17782 | XM_001123211.1 | FBgn0035544 | CG15021 |
|  | AM00357 | GB17782 | NM_001011613.1 | FBgn0035544 | CG15021 |
|  | AM00358 | GB13473-RB | NM_001011613.1 | FBgn0035544 | CG15021 |
|  | AM00359 |  |  |  |  |
|  | AM00360 | GB17782 | NM_001011642.1 | FBgn0035544 | CG15021 |
|  | AM00413R |  |  |  |  |
|  | AM00429 |  |  |  |  |
|  | AM00459R |  |  |  |  |
|  | AM00463R |  |  |  |  |
|  | AM00523 |  |  |  |  |
|  | AM00683 |  |  |  |  |
|  | AM00763 |  |  |  |  |
|  | AM00826 |  |  |  |  |
|  | AM00969 |  |  |  |  |
|  | AM01053 |  |  |  |  |
|  | AM01138 | GB15745 |  | FBgn0036975 | CG5618 |
|  | AM01177 |  |  |  |  |
|  | AM01255 |  |  |  |  |
|  | AM01296 |  |  |  |  |
|  | AM01340 |  |  |  |  |
|  | AM01508 |  |  |  |  |
|  | AM01619 |  |  |  |  |
|  | AM01634 |  |  |  |  |
|  | AM01636 |  |  |  |  |
|  | AM01760 |  |  |  |  |
|  | AM01818 |  |  |  |  |
|  | AM01845 |  |  |  |  |
|  | AM01892 |  |  |  |  |
|  | AM02125 |  |  |  |  |
|  | AM02135 |  |  |  |  |
|  | AM02144 |  |  |  |  |
|  | AM02224 |  |  |  |  |
|  | AM02265 |  |  |  |  |
|  | AM02530 |  |  |  |  |
|  | AM02537 |  |  |  |  |
|  | AM02742 | GB10114 | XM_393807.3 | FBgn0043364 | cbt |
|  | AM02766 | GB10138 | XM_392309.2 | FBgn0016920 | nompC |
|  | AM02832 | GB10205 | XM_393414.3 | FBgn0002478 | l(3)IX-14 |
|  | AM02926 | GB10296 | XM_393619.2 | FBgn0038296 | CG6752 |
|  | AM02992 | GB10362 | XM_001120922.1 | FBgn0038402 | Fer2 |
|  | AM03091 | GB10464 |  | FBgn0039357 | CG4743 |
|  | AM03136 | GB10509 | XM_396657.3 | FBgn0027335 | l(1)G0003 |
|  | AM03624 |  |  |  |  |
|  | AM03646 | GB11022 | NM_001014429.1 | FBgn0039896 | yellow-h |
|  | AM03811 | GB11187 | XM_624300.1 | FBgn0025716 | Bap55 |
|  | AM03820 | GB11197 |  | FBgn0013813 | Dhc98D |
|  | AM03879 | GB11256 | XM_001122900.1 | FBgn0032029 | CG17292 |
|  | AM04060 |  |  |  |  |
|  | AM04186 | GB11566 | XM_623754.2 | FBgn0004102 | oc |
|  | AM04409 |  |  |  |  |
|  | AM04423 | GB11804 | XM_623873.2 | FBgn0027592 | MED15 |
|  | AM04434 | GB11815 | XM_393006.3 | FBgn0035147 | CG12030 |
|  | AM04456 |  |  |  |  |
|  | AM04469 | GB11850 | XM_393443.3 | FBgn0036335 | mRpL20 |
|  | AM04618 | GB12001 | XM_623485.2 | FBgn0051666 | CG31666 |
|  | AM04828 |  |  |  |  |
|  | AM04906 | GB12287 | XM_396705.2 | FBgn0000180 | bib |
|  | AM04975 | GB12357 | XM_394795.2 | FBgn0029157 | ssh |
|  | AM05112 | GB12495 | XM_625286.2 | FBgn0033603 | CG13214 |
|  | AM05189 | GB12573 | XM_393545.3 | FBgn0026708 | l(1)G0030 |
|  | AM05255 |  |  |  |  |
|  | AM05351 | GB12736 | XM_001122401.1 | FBgn0039385 | CG5913 |
|  | AM05395 | GB12780 | XM_396405.2 | FBgn0023521 | CG3587 |
|  | AM05426 | GB12811 | XM_393452.3 | FBgn0003065 | CG2150 |
|  | AM05467 | GB12853 | XM_392099.3 | FBgn0015609 | CadN |
|  | AM05534 | GB12922 | XM_001119846.1 | FBgn0061209 | His2B:CG17949 |
|  | AM05638 |  |  |  |  |
|  | AM05655 | GB13045 | XM_395766.3 | FBgn0039349 | CG4685 |
|  | AM05741 | GB13133 | XM_001120919.1 | FBgn0026314 | Ugt35b |
|  | AM05946 |  |  |  |  |
|  | AM06051 | GB13447 |  | FBgn0051224 | CG31224 |
|  | AM06194 | GB13594 | NM_001011651.1 | FBgn0013348 | TpnC41C |
|  | AM06255 | GB13657 | XM_393596.3 | FBgn0039214 | CG5794 |
|  | AM06315 | GB13717 | XM_001122365.1 | FBgn0034695 | CG13503 |
|  | AM06363 | GB13764 | XM_393750.3 | FBgn0034602 | CG15658 |
|  | AM06524 | GB13926 |  | FBgn0004569 | argos |
|  | AM06656 | GB14057 | XM_396289.2 | FBgn0031252 | CG13690 |
|  | AM06657 |  |  |  |  |
|  | AM06872 | GB14273 | XM_001120035.1 | FBgn0002932 | neur |
|  | AM07086 | GB14488 | XM_393828.2 | FBgn0040207 | kat80 |
|  | AM07281 | GB14684 |  | FBgn0051522 | CG31522 |
|  | AM07337 | GB14740 | XM_625249.2 | FBgn0032638 | CG6639 |
|  | AM07520 | GB14929 | XM_623051.2 | FBgn0052066 | CG32066 |
|  | AM07541 | GB14950 | XM_393674.2 | FBgn0001301 | kel |
|  | AM07675 |  |  |  |  |
|  | AM07682 | GB15091 | XM_397552.3 | FBgn0004870 | bab1 |
|  | AM07707 | GB15117 | XM_392463.3 | FBgn0000303 | Cha |
|  | AM07912 | GB15324 | XM_625100.2 | FBgn0033392 | CG8027 |
|  | AM08227 | GB15643 | XM_391982.3 | FBgn0033288 | pdm3 |
|  | AM08255 | GB15672 |  | FBgn0036316 | CG10960 |
|  | AM08433 | GB15851 | XM_392264.2 | FBgn0052447 | CG32447 |
|  | AM08555 | GB15974 | XM_623761.2 | FBgn0010825 | Gug |
|  | AM08914 |  |  |  |  |
|  | AM08981 | GB16400 | XM_392069.3 | FBgn0015331 | abs |
|  | AM08992 | GB16413 | XM_624876.2 | FBgn0032746 | CG10470 |
|  | AM09265 |  |  |  |  |
|  | AM09271 | GB16692 | XM_396566.3 | FBgn0039805 | CG12045 |
|  | AM09279 | GB16700 | XM_001120196.1 | FBgn0033450 | CG12924 |
|  | AM09504 | GB16927 | XM_397488.3 | FBgn0034797 | nahoda |
|  | AM09567 | GB16990 | XM_624379.2 | FBgn0024698 | cpsf |
|  | AM09829 | GB17254 | XM_392070.3 | FBgn0032151 | nAcRalpha-30D |
|  | AM09933 | GB17360 | XM_001121251.1 | FBgn0029152 | Mkrn1 |
|  | AM10298 | GB17725 | XM_623662.2 | FBgn0036570 | CG5222 |
|  | AM10453 | GB17882 | XM_392165.3 | FBgn0083962 | CG34126 |
|  | AM10534 |  |  |  |  |
|  | AM10787 | GB18221 |  | FBgn0034654 | CG10306 |
|  | AM10854 | GB18289 |  | FBgn0030940 | CG15040 |
|  | AM10998 | GB18435 | XM_394243.3 | FBgn0035101 | p130CAS |
|  | AM11076 | GB18514 |  | FBgn0038341 | CG14869 |
|  | AM11154 | GB18593 |  | FBgn0037807 | CG6293 |
|  | AM11198 | GB18637 | XM_393085.3 | FBgn0011570 | cpb |
|  | AM11202 |  |  |  |  |
|  | AM11315 | GB18755 | XM_394981.3 | FBgn0004176 | gammaTub23C |
|  | AM11517 |  |  |  |  |
|  | AM11586 | GB19031 |  | FBgn0032209 | Hand |
|  | AM11658 | GB19104 |  | FBgn0030715 | Or13a |
|  | AM11865 | GB19312 | XM_001120612.1 | FBgn0037299 | CG1115 |
|  | AM11972 | GB19420 | XM_393429.2 | FBgn0002938 | ninaC |
|  | AM12071 | GB19519 | XM_001122612.1 | FBgn0034978 | CG3257 |
|  | AM12422 | GB19875 | XM_001122114.1 | FBgn0034364 | CG5493 |
|  | AM12518 | GB19971 |  | FBgn0004618 | gl |
|  | AM12553 | GB20006 | XM_001122124.1 | FBgn0041161 | blue |
|  | AM12562 | GB20015 | XM_001122447.1 | FBgn0033869 | CG6305 |
|  | AM12672 | GB20125 | XM_623695.2 | FBgn0037652 | CG11980 |
|  | AM12743 |  |  |  |  |
|  | AM12756 | GB30320 |  | FBgn0045495 | Gr28b |
|  | AM12765 |  |  |  |  |
|  | AM12781 | GB30362 | XR_014951.1 | FBgn0002565 | Lsp2 |
|  | AM12840 |  |  |  |  |
|  | AM12851R | GB30268 | XM_624659.2 | FBgn0010226 | GstS1 |
|  | AM12899 |  |  |  |  |

**S4. Transcripts consistently regulated across both colonies.** - 360 transcripts were consistently up- or down-regulated in high responding individuals in both colonies.
