# Supplementary material for: Individual Variation in Pheromone Response Correlates with Reproductive Traits and Brain Gene Expression in Worker Honey Bees
Source: PLoS One. 2010 Feb 9;5(2):e9116. doi: 10.1371/journal.pone.0009116 (PMC2817734; doi:10.1371/journal.pone.0009116)
Supplement: Table S4 — Biological processes associated with retinue response. Gene ontology analysis of transcripts associated with retinue response. (0.03 MB DOC) [file pone.0009116.s005.doc]

| **GO Biological Process** | **p-value** |
| --- | --- |
| axonogenesis | <0.001 |
| neuron development | 0.001 |
| neuron differentiation | 0.002 |
| axon guidance | 0.007 |
| developmental process | 0.010 |
| response to toxin | 0.015 |
| immune response | 0.032 |
| carbohydrate metabolic process | 0.040 |
| response to chemical stimulus | 0.046 |
| response to temperature stimulus | 0.047 |

**S5. Biological processes associated with retinue response.** Gene ontology analysis of transcripts associated with retinue response.
