# Supplementary material for: Individual Variation in Pheromone Response Correlates with Reproductive Traits and Brain Gene Expression in Worker Honey Bees
Source: PLoS One. 2010 Feb 9;5(2):e9116. doi: 10.1371/journal.pone.0009116 (PMC2817734; doi:10.1371/journal.pone.0009116)
Supplement: Table S5 — Retinue response modules. Statistical gene networks predicted by MMC for retinue response. Each module was assigned an average degree of correlation among transcripts (avg degree), and each transcript received a degree of correlation between itself and the remaining transcripts from that module (degree). Gene ontologies associated with each module are also included in this table. (0.64 MB DOC) [file pone.0009116.s006.doc]

| ***GO Term*** | Module | Average Degree | Transcript | Degree |
| --- | --- | --- | --- | --- |
| ***axonogenesis*** | 1 | 0.77734 | AM04712 | 0.85614 |
|  |  |  | AM11881 | 0.84642 |
|  |  |  | AM04713 | 0.84244 |
|  |  |  | AM01799 | 0.84176 |
|  |  |  | AM04714 | 0.8329 |
|  |  |  | AM08374 | 0.8279 |
|  |  |  | AM03647 | 0.82624 |
|  |  |  | AM02864 | 0.81935 |
|  |  |  | AM08843 | 0.81729 |
|  |  |  | AM05950 | 0.81415 |
|  |  |  | AM02396 | 0.78597 |
|  |  |  | AM11373 | 0.77001 |
|  |  |  | AM02132 | 0.7689 |
|  |  |  | AM08991 | 0.75387 |
|  |  |  | AM10312 | 0.75267 |
|  |  |  | AM10040 | 0.73687 |
|  |  |  | AM11063 | 0.67167 |
|  |  |  | AM09121 | 0.66511 |
|  |  |  | AM04053 | 0.664 |
|  |  |  | AM10107 | 0.65308 |
| ***none*** | 2 | 0.74556 | AM09052 | 0.77662 |
|  |  |  | AM09529 | 0.76934 |
|  |  |  | AM10037 | 0.76296 |
|  |  |  | AM06062 | 0.75392 |
|  |  |  | AM04481 | 0.66495 |
| ***axonogenesis*** | 3 | 0.73227 | AM02021 | 0.82543 |
|  |  |  | AM12012 | 0.81791 |
|  |  |  | AM02259 | 0.81528 |
|  |  |  | AM12011 | 0.81385 |
|  |  |  | AM04734 | 0.80608 |
|  |  |  | AM08327 | 0.80309 |
|  |  |  | AM02967 | 0.79903 |
|  |  |  | AM03911 | 0.77267 |
|  |  |  | AM08088 | 0.77233 |
|  |  |  | AM09116 | 0.77225 |
|  |  |  | AM06086 | 0.77032 |
|  |  |  | AM04711 | 0.75671 |
|  |  |  | AM02196 | 0.75483 |
|  |  |  | AM00757R | 0.75303 |
|  |  |  | AM02588 | 0.74397 |
|  |  |  | AM08292 | 0.74348 |
|  |  |  | AM12037 | 0.73328 |
|  |  |  | AM10070 | 0.7281 |
|  |  |  | AM03528 | 0.72282 |
|  |  |  | AM07631 | 0.72186 |
|  |  |  | AM08746 | 0.7211 |
|  |  |  | AM09504 | 0.7151 |
|  |  |  | AM03187 | 0.71073 |
|  |  |  | AM03440 | 0.70022 |
|  |  |  | AM00558 | 0.67804 |
|  |  |  | AM10529 | 0.67356 |
|  |  |  | AM03211 | 0.66615 |
|  |  |  | AM12039 | 0.66225 |
|  |  |  | AM01881 | 0.6548 |
|  |  |  | AM02224 | 0.653 |
|  |  |  | AM11203 | 0.6523 |
|  |  |  | AM12723 | 0.51922 |
| ***glucose dehydrogenase (acceptor) activity*** | 4 | 0.69791 | AM11945 | 0.8035 |
|  |  |  | AM12251 | 0.78218 |
|  |  |  | AM02560 | 0.72338 |
|  |  |  | AM11970 | 0.71397 |
|  |  |  | AM12020 | 0.69314 |
|  |  |  | AM00109 | 0.64129 |
|  |  |  | AM12155 | 0.52788 |
| ***none*** | 5 | 0.68166 | AM00357 | 0.80117 |
|  |  |  | AM00356 | 0.7967 |
|  |  |  | AM00359 | 0.78923 |
|  |  |  | AM00360 | 0.77698 |
|  |  |  | AM00358 | 0.77439 |
|  |  |  | AM00351 | 0.77116 |
|  |  |  | AM00353 | 0.76003 |
|  |  |  | AM05351 | 0.7572 |
|  |  |  | AM06280 | 0.6372 |
|  |  |  | AM00244 | 0.58194 |
|  |  |  | AM06255 | 0.48587 |
|  |  |  | AM12637 | 0.47992 |
|  |  |  | AM08981 | 0.44975 |
| ***none*** | 6 | 0.67049 | AM01979 | 0.75976 |
|  |  |  | AM12218 | 0.757 |
|  |  |  | AM03848 | 0.7338 |
|  |  |  | AM11791 | 0.72599 |
|  |  |  | AM02635 | 0.63752 |
|  |  |  | AM01646 | 0.62796 |
|  |  |  | AM04117 | 0.59829 |
|  |  |  | AM11940 | 0.52362 |
| ***Synaptotagmin*** | 7 | 0.59496 | AM01394 | 0.71231 |
|  |  |  | AM02540 | 0.69754 |
|  |  |  | AM00529 | 0.66988 |
|  |  |  | AM09862 | 0.57665 |
|  |  |  | AM11228 | 0.46467 |
|  |  |  | AM10854 | 0.4487 |
| ***positive regulation of protein kinase activity*** | 8 | 0.5932 | AM01416 | 0.71892 |
|  |  |  | AM02160 | 0.71754 |
|  |  |  | AM01447 | 0.71102 |
|  |  |  | AM01737 | 0.70268 |
|  |  |  | AM02247 | 0.69421 |
|  |  |  | AM00717R | 0.68031 |
|  |  |  | AM01833 | 0.64567 |
|  |  |  | AM02442 | 0.63244 |
|  |  |  | AM06807 | 0.63009 |
|  |  |  | AM09953 | 0.62693 |
|  |  |  | AM09876 | 0.61704 |
|  |  |  | AM01667 | 0.61322 |
|  |  |  | AM05089 | 0.61177 |
|  |  |  | AM01996 | 0.59284 |
|  |  |  | AM01636 | 0.59083 |
|  |  |  | AM01313 | 0.55049 |
|  |  |  | AM07753 | 0.5377 |
|  |  |  | AM02496 | 0.52452 |
|  |  |  | AM05036 | 0.51309 |
|  |  |  | AM02398 | 0.43104 |
|  |  |  | AM08652 | 0.4094 |
|  |  |  | AM01892 | 0.29863 |
| ***neuron development*** | 9 | 0.57173 | AM01776 | 0.70346 |
|  |  |  | AM09023 | 0.69618 |
|  |  |  | AM02419 | 0.69279 |
|  |  |  | AM01474 | 0.6798 |
|  |  |  | AM12043 | 0.66801 |
|  |  |  | AM06852 | 0.66205 |
|  |  |  | AM02062 | 0.65934 |
|  |  |  | AM03236 | 0.65749 |
|  |  |  | AM07424 | 0.65742 |
|  |  |  | AM09861 | 0.65143 |
|  |  |  | AM11630 | 0.65094 |
|  |  |  | AM03506 | 0.64845 |
|  |  |  | AM00597 | 0.64695 |
|  |  |  | AM07127 | 0.64037 |
|  |  |  | AM09636 | 0.63895 |
|  |  |  | AM02418 | 0.63294 |
|  |  |  | AM02377 | 0.63256 |
|  |  |  | AM10908 | 0.63218 |
|  |  |  | AM04837 | 0.61009 |
|  |  |  | AM06128 | 0.60887 |
|  |  |  | AM10180 | 0.60874 |
|  |  |  | AM03826 | 0.59908 |
|  |  |  | AM03391 | 0.59675 |
|  |  |  | AM05614 | 0.59275 |
|  |  |  | AM05446 | 0.58512 |
|  |  |  | AM02366 | 0.58506 |
|  |  |  | AM01838 | 0.58425 |
|  |  |  | AM02757 | 0.58417 |
|  |  |  | AM03409 | 0.57733 |
|  |  |  | AM11759 | 0.57672 |
|  |  |  | AM07921 | 0.57284 |
|  |  |  | AM05928 | 0.5657 |
|  |  |  | AM02118 | 0.54214 |
|  |  |  | AM04409 | 0.53416 |
|  |  |  | AM06391 | 0.53168 |
|  |  |  | AM04102 | 0.51953 |
|  |  |  | AM01232 | 0.51498 |
|  |  |  | AM06118 | 0.49664 |
|  |  |  | AM06099 | 0.49113 |
|  |  |  | AM08934 | 0.48986 |
|  |  |  | AM07894 | 0.47288 |
|  |  |  | AM00464 | 0.45113 |
|  |  |  | AM01986 | 0.4317 |
|  |  |  | AM08796 | 0.36893 |
|  |  |  | AM07969 | 0.35504 |
|  |  |  | AM01334 | 0.29009 |
|  |  |  | AM10778 | 0.28265 |
| ***ion channel activity*** | 10 | 0.54729 | AM03136 | 0.68665 |
|  |  |  | AM12899 | 0.68278 |
|  |  |  | AM10534 | 0.67927 |
|  |  |  | AM11202 | 0.67583 |
|  |  |  | AM04423 | 0.66679 |
|  |  |  | AM01760 | 0.66423 |
|  |  |  | AM05467 | 0.66177 |
|  |  |  | AM06657 | 0.65062 |
|  |  |  | AM00333 | 0.64877 |
|  |  |  | AM09271 | 0.64178 |
|  |  |  | AM08227 | 0.63965 |
|  |  |  | AM05655 | 0.6287 |
|  |  |  | AM03091 | 0.62363 |
|  |  |  | AM04186 | 0.60378 |
|  |  |  | AM09829 | 0.5881 |
|  |  |  | AM07682 | 0.58758 |
|  |  |  | AM02766 | 0.58756 |
|  |  |  | AM07541 | 0.57506 |
|  |  |  | AM00459R | 0.57307 |
|  |  |  | AM05426 | 0.57268 |
|  |  |  | AM12562 | 0.56745 |
|  |  |  | AM04469 | 0.56361 |
|  |  |  | AM03624 | 0.55992 |
|  |  |  | AM02537 | 0.55415 |
|  |  |  | AM03879 | 0.54132 |
|  |  |  | AM06315 | 0.54095 |
|  |  |  | AM01296 | 0.52942 |
|  |  |  | AM04906 | 0.51528 |
|  |  |  | AM04427 | 0.49773 |
|  |  |  | AM12422 | 0.4968 |
|  |  |  | AM01818 | 0.47839 |
|  |  |  | AM02158 | 0.46856 |
|  |  |  | AM06980 | 0.45936 |
|  |  |  | AM11972 | 0.44792 |
|  |  |  | AM12852R | 0.42756 |
|  |  |  | AM00763 | 0.42526 |
|  |  |  | AM10787 | 0.41683 |
|  |  |  | AM11865 | 0.39473 |
|  |  |  | AM01138 | 0.38645 |
|  |  |  | AM07675 | 0.32722 |
|  |  |  | AM11412 | 0.20152 |
| ***vesicle-mediated transport*** | 11 | 0.34524 | AM03024 | 0.48493 |
|  |  |  | AM01004 | 0.48199 |
|  |  |  | AM04620 | 0.47823 |
|  |  |  | AM01368 | 0.47821 |
|  |  |  | AM01798 | 0.47727 |
|  |  |  | AM09755 | 0.47585 |
|  |  |  | AM02542 | 0.47292 |
|  |  |  | AM07873 | 0.47132 |
|  |  |  | AM01403 | 0.46688 |
|  |  |  | AM01558 | 0.46242 |
|  |  |  | AM02522 | 0.46233 |
|  |  |  | AM01631 | 0.45853 |
|  |  |  | AM04436 | 0.4529 |
|  |  |  | AM02324 | 0.45254 |
|  |  |  | AM02746 | 0.45178 |
|  |  |  | AM07292 | 0.44692 |
|  |  |  | AM08998 | 0.43244 |
|  |  |  | AM01093 | 0.42934 |
|  |  |  | AM03982 | 0.42543 |
|  |  |  | AM01490 | 0.425 |
|  |  |  | AM01880 | 0.41742 |
|  |  |  | AM00954 | 0.41511 |
|  |  |  | AM01977 | 0.40673 |
|  |  |  | AM06368 | 0.40321 |
|  |  |  | AM06152 | 0.40218 |
|  |  |  | AM05615 | 0.40053 |
|  |  |  | AM01596 | 0.39003 |
|  |  |  | AM04029 | 0.3898 |
|  |  |  | AM01804 | 0.38901 |
|  |  |  | AM00733 | 0.38663 |
|  |  |  | AM02725 | 0.37964 |
|  |  |  | AM09733 | 0.37601 |
|  |  |  | AM04609 | 0.37596 |
|  |  |  | AM04196 | 0.37405 |
|  |  |  | AM05999 | 0.37383 |
|  |  |  | AM10510 | 0.3704 |
|  |  |  | AM02936 | 0.36261 |
|  |  |  | AM12553 | 0.36154 |
|  |  |  | AM01508 | 0.36153 |
|  |  |  | AM12207 | 0.35992 |
|  |  |  | AM09441 | 0.34229 |
|  |  |  | AM07579 | 0.34122 |
|  |  |  | AM05570 | 0.33977 |
|  |  |  | AM01159 | 0.33574 |
|  |  |  | AM07470 | 0.33389 |
|  |  |  | AM01178 | 0.32741 |
|  |  |  | AM08306 | 0.32724 |
|  |  |  | AM01423 | 0.31915 |
|  |  |  | AM01252 | 0.31741 |
|  |  |  | AM01515 | 0.30053 |
|  |  |  | AM07912 | 0.29894 |
|  |  |  | AM02201 | 0.29828 |
|  |  |  | AM04953 | 0.29816 |
|  |  |  | AM12756 | 0.2964 |
|  |  |  | AM06220 | 0.295 |
|  |  |  | AM11403 | 0.29099 |
|  |  |  | AM02128 | 0.28898 |
|  |  |  | AM04610 | 0.27833 |
|  |  |  | AM04283 | 0.27754 |
|  |  |  | AM03208 | 0.27742 |
|  |  |  | AM00378 | 0.27337 |
|  |  |  | AM08433 | 0.26159 |
|  |  |  | AM12844R | 0.25778 |
|  |  |  | AM10160 | 0.25696 |
|  |  |  | AM03623 | 0.25406 |
|  |  |  | AM12097 | 0.25353 |
|  |  |  | AM01112 | 0.25288 |
|  |  |  | AM01177 | 0.23815 |
|  |  |  | AM09692 | 0.22783 |
|  |  |  | AM05356 | 0.22626 |
|  |  |  | AM09209 | 0.22108 |
|  |  |  | AM04363 | 0.20849 |
|  |  |  | AM09654 | 0.2058 |
|  |  |  | AM11541 | 0.20047 |
|  |  |  | AM01340 | 0.19509 |
|  |  |  | AM11602 | 0.18874 |
|  |  |  | AM01529 | 0.18801 |
|  |  |  | AM01193 | 0.1509 |
|  |  |  | AM11705 | 0.14467 |
| ***SNF-2 related*** | 12 | 0.23863 | AM04978 | 0.35302 |
|  |  |  | AM10504 | 0.34986 |
|  |  |  | AM08490 | 0.34277 |
|  |  |  | AM02855 | 0.33976 |
|  |  |  | AM07863 | 0.3379 |
|  |  |  | AM11838 | 0.33479 |
|  |  |  | AM12344 | 0.33385 |
|  |  |  | AM07169 | 0.33303 |
|  |  |  | AM12095 | 0.33116 |
|  |  |  | AM04187 | 0.32997 |
|  |  |  | AM01889 | 0.32981 |
|  |  |  | AM06137 | 0.32604 |
|  |  |  | AM06954 | 0.32595 |
|  |  |  | AM03284 | 0.32558 |
|  |  |  | AM01542 | 0.32419 |
|  |  |  | AM11977 | 0.32359 |
|  |  |  | AM07368 | 0.32078 |
|  |  |  | AM03370 | 0.31952 |
|  |  |  | AM06192 | 0.3192 |
|  |  |  | AM12558 | 0.31898 |
|  |  |  | AM09662 | 0.31847 |
|  |  |  | AM03485 | 0.31814 |
|  |  |  | AM08989 | 0.31756 |
|  |  |  | AM12288 | 0.31713 |
|  |  |  | AM10178 | 0.3169 |
|  |  |  | AM05014 | 0.31493 |
|  |  |  | AM01766 | 0.31219 |
|  |  |  | AM12850R | 0.31192 |
|  |  |  | AM12055 | 0.3102 |
|  |  |  | AM08642 | 0.30957 |
|  |  |  | AM01973 | 0.30956 |
|  |  |  | AM12340 | 0.30949 |
|  |  |  | AM03883 | 0.30942 |
|  |  |  | AM06886 | 0.30926 |
|  |  |  | AM01365 | 0.30917 |
|  |  |  | AM09950 | 0.30886 |
|  |  |  | AM03457 | 0.30796 |
|  |  |  | AM10351 | 0.30788 |
|  |  |  | AM01901 | 0.30587 |
|  |  |  | AM03843 | 0.3057 |
|  |  |  | AM06300 | 0.30417 |
|  |  |  | AM10260 | 0.30389 |
|  |  |  | AM11498 | 0.30359 |
|  |  |  | AM04626 | 0.30337 |
|  |  |  | AM01249 | 0.3026 |
|  |  |  | AM03005 | 0.30238 |
|  |  |  | AM03116 | 0.30114 |
|  |  |  | AM04926 | 0.29893 |
|  |  |  | AM03396 | 0.29872 |
|  |  |  | AM07823 | 0.29783 |
|  |  |  | AM05345 | 0.29721 |
|  |  |  | AM05480 | 0.29685 |
|  |  |  | AM11376 | 0.29617 |
|  |  |  | AM09881 | 0.29608 |
|  |  |  | AM06892 | 0.29576 |
|  |  |  | AM07128 | 0.29564 |
|  |  |  | AM02481 | 0.29525 |
|  |  |  | AM07474 | 0.29497 |
|  |  |  | AM09211 | 0.2947 |
|  |  |  | AM10931 | 0.29412 |
|  |  |  | AM03512 | 0.29396 |
|  |  |  | AM11260 | 0.29337 |
|  |  |  | AM12159 | 0.29309 |
|  |  |  | AM02488 | 0.29286 |
|  |  |  | AM06436 | 0.29134 |
|  |  |  | AM08958 | 0.29117 |
|  |  |  | AM09506 | 0.29061 |
|  |  |  | AM03884 | 0.29007 |
|  |  |  | AM08681 | 0.28952 |
|  |  |  | AM07975 | 0.28916 |
|  |  |  | AM12583 | 0.28843 |
|  |  |  | AM09888 | 0.28838 |
|  |  |  | AM02161 | 0.28833 |
|  |  |  | AM08990 | 0.28657 |
|  |  |  | AM01939 | 0.28643 |
|  |  |  | AM08915 | 0.28559 |
|  |  |  | AM12845R | 0.28451 |
|  |  |  | AM06095 | 0.28412 |
|  |  |  | AM10124 | 0.28384 |
|  |  |  | AM09547 | 0.28322 |
|  |  |  | AM08960 | 0.28254 |
|  |  |  | AM12844 | 0.28231 |
|  |  |  | AM12312 | 0.28159 |
|  |  |  | AM09002 | 0.28079 |
|  |  |  | AM12083 | 0.28003 |
|  |  |  | AM04600 | 0.27863 |
|  |  |  | AM10164 | 0.27843 |
|  |  |  | AM12768 | 0.27834 |
|  |  |  | AM11315 | 0.27687 |
|  |  |  | AM04987 | 0.27522 |
|  |  |  | AM06762 | 0.27442 |
|  |  |  | AM02814 | 0.27413 |
|  |  |  | AM02061 | 0.27373 |
|  |  |  | AM11565 | 0.27263 |
|  |  |  | AM10535 | 0.27155 |
|  |  |  | AM03126 | 0.26967 |
|  |  |  | AM05931 | 0.26931 |
|  |  |  | AM00366 | 0.26885 |
|  |  |  | AM12699 | 0.26856 |
|  |  |  | AM02585 | 0.26849 |
|  |  |  | AM06524 | 0.26837 |
|  |  |  | AM05608 | 0.26682 |
|  |  |  | AM09663 | 0.26667 |
|  |  |  | AM11902 | 0.2662 |
|  |  |  | AM00070 | 0.26619 |
|  |  |  | AM11529 | 0.26587 |
|  |  |  | AM03885 | 0.26586 |
|  |  |  | AM01921 | 0.26545 |
|  |  |  | AM04943 | 0.26511 |
|  |  |  | AM10471 | 0.26437 |
|  |  |  | AM12353 | 0.26424 |
|  |  |  | AM01304 | 0.26375 |
|  |  |  | AM06267 | 0.26293 |
|  |  |  | AM04309 | 0.26142 |
|  |  |  | AM02897 | 0.26087 |
|  |  |  | AM09834 | 0.26031 |
|  |  |  | AM03470 | 0.26004 |
|  |  |  | AM09945 | 0.25808 |
|  |  |  | AM10186 | 0.25704 |
|  |  |  | AM12156 | 0.25686 |
|  |  |  | AM05971 | 0.2568 |
|  |  |  | AM02115 | 0.25648 |
|  |  |  | AM04739 | 0.25555 |
|  |  |  | AM03132 | 0.25538 |
|  |  |  | AM07032 | 0.25456 |
|  |  |  | AM12617 | 0.25453 |
|  |  |  | AM12382 | 0.25397 |
|  |  |  | AM01877 | 0.25391 |
|  |  |  | AM05893 | 0.25251 |
|  |  |  | AM11030 | 0.25221 |
|  |  |  | AM10555 | 0.2517 |
|  |  |  | AM09749 | 0.25079 |
|  |  |  | AM01037 | 0.25075 |
|  |  |  | AM12801 | 0.25042 |
|  |  |  | AM01763 | 0.2503 |
|  |  |  | AM01108 | 0.25014 |
|  |  |  | AM09698 | 0.24965 |
|  |  |  | AM09724 | 0.24946 |
|  |  |  | AM05929 | 0.24923 |
|  |  |  | AM03176 | 0.24824 |
|  |  |  | AM01493 | 0.24807 |
|  |  |  | AM12746 | 0.24763 |
|  |  |  | AM09801 | 0.24752 |
|  |  |  | AM07846 | 0.24724 |
|  |  |  | AM05960 | 0.24601 |
|  |  |  | AM11812 | 0.24569 |
|  |  |  | AM02315 | 0.24558 |
|  |  |  | AM01391 | 0.24485 |
|  |  |  | AM11951 | 0.24481 |
|  |  |  | AM12811 | 0.24435 |
|  |  |  | AM02135 | 0.24434 |
|  |  |  | AM00598 | 0.24417 |
|  |  |  | AM00408 | 0.24401 |
|  |  |  | AM00045 | 0.24361 |
|  |  |  | AM12401 | 0.24355 |
|  |  |  | AM01845 | 0.24296 |
|  |  |  | AM10628 | 0.24283 |
|  |  |  | AM07583 | 0.24265 |
|  |  |  | AM02239 | 0.24188 |
|  |  |  | AM00417R | 0.24177 |
|  |  |  | AM07607 | 0.24149 |
|  |  |  | AM01619 | 0.2412 |
|  |  |  | AM07409 | 0.24098 |
|  |  |  | AM01524 | 0.23971 |
|  |  |  | AM12813 | 0.23935 |
|  |  |  | AM08593 | 0.23909 |
|  |  |  | AM11934 | 0.23902 |
|  |  |  | AM01686 | 0.23889 |
|  |  |  | AM11885 | 0.23887 |
|  |  |  | AM07299 | 0.23866 |
|  |  |  | AM10119 | 0.23849 |
|  |  |  | AM00894 | 0.23798 |
|  |  |  | AM00626R | 0.23782 |
|  |  |  | AM04470 | 0.23701 |
|  |  |  | AM05827 | 0.2369 |
|  |  |  | AM06370 | 0.23608 |
|  |  |  | AM09645 | 0.23605 |
|  |  |  | AM12390 | 0.23548 |
|  |  |  | AM02567 | 0.23529 |
|  |  |  | AM00904 | 0.23492 |
|  |  |  | AM01246 | 0.23453 |
|  |  |  | AM07868 | 0.23377 |
|  |  |  | AM12845 | 0.23371 |
|  |  |  | AM02120 | 0.23361 |
|  |  |  | AM08625 | 0.23353 |
|  |  |  | AM12840 | 0.23334 |
|  |  |  | AM12864R | 0.23318 |
|  |  |  | AM09157 | 0.23267 |
|  |  |  | AM01722 | 0.23184 |
|  |  |  | AM01228 | 0.23157 |
|  |  |  | AM12851R | 0.23076 |
|  |  |  | AM07609 | 0.2305 |
|  |  |  | AM07033 | 0.2305 |
|  |  |  | AM02367 | 0.23003 |
|  |  |  | AM09197 | 0.2299 |
|  |  |  | AM12864 | 0.22988 |
|  |  |  | AM04113 | 0.22967 |
|  |  |  | AM08572 | 0.22949 |
|  |  |  | AM08952 | 0.22942 |
|  |  |  | AM05524 | 0.22925 |
|  |  |  | AM12318 | 0.22921 |
|  |  |  | AM06178 | 0.22879 |
|  |  |  | AM02625 | 0.22821 |
|  |  |  | AM07520 | 0.22787 |
|  |  |  | AM07565 | 0.2274 |
|  |  |  | AM08987 | 0.22701 |
|  |  |  | AM09536 | 0.22695 |
|  |  |  | AM10679 | 0.22686 |
|  |  |  | AM03792 | 0.22681 |
|  |  |  | AM07015 | 0.22667 |
|  |  |  | AM05998 | 0.22597 |
|  |  |  | AM11745 | 0.22579 |
|  |  |  | AM00496 | 0.22539 |
|  |  |  | AM10436 | 0.22535 |
|  |  |  | AM11638 | 0.22514 |
|  |  |  | AM10123 | 0.2243 |
|  |  |  | AM03198 | 0.22418 |
|  |  |  | AM02013 | 0.22401 |
|  |  |  | AM12256 | 0.2227 |
|  |  |  | AM01324 | 0.22249 |
|  |  |  | AM00830 | 0.22241 |
|  |  |  | AM06135 | 0.22202 |
|  |  |  | AM08140 | 0.22193 |
|  |  |  | AM00439R | 0.22176 |
|  |  |  | AM04439 | 0.22169 |
|  |  |  | AM02832 | 0.22141 |
|  |  |  | AM08883 | 0.22141 |
|  |  |  | AM07189 | 0.22139 |
|  |  |  | AM02764 | 0.22125 |
|  |  |  | AM06589 | 0.22111 |
|  |  |  | AM11156 | 0.22042 |
|  |  |  | AM12843R | 0.22035 |
|  |  |  | AM10179 | 0.22032 |
|  |  |  | AM04205 | 0.22029 |
|  |  |  | AM01519 | 0.22008 |
|  |  |  | AM02162 | 0.21982 |
|  |  |  | AM02006 | 0.21933 |
|  |  |  | AM11416 | 0.21886 |
|  |  |  | AM05497 | 0.21886 |
|  |  |  | AM05067 | 0.21877 |
|  |  |  | AM04550 | 0.21875 |
|  |  |  | AM03795 | 0.21847 |
|  |  |  | AM05338 | 0.21846 |
|  |  |  | AM08740 | 0.21785 |
|  |  |  | AM05454 | 0.21777 |
|  |  |  | AM05710 | 0.21749 |
|  |  |  | AM05319 | 0.21733 |
|  |  |  | AM05658 | 0.21655 |
|  |  |  | AM01994 | 0.2164 |
|  |  |  | AM00446 | 0.21629 |
|  |  |  | AM10238 | 0.21585 |
|  |  |  | AM07978 | 0.21541 |
|  |  |  | AM03617 | 0.21541 |
|  |  |  | AM01005 | 0.21529 |
|  |  |  | AM04964 | 0.21519 |
|  |  |  | AM11637 | 0.21468 |
|  |  |  | AM01196 | 0.21466 |
|  |  |  | AM07683 | 0.21375 |
|  |  |  | AM00737 | 0.21341 |
|  |  |  | AM09624 | 0.21306 |
|  |  |  | AM11198 | 0.21306 |
|  |  |  | AM10135 | 0.21291 |
|  |  |  | AM01255 | 0.21251 |
|  |  |  | AM11450 | 0.2125 |
|  |  |  | AM02412 | 0.21239 |
|  |  |  | AM08407 | 0.21233 |
|  |  |  | AM12100 | 0.21017 |
|  |  |  | AM08024 | 0.20932 |
|  |  |  | AM02144 | 0.20905 |
|  |  |  | AM11154 | 0.20779 |
|  |  |  | AM03644 | 0.20747 |
|  |  |  | AM03295 | 0.20729 |
|  |  |  | AM11418 | 0.20704 |
|  |  |  | AM12743 | 0.20689 |
|  |  |  | AM08245 | 0.20687 |
|  |  |  | AM04989 | 0.20671 |
|  |  |  | AM09241 | 0.20657 |
|  |  |  | AM04434 | 0.20653 |
|  |  |  | AM07274 | 0.20611 |
|  |  |  | AM07946 | 0.20607 |
|  |  |  | AM08992 | 0.20574 |
|  |  |  | AM08260 | 0.20511 |
|  |  |  | AM00612 | 0.2049 |
|  |  |  | AM03421 | 0.20489 |
|  |  |  | AM00523 | 0.20476 |
|  |  |  | AM09533 | 0.20454 |
|  |  |  | AM00859 | 0.20424 |
|  |  |  | AM05177 | 0.20389 |
|  |  |  | AM05111 | 0.20386 |
|  |  |  | AM05189 | 0.20382 |
|  |  |  | AM03854 | 0.20374 |
|  |  |  | AM05728 | 0.2031 |
|  |  |  | AM11405 | 0.20265 |
|  |  |  | AM02954 | 0.20265 |
|  |  |  | AM04023 | 0.20259 |
|  |  |  | AM04650 | 0.2025 |
|  |  |  | AM11125 | 0.20164 |
|  |  |  | AM01427 | 0.20153 |
|  |  |  | AM12033 | 0.20072 |
|  |  |  | AM04707 | 0.20037 |
|  |  |  | AM03128 | 0.20004 |
|  |  |  | AM10161 | 0.19979 |
|  |  |  | AM09699 | 0.19937 |
|  |  |  | AM03618 | 0.19889 |
|  |  |  | AM04843 | 0.19848 |
|  |  |  | AM01335 | 0.19842 |
|  |  |  | AM11318 | 0.19692 |
|  |  |  | AM08895 | 0.19662 |
|  |  |  | AM06718 | 0.19661 |
|  |  |  | AM05726 | 0.19653 |
|  |  |  | AM04979 | 0.19645 |
|  |  |  | AM00823 | 0.19644 |
|  |  |  | AM11076 | 0.1964 |
|  |  |  | AM03767 | 0.19622 |
|  |  |  | AM02439 | 0.19604 |
|  |  |  | AM05638 | 0.19593 |
|  |  |  | AM05215 | 0.19564 |
|  |  |  | AM02110 | 0.19514 |
|  |  |  | AM02333 | 0.19479 |
|  |  |  | AM06400 | 0.19422 |
|  |  |  | AM04618 | 0.19368 |
|  |  |  | AM09265 | 0.19348 |
|  |  |  | AM11573 | 0.19332 |
|  |  |  | AM07405 | 0.19263 |
|  |  |  | AM03021 | 0.19228 |
|  |  |  | AM06676 | 0.19069 |
|  |  |  | AM03777 | 0.18997 |
|  |  |  | AM07411 | 0.1897 |
|  |  |  | AM08466 | 0.18928 |
|  |  |  | AM05183 | 0.18871 |
|  |  |  | AM00463R | 0.18756 |
|  |  |  | AM06117 | 0.1873 |
|  |  |  | AM06650 | 0.18729 |
|  |  |  | AM10281 | 0.18708 |
|  |  |  | AM02617 | 0.18671 |
|  |  |  | AM02125 | 0.1867 |
|  |  |  | AM11092 | 0.18573 |
|  |  |  | AM00951 | 0.18569 |
|  |  |  | AM08345 | 0.18542 |
|  |  |  | AM02768 | 0.18504 |
|  |  |  | AM04098 | 0.18452 |
|  |  |  | AM01607 | 0.18416 |
|  |  |  | AM04828 | 0.18375 |
|  |  |  | AM07624 | 0.18027 |
|  |  |  | AM03065 | 0.18023 |
|  |  |  | AM00464R | 0.17747 |
|  |  |  | AM07360 | 0.17694 |
|  |  |  | AM03530 | 0.17682 |
|  |  |  | AM10433 | 0.17682 |
|  |  |  | AM01053 | 0.17678 |
|  |  |  | AM05934 | 0.17568 |
|  |  |  | AM10298 | 0.17567 |
|  |  |  | AM09674 | 0.17552 |
|  |  |  | AM10606 | 0.1744 |
|  |  |  | AM09669 | 0.17418 |
|  |  |  | AM10939 | 0.17348 |
|  |  |  | AM02742 | 0.17211 |
|  |  |  | AM02706 | 0.17073 |
|  |  |  | AM11495 | 0.17052 |
|  |  |  | AM02154 | 0.16922 |
|  |  |  | AM09503 | 0.1679 |
|  |  |  | AM00787 | 0.16729 |
|  |  |  | AM12765 | 0.16609 |
|  |  |  | AM01033 | 0.16566 |
|  |  |  | AM01043 | 0.16322 |
|  |  |  | AM04830 | 0.16258 |
|  |  |  | AM10104 | 0.16119 |
|  |  |  | AM12877R | 0.16018 |
|  |  |  | AM09642 | 0.1589 |
|  |  |  | AM10893 | 0.15818 |
|  |  |  | AM04460 | 0.1574 |
|  |  |  | AM03822 | 0.15623 |
|  |  |  | AM11364 | 0.15574 |
|  |  |  | AM00073 | 0.15561 |
|  |  |  | AM06103 | 0.15362 |
|  |  |  | AM03234 | 0.14874 |
|  |  |  | AM03972 | 0.14781 |
|  |  |  | AM00429 | 0.14767 |
|  |  |  | AM12403 | 0.13187 |

**S6. Retinue response modules.** Statistical gene networks predicted by MMC for retinue response. Each module was assigned an average degree of correlation among transcripts (avg degree), and each transcript received a degree of correlation between itself and the remaining transcripts from that module (degree). Gene ontologies associated with each module are also included in this table.
