# Supplementary material for: Individual Variation in Pheromone Response Correlates with Reproductive Traits and Brain Gene Expression in Worker Honey Bees
Source: PLoS One. 2010 Feb 9;5(2):e9116. doi: 10.1371/journal.pone.0009116 (PMC2817734; doi:10.1371/journal.pone.0009116)
Supplement: Table S6 — Comparative genomic analysis. The 960 transcripts associated with retinue response were compared to previously published studies that identified sets of transcripts associated with other behavioral or physiological traits in workers. Because some of the 960 transcripts were up-regulated in high-responders of one colony and down-regulated in high-responders of the other colony, there is some overlap between gene lists. Overall, these patterns suggest that high-responding individuals have brain transcriptional profiles more similar to nurse bees than to forager bees. (0.04 MB DOC) [file pone.0009116.s007.doc]

| **Gene List** | **# Sig Transcripts** | **Up-regulated (819)** | **p-value** | **Down-regulated (600)** | **p-value** | **Reference** |
| --- | --- | --- | --- | --- | --- | --- |
| *QMP up-regulated* | 491 | 56 | 0.88 | 32 | 0.09 | *Grozinger et.al. 2003* |
| *QMP down-regulated* | 501 | 53 | 0.47 | 34 | 0.16 | *Grozinger et.al. 2003* |
| *Nursing-associated** | 599 | 61 | 0.26 | 36 | **0.02** | *Whitfield et.al. 2003* |
| *Foraging-associated** | 400 | 31 | **0.002** | 26 | 0.14 | *Whitfield et.al. 2003* |
| *Methoprene up-regulated** | 306 | 20 | **0.003** | 20 | 0.21 | *Whitfield et.al. 2006* |
| *Methoprene down-regulated* | 207 | 23 | 0.91 | 19 | 0.71 | *Whitfield et.al. 2006* |
| *Sterile > Reproductive* | 21 | 1 | 0.50 | 1 | 1.00 | *Grozinger et.al. 2007* |
| *Reproductive > Sterile* | 64 | 6 | 0.70 | 6 | 0.82 | *Grozinger et.al. 2007* |
| *Pollen hoarding QTL candidates* | 99 | 8 | 0.62 | 6 | 0.70 | *Hunt et.al 2007* |

**S7. Comparative genomic analysis.** The 960 transcripts associated with retinue response were compared to previously-published studies that identified sets of transcripts associated with other behavioral or physiological traits in workers. Because some of the 960 transcripts were up-regulated in high-responders of one colony and down-regulated in high-responders of the other colony, there is some overlap between gene lists. Overall, these patterns suggest that high-responding individuals have brain transcriptional profiles more similar to nurse bees than to forager bees.
