# Supplementary material for: Individual Variation in Pheromone Response Correlates with Reproductive Traits and Brain Gene Expression in Worker Honey Bees
Source: PLoS One. 2010 Feb 9;5(2):e9116. doi: 10.1371/journal.pone.0009116 (PMC2817734; doi:10.1371/journal.pone.0009116)
Supplement: Table S7 — Comparative studies gene lists. Significantly-regulated transcripts in this study were compared to previously published expression studies in honey bees. The list of overlapping transcripts are included in this file. (1.40 MB DOC) [file pone.0009116.s008.doc]

| **Transcript** | **Gene Name** | **Fly Ortholog** | **Grozinger 2003** | | **Whitfield 2003, nursing associated** | **Whitfield 2003, foraging associated** | **Whitfield 2006, Methoprene upregulated** | **Whitfield 2006, Methoprene down-regulated** | **Hunt 2007, Pollen QTL** | **N(Studies)** |
| --- | --- | --- | --- | --- | --- | --- | --- | --- | --- | --- |
| AM05728 | GB13120 | FucTA | | 1 | 1 |  |  | 1 |  | 3 |
| AM05764 | GB13155 | CG6847 | | 1 |  | 1 | 1 |  |  | 3 |
| AM06062 | GB13458 | und | | 1 | 1 |  |  | 1 |  | 3 |
| AM06118 | GB13516 | Glut1 | | 1 | 1 |  |  | 1 |  | 3 |
| AM06137 | GB13536 | CG11963 | | 1 |  | 1 | 1 |  |  | 3 |
| AM07520 | GB14929 | CG32066 | | 1 |  | 1 | 1 |  |  | 3 |
| AM08989 | GB16408 | nemy | | 1 |  | 1 |  | 1 |  | 3 |
| AM09781 | GB17206 | CG9743 | | 1 | 1 |  | 1 |  |  | 3 |
| AM09953 | GB17380 | fax | | 1 | 1 |  |  | 1 |  | 3 |
| AM12344 | GB19797 | Cyp6a2 | | 1 | 1 |  |  | 1 |  | 3 |
| AM05153 | GB12537 | CG14321 | |  | 1 |  |  |  |  | 1 |
| AM08407 | GB15825 | CG10139 | | 1 |  |  |  |  |  | 1 |
| AM02832 | GB10205 | l(3)IX-14 | | 1 |  |  | 1 |  |  | 2 |
| AM02834 | GB10207 | Vps16A | | 1 | 1 |  |  |  |  | 2 |
| AM03116 | GB10489 | nrv3 | | 1 |  |  | 1 |  |  | 2 |
| AM03284 | GB10657 | ScpX | | 1 |  |  | 1 |  |  | 2 |
| AM03409 | GB10784 | SP2637 | | 1 |  |  |  | 1 |  | 2 |
| AM03420 | GB10795 | CG8839 | | 1 | 1 |  |  |  |  | 2 |
| AM03885 | GB11262 | Scgalpha | | 1 | 1 |  |  |  |  | 2 |
| AM03982 | GB11361 | CG3036 | |  | 1 |  |  | 1 |  | 2 |
| AM04025 | GB11404 | trc | | 1 |  |  |  | 1 |  | 2 |
| AM04072 | GB11451 | CG10641 | | 1 |  |  |  | 1 |  | 2 |
| AM04439 | GB11820 | Ipp | | 1 | 1 |  |  |  |  | 2 |
| AM04609 | GB11992 | CG4065 | | 1 |  |  |  |  | 1 | 2 |
| AM04621 | GB12004 | Jra | | 1 |  | 1 |  |  |  | 2 |
| AM04650 | GB12033 | CG32687 | | 1 | 1 |  |  |  |  | 2 |
| AM04734 | GB12113 | porin | | 1 | 1 |  |  |  |  | 2 |
| AM05177 | GB12561 | garz | | 1 |  |  | 1 |  |  | 2 |
| AM05189 | GB12573 | l(1)G0030 | |  |  | 1 | 1 |  |  | 2 |
| AM05460 | GB12846 | CG32048 | | 1 |  | 1 |  |  |  | 2 |
| AM05570 | GB12957 | CG4090 | | 1 | 1 |  |  |  |  | 2 |
| AM06192 | GB13592 | Klc | | 1 |  | 1 |  |  |  | 2 |
| AM06220 | GB13621 | CG32250 | | 1 |  |  |  |  | 1 | 2 |
| AM06625 | GB14026 | inx2 | | 1 | 1 |  |  |  |  | 2 |
| AM07032 | GB14434 | Lsd-2 | |  |  | 1 | 1 |  |  | 2 |
| AM07337 | GB14740 | CG6639 | | 1 |  |  | 1 |  |  | 2 |
| AM07367 | GB14772 | Src64B | | 1 |  | 1 |  |  |  | 2 |
| AM07405 | GB14810 | Hel89B | | 1 | 1 |  |  |  |  | 2 |
| AM07631 | GB15040 | CG32131 | | 1 |  | 1 |  |  |  | 2 |
| AM07697 | GB15106 | CG11159 | | 1 | 1 |  |  |  |  | 2 |
| AM07975 | GB15387 | janA | | 1 | 1 |  |  |  |  | 2 |
| AM08067 | GB15481 | CG11926 | | 1 |  | 1 |  |  |  | 2 |
| AM08245 | GB15662 | CG10962 | | 1 | 1 |  |  |  |  | 2 |
| AM08260 | GB15677 | PH4alphaEFB | | 1 | 1 |  |  |  |  | 2 |
| AM01138 | GB15745 | CG5618 | |  | 1 |  |  | 1 |  | 2 |
| AM08593 | GB16012 | rg | | 1 |  |  | 1 |  |  | 2 |
| AM08641 | GB16060 | CG12950 | |  | 1 |  |  | 1 |  | 2 |
| AM08740 | GB16162 | CG8498 | | 1 |  |  | 1 |  |  | 2 |
| AM08746 | GB16168 | tnc | |  | 1 |  |  | 1 |  | 2 |
| AM08958 | GB16377 | Eaat2 | | 1 |  |  | 1 |  |  | 2 |
| AM08991 | GB16412 | Tctp | |  | 1 |  |  | 1 |  | 2 |
| AM09023 | GB16444 | CG10737 | | 1 |  | 1 |  |  |  | 2 |
| AM09547 | GB16970 | Pli | | 1 | 1 |  |  |  |  | 2 |
| AM09669 | GB17094 | CG15786 | | 1 | 1 |  |  |  |  | 2 |
| AM09749 | GB17174 | CG3376 | | 1 |  | 1 |  |  |  | 2 |
| AM09861 | GB17286 | RpL13 | |  |  | 1 | 1 |  |  | 2 |
| AM10040 | GB17469 | shot | | 1 |  |  | 1 |  |  | 2 |
| AM10070 | GB17499 | sesB | |  | 1 |  | 1 |  |  | 2 |
| AM10312 | GB17739 | CG10376 | | 1 |  | 1 |  |  |  | 2 |
| AM10373 | GB17801 | CG9448 | | 1 | 1 |  |  |  |  | 2 |
| AM10397 | GB17826 | Rab10 | | 1 | 1 |  |  |  |  | 2 |
| AM10510 | GB17937 | CG2247 | | 1 |  | 1 |  |  |  | 2 |
| AM10535 | GB17962 | CG32638 | | 1 | 1 |  |  |  |  | 2 |
| AM10628 | GB18056 | CG5001 | | 1 |  |  |  | 1 |  | 2 |
| AM11203 | GB18642 | futsch | | 1 | 1 |  |  |  |  | 2 |
| AM11260 | GB18699 | CG8271 | |  |  | 1 | 1 |  |  | 2 |
| AM11940 | GB19387 | Hex-A | |  |  | 1 | 1 |  |  | 2 |
| AM11977 | GB19425 | Hop | | 1 |  | 1 |  |  |  | 2 |
| AM03295 | GB10669 | RpS16 | |  |  |  |  |  |  | 0 |
| AM06389 | GB13790 | CG18095 | |  |  |  |  |  |  | 0 |
| AM00688 |  |  | |  |  |  |  |  |  | 0 |
| AM01246 |  |  | |  |  |  |  |  |  | 0 |
| AM01515 |  |  | |  |  |  |  |  |  | 0 |
| AM01977 |  |  | |  |  |  |  |  |  | 0 |
| AM09157 |  |  | |  |  |  |  |  |  | 0 |
| AM12097 |  |  | |  |  |  |  |  |  | 0 |
| AM02746 | GB10118 | Nhe2 | | 1 |  |  |  |  |  | 1 |
| AM02766 | GB10138 | nompC | |  |  | 1 |  |  |  | 1 |
| AM02814 | GB10187 | px | |  |  | 1 |  |  |  | 1 |
| AM01490 | GB10237 | sif | | 1 |  |  |  |  |  | 1 |
| AM02757 | GB10294 | CG5731 | | 1 |  |  |  |  |  | 1 |
| AM03126 | GB10500 | Nle | |  | 1 |  |  |  |  | 1 |
| AM03333 | GB10708 | CG32055 | |  |  |  | 1 |  |  | 1 |
| AM03391 | GB10766 | Lar | |  |  | 1 |  |  |  | 1 |
| AM03440 | GB10815 | CG2201 | |  | 1 |  |  |  |  | 1 |
| AM03470 | GB10845 | PRL-1 | | 1 |  |  |  |  |  | 1 |
| AM03623 | GB10999 | DMAP1 | | 1 |  |  |  |  |  | 1 |
| AM03647 | GB11023 | CG3523 | |  |  |  |  | 1 |  | 1 |
| AM03731 | GB11107 | CG5366 | | 1 |  |  |  |  |  | 1 |
| AM03765 | GB11141 | lat | |  | 1 |  |  |  |  | 1 |
| AM03826 | GB11203 | Ard1 | |  | 1 |  |  |  |  | 1 |
| AM04117 | GB11497 | Unr | |  |  |  |  | 1 |  | 1 |
| AM04434 | GB11815 | CG12030 | |  |  | 1 |  |  |  | 1 |
| AM04436 | GB11817 | Atet | |  | 1 |  |  |  |  | 1 |
| AM04487 | GB11868 | Rab26 | |  | 1 |  |  |  |  | 1 |
| AM04550 | GB11933 | CG17904 | |  | 1 |  |  |  |  | 1 |
| AM04600 | GB11983 | Ance | | 1 |  |  |  |  |  | 1 |
| AM04702 | GB12085 | fau | |  |  | 1 |  |  |  | 1 |
| AM04926 | GB12307 | CG5087 | | 1 |  |  |  |  |  | 1 |
| AM05112 | GB12495 | CG13214 | |  |  |  | 1 |  |  | 1 |
| AM05132 | GB12515 | trus | |  |  | 1 |  |  |  | 1 |
| AM05168 | GB12553 | CG17471 | | 1 |  |  |  |  |  | 1 |
| AM05345 | GB12730 | CG5071 | | 1 |  |  |  |  |  | 1 |
| AM05467 | GB12853 | CadN | |  | 1 |  |  |  |  | 1 |
| AM05484 | GB12871 | Cam | |  |  | 1 |  |  |  | 1 |
| AM05614 | GB13003 | Pabp2 | |  |  | 1 |  |  |  | 1 |
| AM05615 | GB13004 | CG12163 | |  | 1 |  |  |  |  | 1 |
| AM05928 | GB13324 | Gtp-bp | | 1 |  |  |  |  |  | 1 |
| AM06045 | GB13441 | Ogt | |  |  | 1 |  |  |  | 1 |
| AM06128 | GB13526 | CG6914 | |  | 1 |  |  |  |  | 1 |
| AM06178 | GB13577 | Adar | | 1 |  |  |  |  |  | 1 |
| AM06194 | GB13594 | TpnC41C | |  |  |  | 1 |  |  | 1 |
| AM06204 | GB13604 | GABA-B-R2 | | 1 |  |  |  |  |  | 1 |
| AM06271 | GB13673 | Pkcdelta | | 1 |  |  |  |  |  | 1 |
| AM06281 | GB13683 | krz | | 1 |  |  |  |  |  | 1 |
| AM06370 | GB13771 | CG30296 | | 1 |  |  |  |  |  | 1 |
| AM06391 | GB13792 | mdy | |  |  |  |  |  | 1 | 1 |
| AM06525 | GB13927 | fl(2)d | | 1 |  |  |  |  |  | 1 |
| AM06718 | GB14118 | flfl | | 1 |  |  |  |  |  | 1 |
| AM06886 | GB14286 | CG2839 | |  | 1 |  |  |  |  | 1 |
| AM06972 | GB14374 | Pcmt | |  | 1 |  |  |  |  | 1 |
| AM07033 | GB14435 | l(2)efl | |  |  |  |  | 1 |  | 1 |
| AM07127 | GB14529 | Ptp61F | |  |  | 1 |  |  |  | 1 |
| AM07140 | GB14541 | dikar | | 1 |  |  |  |  |  | 1 |
| AM07205 | GB14608 | CG5375 | | 1 |  |  |  |  |  | 1 |
| AM07307 | GB14710 | CG4572 | |  | 1 |  |  |  |  | 1 |
| AM07368 | GB14773 | CG32663 | |  | 1 |  |  |  |  | 1 |
| AM07470 | GB14876 | CG3579 | |  | 1 |  |  |  |  | 1 |
| AM07549 | GB14958 | SelD | |  |  | 1 |  |  |  | 1 |
| AM07579 | GB14988 | CG31712 | |  | 1 |  |  |  |  | 1 |
| AM07591 | GB15000 | sgl | | 1 |  |  |  |  |  | 1 |
| AM07790 | GB15201 | CG10420 | |  |  |  |  |  | 1 | 1 |
| AM07837 | GB15248 | Vhl | | 1 |  |  |  |  |  | 1 |
| AM07846 | GB15257 | wfs1 | |  | 1 |  |  |  |  | 1 |
| AM07894 | GB15304 | CG13349 | | 1 |  |  |  |  |  | 1 |
| AM08024 | GB15437 | CG17034 | |  | 1 |  |  |  |  | 1 |
| AM08088 | GB15503 | RpL4 | |  |  |  |  | 1 |  | 1 |
| AM08227 | GB15643 | pdm3 | |  |  |  |  |  | 1 | 1 |
| AM08262 | GB15679 | Rep2 | |  | 1 |  |  |  |  | 1 |
| AM08493 | GB15912 | CG14509 | |  | 1 |  |  |  |  | 1 |
| AM08515 | GB15934 | CG13424 | |  |  |  |  |  | 1 | 1 |
| AM08681 | GB16101 | CG33229 | | 1 |  |  |  |  |  | 1 |
| AM08874 | GB16294 | Glycogenin | | 1 |  |  |  |  |  | 1 |
| AM08915 | GB16335 | Nlp | |  |  |  |  |  | 1 | 1 |
| AM09082 | GB16503 | CG31619 | | 1 |  |  |  |  |  | 1 |
| AM09241 | GB16663 | CG10971 | |  | 1 |  |  |  |  | 1 |
| AM09279 | GB16700 | CG12924 | |  |  | 1 |  |  |  | 1 |
| AM09441 | GB16864 | CG4389 | |  | 1 |  |  |  |  | 1 |
| AM09503 | GB16926 | RfC40 | |  | 1 |  |  |  |  | 1 |
| AM09536 | GB16959 | GstS1 | |  |  | 1 |  |  |  | 1 |
| AM09636 | GB17060 | CG15735 | | 1 |  |  |  |  |  | 1 |
| AM09644 | GB17068 | CG2663 | |  |  |  | 1 |  |  | 1 |
| AM09662 | GB17087 | CG13868 | |  |  |  | 1 |  |  | 1 |
| AM09674 | GB17099 | stc | | 1 |  |  |  |  |  | 1 |
| AM09724 | GB17149 | lbk | | 1 |  |  |  |  |  | 1 |
| AM09799 | GB17224 | CG7120 | |  | 1 |  |  |  |  | 1 |
| AM09834 | GB17259 | CG18646 | |  |  |  |  | 1 |  | 1 |
| AM09881 | GB17306 | CG15814 | |  |  |  |  |  | 1 | 1 |
| AM09933 | GB17360 | Mkrn1 | |  |  |  |  | 1 |  | 1 |
| AM09938 | GB17365 | slo | |  |  | 1 |  |  |  | 1 |
| AM10037 | GB17466 | CG10555 | |  | 1 |  |  |  |  | 1 |
| AM10104 | GB17533 | CG15093 | |  | 1 |  |  |  |  | 1 |
| AM10107 | GB17536 | CG6695 | | 1 |  |  |  |  |  | 1 |
| AM10436 | GB17865 | CG5934 | |  | 1 |  |  |  |  | 1 |
| AM10908 | GB18344 | CG9821 | | 1 |  |  |  |  |  | 1 |
| AM10931 | GB18367 | CG1105 | |  | 1 |  |  |  |  | 1 |
| AM11033 | GB18471 | GATAd | | 1 |  |  |  |  |  | 1 |
| AM11063 | GB18501 | CaBP1 | | 1 |  |  |  |  |  | 1 |
| AM11198 | GB18637 | cpb | | 1 |  |  |  |  |  | 1 |
| AM11364 | GB18806 | His2A:CG33862 | |  |  |  |  |  | 1 | 1 |
| AM11373 | GB18815 | Pp1alpha-96A | | 1 |  |  |  |  |  | 1 |
| AM11403 | GB18846 | CG2126 | |  | 1 |  |  |  |  | 1 |
| AM11471 | GB18914 | CG9050 | |  | 1 |  |  |  |  | 1 |
| AM11745 | GB19191 | Scgdelta | | 1 |  |  |  |  |  | 1 |
| AM11759 | GB19205 | CG9386 | |  | 1 |  |  |  |  | 1 |
| AM12033 | GB19481 | ND75 | |  |  |  | 1 |  |  | 1 |
| AM12037 | GB19485 | CG32486 | |  |  |  |  | 1 |  | 1 |
| AM12055 | GB19503 | Hsc70-4 | |  |  |  |  | 1 |  | 1 |
| AM12078 | GB19526 | CG14047 | | 1 |  |  |  |  |  | 1 |
| AM12083 | GB19531 | sec23 | | 1 |  |  |  |  |  | 1 |
| AM12218 | GB19670 | CG5840 | |  |  |  | 1 |  |  | 1 |
| AM12256 | GB19709 | Syt7 | |  |  | 1 |  |  |  | 1 |
| AM12340 | GB19793 | lama | |  |  |  |  | 1 |  | 1 |
| AM12353 | GB19806 | CG30118 | |  |  | 1 |  |  |  | 1 |
| AM12617 | GB20069 | CG5808 | | 1 |  |  |  |  |  | 1 |
| AM12691 | GB20144 | CG6972 | |  |  |  |  | 1 |  | 1 |
| AM02669 | GB10041 | CG8552 | |  |  |  |  |  |  | 0 |
| AM02706 | GB10078 | CG14470 | |  |  |  |  |  |  | 0 |
| AM02720 | GB10092 | l(1)G0060 | |  |  |  |  |  |  | 0 |
| AM02725 | GB10097 | CG6664 | |  |  |  |  |  |  | 0 |
| AM02742 | GB10114 | cbt | |  |  |  |  |  |  | 0 |
| AM02762 | GB10134 | su(w[a]) | |  |  |  |  |  |  | 0 |
| AM02764 | GB10136 | pain | |  |  |  |  |  |  | 0 |
| AM02768 | GB10140 | CG12173 | |  |  |  |  |  |  | 0 |
| AM02855 | GB10228 | CG1444 | |  |  |  |  |  |  | 0 |
| AM02863 | GB10236 | Hira | |  |  |  |  |  |  | 0 |
| AM02864 | GB10237 | sif | |  |  |  |  |  |  | 0 |
| AM02872 | GB10245 | CG2218 | |  |  |  |  |  |  | 0 |
| AM02910 | GB10280 | Slip1 | |  |  |  |  |  |  | 0 |
| AM02926 | GB10296 | CG6752 | |  |  |  |  |  |  | 0 |
| AM02936 | GB10306 | CG8679 | |  |  |  |  |  |  | 0 |
| AM02954 | GB10324 | CG32529 | |  |  |  |  |  |  | 0 |
| AM02967 | GB10337 | Ank2 | |  |  |  |  |  |  | 0 |
| AM02992 | GB10362 | Fer2 | |  |  |  |  |  |  | 0 |
| AM03005 | GB10376 | CG7510 | |  |  |  |  |  |  | 0 |
| AM03021 | GB10393 | CG10189 | |  |  |  |  |  |  | 0 |
| AM03024 | GB10396 | Gga | |  |  |  |  |  |  | 0 |
| AM03065 | GB10437 | Fur1 | |  |  |  |  |  |  | 0 |
| AM03091 | GB10464 | CG4743 | |  |  |  |  |  |  | 0 |
| AM03092 | GB10465 | srt | |  |  |  |  |  |  | 0 |
| AM03128 | GB10502 | CG32937 | |  |  |  |  |  |  | 0 |
| AM03136 | GB10509 | l(1)G0003 | |  |  |  |  |  |  | 0 |
| AM03176 | GB10549 | ss | |  |  |  |  |  |  | 0 |
| AM03181 | GB10554 | BBS8 | |  |  |  |  |  |  | 0 |
| AM03187 | GB10560 | Ef1alpha100E | |  |  |  |  |  |  | 0 |
| AM03198 | GB10571 | gce | |  |  |  |  |  |  | 0 |
| AM03208 | GB10581 | CG3156 | |  |  |  |  |  |  | 0 |
| AM03234 | GB10607 | Kap3 | |  |  |  |  |  |  | 0 |
| AM03236 | GB10608 | Mo25 | |  |  |  |  |  |  | 0 |
| AM03248 | GB10622 | yellow-h | |  |  |  |  |  |  | 0 |
| AM03251 | GB10625 | Mctp | |  |  |  |  |  |  | 0 |
| AM03283 | GB10656 | crol | |  |  |  |  |  |  | 0 |
| AM03370 | GB10745 | CG32103 | |  |  |  |  |  |  | 0 |
| AM03396 | GB10771 | Jheh1 | |  |  |  |  |  |  | 0 |
| AM03421 | GB10796 | Rpn12 | |  |  |  |  |  |  | 0 |
| AM03434 | GB10809 | CG9384 | |  |  |  |  |  |  | 0 |
| AM03457 | GB10832 | CG31650 | |  |  |  |  |  |  | 0 |
| AM03494 | GB10869 | Lsp2 | |  |  |  |  |  |  | 0 |
| AM03506 | GB10881 | CG17260 | |  |  |  |  |  |  | 0 |
| AM03512 | GB10887 | tefu | |  |  |  |  |  |  | 0 |
| AM03528 | GB10903 | RpL32 | |  |  |  |  |  |  | 0 |
| AM03530 | GB10905 | Cyp4c3 | |  |  |  |  |  |  | 0 |
| AM03560 | GB10935 | CG11802 | |  |  |  |  |  |  | 0 |
| AM03617 | GB10995 | CG33205 | |  |  |  |  |  |  | 0 |
| AM03618 | GB10995 | CG33205 | |  |  |  |  |  |  | 0 |
| AM03644 | GB11020 | CG6761 | |  |  |  |  |  |  | 0 |
| AM03646 | GB11022 | yellow-h | |  |  |  |  |  |  | 0 |
| AM03648 | GB11024 | CG11030 | |  |  |  |  |  |  | 0 |
| AM03767 | GB11143 | Csat | |  |  |  |  |  |  | 0 |
| AM03777 | GB11153 | smp-30 | |  |  |  |  |  |  | 0 |
| AM03792 | GB11168 | sr | |  |  |  |  |  |  | 0 |
| AM03795 | GB11171 | CLIP-190 | |  |  |  |  |  |  | 0 |
| AM03811 | GB11187 | Bap55 | |  |  |  |  |  |  | 0 |
| AM03820 | GB11197 | Dhc98D | |  |  |  |  |  |  | 0 |
| AM03822 | GB11199 | Strn-Mlck | |  |  |  |  |  |  | 0 |
| AM03843 | GB11220 | CG2791 | |  |  |  |  |  |  | 0 |
| AM03848 | GB11225 | CG6903 | |  |  |  |  |  |  | 0 |
| AM03854 | GB11231 | CG32495 | |  |  |  |  |  |  | 0 |
| AM03879 | GB11256 | CG17292 | |  |  |  |  |  |  | 0 |
| AM03883 | GB11260 | Rpn5 | |  |  |  |  |  |  | 0 |
| AM03884 | GB11261 | CG4673 | |  |  |  |  |  |  | 0 |
| AM03899 | GB11276 | CG31183 | |  |  |  |  |  |  | 0 |
| AM03911 | GB11288 | syt | |  |  |  |  |  |  | 0 |
| AM03980 | GB11359 | Aats-met | |  |  |  |  |  |  | 0 |
| AM04023 | GB11402 | WASp | |  |  |  |  |  |  | 0 |
| AM04029 | GB11408 | mask | |  |  |  |  |  |  | 0 |
| AM04066 | GB11445 | CG12769 | |  |  |  |  |  |  | 0 |
| AM04073 | GB11452 | MICAL | |  |  |  |  |  |  | 0 |
| AM04098 | GB11477 | hgo | |  |  |  |  |  |  | 0 |
| AM04102 | GB11481 | CG12340 | |  |  |  |  |  |  | 0 |
| AM04113 | GB11493 | CG14608 | |  |  |  |  |  |  | 0 |
| AM04133 | GB11513 | SRm160 | |  |  |  |  |  |  | 0 |
| AM04186 | GB11566 | oc | |  |  |  |  |  |  | 0 |
| AM04187 | GB11567 | Flo | |  |  |  |  |  |  | 0 |
| AM04196 | GB11576 | CG7380 | |  |  |  |  |  |  | 0 |
| AM04229 | GB11609 | CG5053 | |  |  |  |  |  |  | 0 |
| AM04245 | GB11625 | Sema-5c | |  |  |  |  |  |  | 0 |
| AM04256 | GB11636 | Ric | |  |  |  |  |  |  | 0 |
| AM04269 | GB11649 | CG33080 | |  |  |  |  |  |  | 0 |
| AM04283 | GB11663 | Nmt | |  |  |  |  |  |  | 0 |
| AM04289 | GB11669 | CG14483 | |  |  |  |  |  |  | 0 |
| AM04309 | GB11689 | babo | |  |  |  |  |  |  | 0 |
| AM04363 | GB11744 | CG12876 | |  |  |  |  |  |  | 0 |
| AM04423 | GB11804 | MED15 | |  |  |  |  |  |  | 0 |
| AM04427 | GB11808 | CG6928 | |  |  |  |  |  |  | 0 |
| AM04460 | GB11841 | Eaat2 | |  |  |  |  |  |  | 0 |
| AM04461 | GB11842 | CG13293 | |  |  |  |  |  |  | 0 |
| AM04467 | GB11848 | mab-2 | |  |  |  |  |  |  | 0 |
| AM04469 | GB11850 | mRpL20 | |  |  |  |  |  |  | 0 |
| AM04470 | GB11851 | CG6729 | |  |  |  |  |  |  | 0 |
| AM04481 | GB11862 | CG6770 | |  |  |  |  |  |  | 0 |
| AM04500 | GB11882 | CG8300 | |  |  |  |  |  |  | 0 |
| AM04541 | GB11924 | pho | |  |  |  |  |  |  | 0 |
| AM04584 | GB11967 | CG4996 | |  |  |  |  |  |  | 0 |
| AM04599 | GB11982 | CG10268 | |  |  |  |  |  |  | 0 |
| AM04607 | GB11990 | CG14446 | |  |  |  |  |  |  | 0 |
| AM04610 | GB11993 | CG10927 | |  |  |  |  |  |  | 0 |
| AM04618 | GB12001 | CG31666 | |  |  |  |  |  |  | 0 |
| AM04620 | GB12003 | CG6254 | |  |  |  |  |  |  | 0 |
| AM04626 | GB12009 | wgn | |  |  |  |  |  |  | 0 |
| AM04676 | GB12059 | Arc-p20 | |  |  |  |  |  |  | 0 |
| AM04707 | GB12090 | CG3321 | |  |  |  |  |  |  | 0 |
| AM04711 | GB12094 | lola | |  |  |  |  |  |  | 0 |
| AM04712 | GB12094 | lola | |  |  |  |  |  |  | 0 |
| AM04713 | GB12094 | lola | |  |  |  |  |  |  | 0 |
| AM04714 | GB12094 | lola | |  |  |  |  |  |  | 0 |
| AM04739 | GB12118 | CG5382 | |  |  |  |  |  |  | 0 |
| AM04830 | GB12210 | CG4090 | |  |  |  |  |  |  | 0 |
| AM04837 | GB12218 | His1:CG33864 | |  |  |  |  |  |  | 0 |
| AM04843 | GB12224 | CG4293 | |  |  |  |  |  |  | 0 |
| AM04906 | GB12287 | bib | |  |  |  |  |  |  | 0 |
| AM04943 | GB12324 | Rnp4F | |  |  |  |  |  |  | 0 |
| AM04953 | GB12334 | CG31064 | |  |  |  |  |  |  | 0 |
| AM04954 | GB12335 | CG5694 | |  |  |  |  |  |  | 0 |
| AM04958 | GB12339 | nmo | |  |  |  |  |  |  | 0 |
| AM04964 | GB12346 | CG16947 | |  |  |  |  |  |  | 0 |
| AM04975 | GB12357 | ssh | |  |  |  |  |  |  | 0 |
| AM04976 | GB12358 | CG9344 | |  |  |  |  |  |  | 0 |
| AM04978 | GB12360 | Pdk | |  |  |  |  |  |  | 0 |
| AM04979 | GB12361 | RpL24 | |  |  |  |  |  |  | 0 |
| AM04987 | GB12369 | dpr9 | |  |  |  |  |  |  | 0 |
| AM04989 | GB12371 | Mgstl | |  |  |  |  |  |  | 0 |
| AM05014 | GB12396 | Dip-C | |  |  |  |  |  |  | 0 |
| AM05036 | GB12419 | Mcm3 | |  |  |  |  |  |  | 0 |
| AM05089 | GB12472 | crol | |  |  |  |  |  |  | 0 |
| AM05111 | GB12494 | CG15269 | |  |  |  |  |  |  | 0 |
| AM05145 | GB12529 | CG1753 | |  |  |  |  |  |  | 0 |
| AM05183 | GB12567 | bgm | |  |  |  |  |  |  | 0 |
| AM05196 | GB12580 | CG15720 | |  |  |  |  |  |  | 0 |
| AM05202 | GB12586 | Pdi | |  |  |  |  |  |  | 0 |
| AM05319 | GB12704 | CG2698 | |  |  |  |  |  |  | 0 |
| AM05338 | GB12723 | CG3071 | |  |  |  |  |  |  | 0 |
| AM05351 | GB12736 | CG5913 | |  |  |  |  |  |  | 0 |
| AM05356 | GB12741 | Aldh | |  |  |  |  |  |  | 0 |
| AM05361 | GB12746 | CG32133 | |  |  |  |  |  |  | 0 |
| AM05394 | GB12779 | vih | |  |  |  |  |  |  | 0 |
| AM05395 | GB12780 | CG3587 | |  |  |  |  |  |  | 0 |
| AM05426 | GB12811 | CG2150 | |  |  |  |  |  |  | 0 |
| AM05446 | GB12832 | CG7974 | |  |  |  |  |  |  | 0 |
| AM05454 | GB12840 | sqd | |  |  |  |  |  |  | 0 |
| AM05480 | GB12866 | Arf79F | |  |  |  |  |  |  | 0 |
| AM05497 | GB12884 | CG17090 | |  |  |  |  |  |  | 0 |
| AM05524 | GB12911 | Tsp3A | |  |  |  |  |  |  | 0 |
| AM05534 | GB12922 | His2B:CG17949 | |  |  |  |  |  |  | 0 |
| AM00757R | GB12929 | para | |  |  |  |  |  |  | 0 |
| AM05602 | GB12991 | His2Av | |  |  |  |  |  |  | 0 |
| AM05608 | GB12997 | CG5288 | |  |  |  |  |  |  | 0 |
| AM05613 | GB13002 | CG3781 | |  |  |  |  |  |  | 0 |
| AM05627 | GB13016 | GABA-B-R3 | |  |  |  |  |  |  | 0 |
| AM05655 | GB13045 | CG4685 | |  |  |  |  |  |  | 0 |
| AM05658 | GB13048 | CG4159 | |  |  |  |  |  |  | 0 |
| AM05710 | GB13102 | CG8506 | |  |  |  |  |  |  | 0 |
| AM05726 | GB13118 | Ssrp | |  |  |  |  |  |  | 0 |
| AM05741 | GB13133 | Ugt35b | |  |  |  |  |  |  | 0 |
| AM05827 | GB13219 | asparagine-synthetase | |  |  |  |  |  |  | 0 |
| AM05893 | GB13286 | CG10214 | |  |  |  |  |  |  | 0 |
| AM05897 | GB13290 | tnc | |  |  |  |  |  |  | 0 |
| AM05926 | GB13322 | CG8314 | |  |  |  |  |  |  | 0 |
| AM05929 | GB13325 | PebIII | |  |  |  |  |  |  | 0 |
| AM05931 | GB13327 | pbl | |  |  |  |  |  |  | 0 |
| AM05934 | GB13330 | Nep4 | |  |  |  |  |  |  | 0 |
| AM05950 | GB13346 | RhoGAP92B | |  |  |  |  |  |  | 0 |
| AM05971 | GB13368 | CG9914 | |  |  |  |  |  |  | 0 |
| AM05998 | GB13395 | CG10221 | |  |  |  |  |  |  | 0 |
| AM05999 | GB13396 | CG15890 | |  |  |  |  |  |  | 0 |
| AM06028 | GB13424 | RnrS | |  |  |  |  |  |  | 0 |
| AM06051 | GB13447 | CG31224 | |  |  |  |  |  |  | 0 |
| AM00351 | GB13473 | CG15021 | |  |  |  |  |  |  | 0 |
| AM00352 | GB13473 | CG15021 | |  |  |  |  |  |  | 0 |
| AM00353 | GB13473 | CG15021 | |  |  |  |  |  |  | 0 |
| AM00358 | GB13473-RB | CG15021 | |  |  |  |  |  |  | 0 |
| AM06086 | GB13483 | CG31550 | |  |  |  |  |  |  | 0 |
| AM06095 | GB13493 | Rh5 | |  |  |  |  |  |  | 0 |
| AM06098 | GB13496 | Rgk2 | |  |  |  |  |  |  | 0 |
| AM06099 | GB13497 | tacc | |  |  |  |  |  |  | 0 |
| AM06103 | GB13501 | CG4797 | |  |  |  |  |  |  | 0 |
| AM06117 | GB13515 | CG4538 | |  |  |  |  |  |  | 0 |
| AM06135 | GB13534 | CG4644 | |  |  |  |  |  |  | 0 |
| AM06152 | GB13551 | CG12851 | |  |  |  |  |  |  | 0 |
| AM06176 | GB13575 | torp4a | |  |  |  |  |  |  | 0 |
| AM06203 | GB13603 | CG7628 | |  |  |  |  |  |  | 0 |
| AM06224 | GB13625 | CG1090 | |  |  |  |  |  |  | 0 |
| AM06255 | GB13657 | CG5794 | |  |  |  |  |  |  | 0 |
| AM06267 | GB13669 | CG15040 | |  |  |  |  |  |  | 0 |
| AM06280 | GB13682 | CG6860 | |  |  |  |  |  |  | 0 |
| AM06298 | GB13700 | Mhc | |  |  |  |  |  |  | 0 |
| AM06300 | GB13702 | CG4610 | |  |  |  |  |  |  | 0 |
| AM06315 | GB13717 | CG13503 | |  |  |  |  |  |  | 0 |
| AM06342 | GB13743 | Tom70 | |  |  |  |  |  |  | 0 |
| AM06363 | GB13764 | CG15658 | |  |  |  |  |  |  | 0 |
| AM06368 | GB13769 | mRpS22 | |  |  |  |  |  |  | 0 |
| AM06388 | GB13789 | yellow-h | |  |  |  |  |  |  | 0 |
| AM06400 | GB13801 | CG14299 | |  |  |  |  |  |  | 0 |
| AM06422 | GB13823 | CG11360 | |  |  |  |  |  |  | 0 |
| AM06436 | GB13837 | CG12252 | |  |  |  |  |  |  | 0 |
| AM06452 | GB13853 | Dys | |  |  |  |  |  |  | 0 |
| AM06507 | GB13909 | CG12017 | |  |  |  |  |  |  | 0 |
| AM06516 | GB13918 | CG17108 | |  |  |  |  |  |  | 0 |
| AM06524 | GB13926 | argos | |  |  |  |  |  |  | 0 |
| AM06650 | GB14051 | CG9547 | |  |  |  |  |  |  | 0 |
| AM06656 | GB14057 | CG13690 | |  |  |  |  |  |  | 0 |
| AM06676 | GB14074 | CG11986 | |  |  |  |  |  |  | 0 |
| AM06762 | GB14162 | CG4756 | |  |  |  |  |  |  | 0 |
| AM06807 | GB14208 | CG32627 | |  |  |  |  |  |  | 0 |
| AM06852 | GB14253 | CG7275 | |  |  |  |  |  |  | 0 |
| AM06872 | GB14273 | neur | |  |  |  |  |  |  | 0 |
| AM06892 | GB14292 | dpr8 | |  |  |  |  |  |  | 0 |
| AM06941 | GB14342 | CG3021 | |  |  |  |  |  |  | 0 |
| AM06954 | GB14356 | Gint3 | |  |  |  |  |  |  | 0 |
| AM06980 | GB14382 | Nrx-IV | |  |  |  |  |  |  | 0 |
| AM07015 | GB14417 | CG14482 | |  |  |  |  |  |  | 0 |
| AM07086 | GB14488 | kat80 | |  |  |  |  |  |  | 0 |
| AM07128 | GB14529 | Ptp61F | |  |  |  |  |  |  | 0 |
| AM07154 | GB14556 | CG14950 | |  |  |  |  |  |  | 0 |
| AM07169 | GB14572 | CG15211 | |  |  |  |  |  |  | 0 |
| AM07186 | GB14589 | CG14515 | |  |  |  |  |  |  | 0 |
| AM07189 | GB14592 | CG8503 | |  |  |  |  |  |  | 0 |
| AM07274 | GB14677 | CG11089 | |  |  |  |  |  |  | 0 |
| AM07281 | GB14684 | CG31522 | |  |  |  |  |  |  | 0 |
| AM07292 | GB14695 | Rph | |  |  |  |  |  |  | 0 |
| AM07299 | GB14702 | CG7156 | |  |  |  |  |  |  | 0 |
| AM07346 | GB14749 | NPC1 | |  |  |  |  |  |  | 0 |
| AM07360 | GB14765 | CG16833 | |  |  |  |  |  |  | 0 |
| AM07389 | GB14794 | sds22 | |  |  |  |  |  |  | 0 |
| AM07409 | GB14814 | CG18809 | |  |  |  |  |  |  | 0 |
| AM07411 | GB14816 | CG7786 | |  |  |  |  |  |  | 0 |
| AM07424 | GB14829 | CG4452 | |  |  |  |  |  |  | 0 |
| AM12801 | GB14865 | Tsp42Ea | |  |  |  |  |  |  | 0 |
| AM07474 | GB14880 | CG17683 | |  |  |  |  |  |  | 0 |
| AM07482 | GB14888 | yellow-h | |  |  |  |  |  |  | 0 |
| AM07488 | GB14894 | mRpL27 | |  |  |  |  |  |  | 0 |
| AM07512 | GB14921 | Suv4-20 | |  |  |  |  |  |  | 0 |
| AM07541 | GB14950 | kel | |  |  |  |  |  |  | 0 |
| AM07546 | GB14955 | adat | |  |  |  |  |  |  | 0 |
| AM07563 | GB14971 | Lerp | |  |  |  |  |  |  | 0 |
| AM07583 | GB14992 | CG30197 | |  |  |  |  |  |  | 0 |
| AM07607 | GB15016 | Hsc70-3 | |  |  |  |  |  |  | 0 |
| AM07609 | GB15018 | Hml | |  |  |  |  |  |  | 0 |
| AM07624 | GB15033 | CG32705 | |  |  |  |  |  |  | 0 |
| AM07677 | GB15086 | SC35 | |  |  |  |  |  |  | 0 |
| AM07682 | GB15091 | bab1 | |  |  |  |  |  |  | 0 |
| AM07683 | GB15092 | Tom20 | |  |  |  |  |  |  | 0 |
| AM07707 | GB15117 | Cha | |  |  |  |  |  |  | 0 |
| AM07753 | GB15163 | CG17323 | |  |  |  |  |  |  | 0 |
| AM07826 | GB15237 | CG34139 | |  |  |  |  |  |  | 0 |
| AM07851 | GB15262 | CG34109 | |  |  |  |  |  |  | 0 |
| AM07868 | GB15279 | CG15040 | |  |  |  |  |  |  | 0 |
| AM07873 | GB15283 | CG10077 | |  |  |  |  |  |  | 0 |
| AM07875 | GB15285 | CG30497 | |  |  |  |  |  |  | 0 |
| AM07912 | GB15324 | CG8027 | |  |  |  |  |  |  | 0 |
| AM07921 | GB15333 | Adk3 | |  |  |  |  |  |  | 0 |
| AM07931 | GB15343 | CG9922 | |  |  |  |  |  |  | 0 |
| AM07946 | GB15358 | CG15785 | |  |  |  |  |  |  | 0 |
| AM07969 | GB15381 | CG32677 | |  |  |  |  |  |  | 0 |
| AM07978 | GB15390 | CG15040 | |  |  |  |  |  |  | 0 |
| AM07979 | GB15391 | CG5613 | |  |  |  |  |  |  | 0 |
| AM08041 | GB15454 | CG14995 | |  |  |  |  |  |  | 0 |
| AM08047 | GB15460 | Obp56d | |  |  |  |  |  |  | 0 |
| AM08140 | GB15555 | CG6522 | |  |  |  |  |  |  | 0 |
| AM08191 | GB15607 | CG15029 | |  |  |  |  |  |  | 0 |
| AM08222 | GB15638 | wus | |  |  |  |  |  |  | 0 |
| AM08247 | GB15664 | kst | |  |  |  |  |  |  | 0 |
| AM08255 | GB15672 | CG10960 | |  |  |  |  |  |  | 0 |
| AM08292 | GB15709 | Snap | |  |  |  |  |  |  | 0 |
| AM08306 | GB15723 | Rrp45 | |  |  |  |  |  |  | 0 |
| AM08327 | GB15745 | CG5618 | |  |  |  |  |  |  | 0 |
| AM08345 | GB15763 | CG13398 | |  |  |  |  |  |  | 0 |
| AM02021 | GB15792 | B52 | |  |  |  |  |  |  | 0 |
| AM08374 | GB15792 | B52 | |  |  |  |  |  |  | 0 |
| AM08433 | GB15851 | CG32447 | |  |  |  |  |  |  | 0 |
| AM00244 | GB15899 | Or82a | |  |  |  |  |  |  | 0 |
| AM08483 | GB15902 | ari-2 | |  |  |  |  |  |  | 0 |
| AM08490 | GB15909 | ewg | |  |  |  |  |  |  | 0 |
| AM08555 | GB15974 | Gug | |  |  |  |  |  |  | 0 |
| AM08562 | GB15981 | trr | |  |  |  |  |  |  | 0 |
| AM08572 | GB15990 | CG6783 | |  |  |  |  |  |  | 0 |
| AM08625 | GB16044 | CG14562 | |  |  |  |  |  |  | 0 |
| AM08652 | GB16071 | Abi | |  |  |  |  |  |  | 0 |
| AM08796 | GB16216 | CG7565 | |  |  |  |  |  |  | 0 |
| AM08843 | GB16263 | Amph | |  |  |  |  |  |  | 0 |
| AM08883 | GB16303 | mRpS18A | |  |  |  |  |  |  | 0 |
| AM08895 | GB16315 | Ssl1 | |  |  |  |  |  |  | 0 |
| AM08926 | GB16346 | ras | |  |  |  |  |  |  | 0 |
| AM08934 | GB16354 | CG12384 | |  |  |  |  |  |  | 0 |
| AM08944 | GB16363 | CG7323 | |  |  |  |  |  |  | 0 |
| AM08952 | GB16371 | CG7712 | |  |  |  |  |  |  | 0 |
| AM08960 | GB16379 | twin | |  |  |  |  |  |  | 0 |
| AM08981 | GB16400 | abs | |  |  |  |  |  |  | 0 |
| AM08987 | GB16406 | CG8289 | |  |  |  |  |  |  | 0 |
| AM08990 | GB16409 | sec5 | |  |  |  |  |  |  | 0 |
| AM08992 | GB16413 | CG10470 | |  |  |  |  |  |  | 0 |
| AM09002 | GB16423 | Fs(2)Ket | |  |  |  |  |  |  | 0 |
| AM09018 | GB16439 | CG17912 | |  |  |  |  |  |  | 0 |
| AM09028 | GB16449 | Pi4KIIalpha | |  |  |  |  |  |  | 0 |
| AM09038 | GB16459 | yellow-h | |  |  |  |  |  |  | 0 |
| AM09116 | GB16537 | CG10077 | |  |  |  |  |  |  | 0 |
| AM09121 | GB16542 | Sur-8 | |  |  |  |  |  |  | 0 |
| AM09197 | GB16619 | Nep2 | |  |  |  |  |  |  | 0 |
| AM09211 | GB16633 | okr | |  |  |  |  |  |  | 0 |
| AM09264 | GB16685 | CG5880 | |  |  |  |  |  |  | 0 |
| AM00333 | GB16692 | CG12045 | |  |  |  |  |  |  | 0 |
| AM09271 | GB16692 | CG12045 | |  |  |  |  |  |  | 0 |
| AM09275 | GB16696 | CG14325 | |  |  |  |  |  |  | 0 |
| AM09388 | GB16811 | gus | |  |  |  |  |  |  | 0 |
| AM09504 | GB16927 | nahoda | |  |  |  |  |  |  | 0 |
| AM09514 | GB16937 | MAPk-Ak2 | |  |  |  |  |  |  | 0 |
| AM09533 | GB16956 | Dhod | |  |  |  |  |  |  | 0 |
| AM09557 | GB16980 | CG31937 | |  |  |  |  |  |  | 0 |
| AM09567 | GB16990 | cpsf | |  |  |  |  |  |  | 0 |
| AM12811 | GB16999 | Lgr3 | |  |  |  |  |  |  | 0 |
| AM09601 | GB17025 | Frq2 | |  |  |  |  |  |  | 0 |
| AM09642 | GB17066 | CG3608 | |  |  |  |  |  |  | 0 |
| AM09645 | GB17069 | PP2A-B' | |  |  |  |  |  |  | 0 |
| AM09659 | GB17084 | Fas3 | |  |  |  |  |  |  | 0 |
| AM09663 | GB17088 | mRpS30 | |  |  |  |  |  |  | 0 |
| AM09692 | GB17117 | Gpi1 | |  |  |  |  |  |  | 0 |
| AM09693 | GB17118 | CG18619 | |  |  |  |  |  |  | 0 |
| AM09698 | GB17123 | CG11454 | |  |  |  |  |  |  | 0 |
| AM09708 | GB17133 | nAcRalpha-96Aa | |  |  |  |  |  |  | 0 |
| AM09733 | GB17158 | CG8446 | |  |  |  |  |  |  | 0 |
| AM09755 | GB17180 | CG15483 | |  |  |  |  |  |  | 0 |
| AM09784 | GB17209 | SH3PX1 | |  |  |  |  |  |  | 0 |
| AM09789 | GB17214 | Fkbp13 | |  |  |  |  |  |  | 0 |
| AM09829 | GB17254 | nAcRalpha-30D | |  |  |  |  |  |  | 0 |
| AM09862 | GB17287 | Rph | |  |  |  |  |  |  | 0 |
| AM09876 | GB17301 | CG31140 | |  |  |  |  |  |  | 0 |
| AM09888 | GB17313 | CG12121 | |  |  |  |  |  |  | 0 |
| AM09918 | GB17343 | ttv | |  |  |  |  |  |  | 0 |
| AM09950 | GB17377 | CG2604 | |  |  |  |  |  |  | 0 |
| AM09977 | GB17404 | kermit | |  |  |  |  |  |  | 0 |
| AM09981 | GB17409 | CG6981 | |  |  |  |  |  |  | 0 |
| AM10041 | GB17470 | CG9418 | |  |  |  |  |  |  | 0 |
| AM10119 | GB17548 | CG3603 | |  |  |  |  |  |  | 0 |
| AM10123 | GB17552 | Ercc1 | |  |  |  |  |  |  | 0 |
| AM10124 | GB17553 | CG4858 | |  |  |  |  |  |  | 0 |
| AM10135 | GB17563 | smg | |  |  |  |  |  |  | 0 |
| AM10137 | GB17565 | CG31064 | |  |  |  |  |  |  | 0 |
| AM10148 | GB17576 | CG33017 | |  |  |  |  |  |  | 0 |
| AM10161 | GB17590 | CG4587 | |  |  |  |  |  |  | 0 |
| AM10162 | GB17591 | RpII215 | |  |  |  |  |  |  | 0 |
| AM10178 | GB17607 | Ogg1 | |  |  |  |  |  |  | 0 |
| AM10193 | GB17619 | deltaCOP | |  |  |  |  |  |  | 0 |
| AM10238 | GB17664 | CG4778 | |  |  |  |  |  |  | 0 |
| AM10252 | GB17678 | CG4133 | |  |  |  |  |  |  | 0 |
| AM10260 | GB17687 | dos | |  |  |  |  |  |  | 0 |
| AM10281 | GB17708 | CG3980 | |  |  |  |  |  |  | 0 |
| AM10298 | GB17725 | CG5222 | |  |  |  |  |  |  | 0 |
| AM10313 | GB17740 | CG8202 | |  |  |  |  |  |  | 0 |
| AM10351 | GB17778 | CG11107 | |  |  |  |  |  |  | 0 |
| AM00354 | GB17782 | CG15021 | |  |  |  |  |  |  | 0 |
| AM00355 | GB17782 | CG15021 | |  |  |  |  |  |  | 0 |
| AM00356 | GB17782 | CG15021 | |  |  |  |  |  |  | 0 |
| AM00357 | GB17782 | CG15021 | |  |  |  |  |  |  | 0 |
| AM00360 | GB17782 | CG15021 | |  |  |  |  |  |  | 0 |
| AM10433 | GB17862 | FucT6 | |  |  |  |  |  |  | 0 |
| AM10438 | GB17867 | CG32698 | |  |  |  |  |  |  | 0 |
| AM10446 | GB17875 | PebIII | |  |  |  |  |  |  | 0 |
| AM10453 | GB17882 | CG34126 | |  |  |  |  |  |  | 0 |
| AM10471 | GB17900 | CG6128 | |  |  |  |  |  |  | 0 |
| AM10494 | GB17923 | CG31108 | |  |  |  |  |  |  | 0 |
| AM10504 | GB17931 | CG5065 | |  |  |  |  |  |  | 0 |
| AM10518 | GB17945 | ct | |  |  |  |  |  |  | 0 |
| AM10529 | GB17956 | CG33525 | |  |  |  |  |  |  | 0 |
| AM10555 | GB17982 | CG32372 | |  |  |  |  |  |  | 0 |
| AM10585 | GB18012 | CG11095 | |  |  |  |  |  |  | 0 |
| AM10606 | GB18033 | dpld | |  |  |  |  |  |  | 0 |
| AM10679 | GB18109 | CG6084 | |  |  |  |  |  |  | 0 |
| AM10787 | GB18221 | CG10306 | |  |  |  |  |  |  | 0 |
| AM10808 | GB18242 | Hmgcr | |  |  |  |  |  |  | 0 |
| AM10849 | GB18284 | alpha-Man-I | |  |  |  |  |  |  | 0 |
| AM10854 | GB18289 | CG15040 | |  |  |  |  |  |  | 0 |
| AM10939 | GB18375 | sds22 | |  |  |  |  |  |  | 0 |
| AM10977 | GB18414 | Ace | |  |  |  |  |  |  | 0 |
| AM10982 | GB18419 | CG5319 | |  |  |  |  |  |  | 0 |
| AM10998 | GB18435 | p130CAS | |  |  |  |  |  |  | 0 |
| AM11024 | GB18461 | CG7082 | |  |  |  |  |  |  | 0 |
| AM11076 | GB18514 | CG14869 | |  |  |  |  |  |  | 0 |
| AM11092 | GB18531 | Cks85A | |  |  |  |  |  |  | 0 |
| AM11154 | GB18593 | CG6293 | |  |  |  |  |  |  | 0 |
| AM11228 | GB18667 | CG1440 | |  |  |  |  |  |  | 0 |
| AM11315 | GB18755 | gammaTub23C | |  |  |  |  |  |  | 0 |
| AM11318 | GB18758 | Grip128 | |  |  |  |  |  |  | 0 |
| AM11325 | GB18765 | CG4972 | |  |  |  |  |  |  | 0 |
| AM11353 | GB18794 | CG18600 | |  |  |  |  |  |  | 0 |
| AM11376 | GB18818 | Tace | |  |  |  |  |  |  | 0 |
| AM11416 | GB18859 | CG8291 | |  |  |  |  |  |  | 0 |
| AM11418 | GB18861 | CG10743 | |  |  |  |  |  |  | 0 |
| AM11459 | GB18902 | Nca | |  |  |  |  |  |  | 0 |
| AM11475 | GB18918 | zfh1 | |  |  |  |  |  |  | 0 |
| AM11495 | GB18938 | CG9281 | |  |  |  |  |  |  | 0 |
| AM11498 | GB18942 | Nmdar2 | |  |  |  |  |  |  | 0 |
| AM11527 | GB18971 | l(1)G0232 | |  |  |  |  |  |  | 0 |
| AM11529 | GB18973 | ari-2 | |  |  |  |  |  |  | 0 |
| AM11537 | GB18981 | Med | |  |  |  |  |  |  | 0 |
| AM11541 | GB18985 | CG7261 | |  |  |  |  |  |  | 0 |
| AM11573 | GB19017 | CG14934 | |  |  |  |  |  |  | 0 |
| AM11586 | GB19031 | Hand | |  |  |  |  |  |  | 0 |
| AM11602 | GB19047 | CG4098 | |  |  |  |  |  |  | 0 |
| AM11608 | GB19053 | CG5706 | |  |  |  |  |  |  | 0 |
| AM11630 | GB19075 | CG3153 | |  |  |  |  |  |  | 0 |
| AM11637 | GB19082 | sta | |  |  |  |  |  |  | 0 |
| AM11638 | GB19083 | CG5155 | |  |  |  |  |  |  | 0 |
| AM11656 | GB19102 | CG11247 | |  |  |  |  |  |  | 0 |
| AM11658 | GB19104 | Or13a | |  |  |  |  |  |  | 0 |
| AM11705 | GB19151 | Mmp1 | |  |  |  |  |  |  | 0 |
| AM11791 | GB19237 | CG2165 | |  |  |  |  |  |  | 0 |
| AM11792 | GB19238 | CG9796 | |  |  |  |  |  |  | 0 |
| AM11812 | GB19258 | Bap170 | |  |  |  |  |  |  | 0 |
| AM11838 | GB19284 | mxc | |  |  |  |  |  |  | 0 |
| AM11865 | GB19312 | CG1115 | |  |  |  |  |  |  | 0 |
| AM11901 | GB19348 | CLIP-190 | |  |  |  |  |  |  | 0 |
| AM11902 | GB19349 | rho-5 | |  |  |  |  |  |  | 0 |
| AM11926 | GB19373 | DppIII | |  |  |  |  |  |  | 0 |
| AM11934 | GB19381 | CG12765 | |  |  |  |  |  |  | 0 |
| AM11951 | GB19398 | CG15925 | |  |  |  |  |  |  | 0 |
| AM02626 | GB19418 | Gld | |  |  |  |  |  |  | 0 |
| AM11970 | GB19418 | Gld | |  |  |  |  |  |  | 0 |
| AM11972 | GB19420 | ninaC | |  |  |  |  |  |  | 0 |
| AM12008 | GB19457 | emc | |  |  |  |  |  |  | 0 |
| AM12011 | GB19460 | Ald | |  |  |  |  |  |  | 0 |
| AM12012 | GB19460 | Ald | |  |  |  |  |  |  | 0 |
| AM12043 | GB19491 | fry | |  |  |  |  |  |  | 0 |
| AM12071 | GB19519 | CG3257 | |  |  |  |  |  |  | 0 |
| AM12100 | GB19548 | kkv | |  |  |  |  |  |  | 0 |
| AM12149 | GB19596 | brp | |  |  |  |  |  |  | 0 |
| AM12155 | GB19603 | CG6656 | |  |  |  |  |  |  | 0 |
| AM12156 | GB19604 | Eip75B | |  |  |  |  |  |  | 0 |
| AM12159 | GB19607 | Smox | |  |  |  |  |  |  | 0 |
| AM12205 | GB19656 | CG2924 | |  |  |  |  |  |  | 0 |
| AM12207 | GB19658 | CG7382 | |  |  |  |  |  |  | 0 |
| AM12231 | GB19683-RB | emp | |  |  |  |  |  |  | 0 |
| AM12288 | GB19741 | eIF2B-delta | |  |  |  |  |  |  | 0 |
| AM12312 | GB19765 | Ca-alpha1T | |  |  |  |  |  |  | 0 |
| AM12318 | GB19771 | CG8830 | |  |  |  |  |  |  | 0 |
| AM12358 | GB19811 | CG13618 | |  |  |  |  |  |  | 0 |
| AM12382 | GB19835 | CG13282 | |  |  |  |  |  |  | 0 |
| AM12390 | GB19843 | CG7402 | |  |  |  |  |  |  | 0 |
| AM12393 | GB19846 | CG5255 | |  |  |  |  |  |  | 0 |
| AM12400 | GB19853 | Bgb | |  |  |  |  |  |  | 0 |
| AM12401 | GB19854 | CG7280 | |  |  |  |  |  |  | 0 |
| AM12403 | GB19856 | CG31265 | |  |  |  |  |  |  | 0 |
| AM12405 | GB19858 | beat-VI | |  |  |  |  |  |  | 0 |
| AM12422 | GB19875 | CG5493 | |  |  |  |  |  |  | 0 |
| AM12457 | GB19910 | CG32158 | |  |  |  |  |  |  | 0 |
| AM12492 | GB19945 | trn | |  |  |  |  |  |  | 0 |
| AM12518 | GB19971 | gl | |  |  |  |  |  |  | 0 |
| AM12553 | GB20006 | blue | |  |  |  |  |  |  | 0 |
| AM12558 | GB20011 | CG10103 | |  |  |  |  |  |  | 0 |
| AM12562 | GB20015 | CG6305 | |  |  |  |  |  |  | 0 |
| AM12583 | GB20036 | syt | |  |  |  |  |  |  | 0 |
| AM12597 | GB20050 | CG5515 | |  |  |  |  |  |  | 0 |
| AM12608 | GB20060 | CG11498 | |  |  |  |  |  |  | 0 |
| AM12616 | GB20068 | CG10721 | |  |  |  |  |  |  | 0 |
| AM12637 | GB20089 | CG32654 | |  |  |  |  |  |  | 0 |
| AM12656 | GB20109 | CG1951 | |  |  |  |  |  |  | 0 |
| AM12672 | GB20125 | CG11980 | |  |  |  |  |  |  | 0 |
| AM03764 | GB30057 | CG33259 | |  |  |  |  |  |  | 0 |
| AM09654 | GB30070_2 | mib1 | |  |  |  |  |  |  | 0 |
| AM09209 | GB30075 | CG7896 | |  |  |  |  |  |  | 0 |
| AM03628 | GB30096 | simj | |  |  |  |  |  |  | 0 |
| AM12768 | GB30101 | plexA | |  |  |  |  |  |  | 0 |
| AM02897 | GB30104 | CG6416 | |  |  |  |  |  |  | 0 |
| AM09534 | GB30112 | mfr | |  |  |  |  |  |  | 0 |
| AM04832 | GB30215 | kay | |  |  |  |  |  |  | 0 |
| AM04138 | GB30227_2 | Cat | |  |  |  |  |  |  | 0 |
| AM11945 | GB30228 | Def | |  |  |  |  |  |  | 0 |
| AM06645 | GB30234 | Ets65A | |  |  |  |  |  |  | 0 |
| AM12851R | GB30268 | GstS1 | |  |  |  |  |  |  | 0 |
| AM12756 | GB30320 | Gr28b | |  |  |  |  |  |  | 0 |
| AM11450 | GB30325 | XNP | |  |  |  |  |  |  | 0 |
| AM11412 | GB30326 | CG12014 | |  |  |  |  |  |  | 0 |
| AM12746 | GB30328 | CG1738 | |  |  |  |  |  |  | 0 |
| AM08998 | GB30339 | HLH106 | |  |  |  |  |  |  | 0 |
| AM12781 | GB30362 | Lsp2 | |  |  |  |  |  |  | 0 |
| AM12614 | GB30368 | glo | |  |  |  |  |  |  | 0 |
| AM11293 | GB30378 | snk | |  |  |  |  |  |  | 0 |
| AM09052 | GB30393 | RpL11 | |  |  |  |  |  |  | 0 |
| AM04880 | GB30566 | CG14692 | |  |  |  |  |  |  | 0 |
| AM08642 | GB30579_2 | Gcn2 | |  |  |  |  |  |  | 0 |
| AM00021 |  |  | |  |  |  |  |  |  | 0 |
| AM00413R |  |  | |  |  |  |  |  |  | 0 |
| AM00705 |  |  | |  |  |  |  |  |  | 0 |
| AM01004 |  |  | |  |  |  |  |  |  | 0 |
| AM01196 |  |  | |  |  |  |  |  |  | 0 |
| AM01249 |  |  | |  |  |  |  |  |  | 0 |
| AM01368 |  |  | |  |  |  |  |  |  | 0 |
| AM01403 |  |  | |  |  |  |  |  |  | 0 |
| AM01508 |  |  | |  |  |  |  |  |  | 0 |
| AM01669 |  |  | |  |  |  |  |  |  | 0 |
| AM01713 |  |  | |  |  |  |  |  |  | 0 |
| AM01766 |  |  | |  |  |  |  |  |  | 0 |
| AM01776 |  |  | |  |  |  |  |  |  | 0 |
| AM01798 |  |  | |  |  |  |  |  |  | 0 |
| AM01920 |  | kay | |  |  |  |  |  |  | 0 |
| AM01974 |  |  | |  |  |  |  |  |  | 0 |
| AM01979 |  |  | |  |  |  |  |  |  | 0 |
| AM02115 |  |  | |  |  |  |  |  |  | 0 |
| AM02132 |  | Eip93F | |  |  |  |  |  |  | 0 |
| AM02315 |  |  | |  |  |  |  |  |  | 0 |
| AM02398 |  |  | |  |  |  |  |  |  | 0 |
| AM02418 |  |  | |  |  |  |  |  |  | 0 |
| AM02635 |  |  | |  |  |  |  |  |  | 0 |
| AM03132 |  |  | |  |  |  |  |  |  | 0 |
| AM03211 |  |  | |  |  |  |  |  |  | 0 |
| AM03485 |  |  | |  |  |  |  |  |  | 0 |
| AM03624 |  |  | |  |  |  |  |  |  | 0 |
| AM05067 |  |  | |  |  |  |  |  |  | 0 |
| AM06462 |  |  | |  |  |  |  |  |  | 0 |
| AM07863 |  |  | |  |  |  |  |  |  | 0 |
| AM09945 |  |  | |  |  |  |  |  |  | 0 |
| AM10302 |  |  | |  |  |  |  |  |  | 0 |
| AM10618 |  |  | |  |  |  |  |  |  | 0 |
| AM11030 |  |  | |  |  |  |  |  |  | 0 |
| AM12843R |  |  | |  |  |  |  |  |  | 0 |
| AM00021R |  |  | |  |  |  |  |  |  | 0 |
| AM00045 |  |  | |  |  |  |  |  |  | 0 |
| AM00070 |  |  | |  |  |  |  |  |  | 0 |
| AM00073 |  |  | |  |  |  |  |  |  | 0 |
| AM00109 |  |  | |  |  |  |  |  |  | 0 |
| AM00148 |  |  | |  |  |  |  |  |  | 0 |
| AM00159 |  |  | |  |  |  |  |  |  | 0 |
| AM00359 |  |  | |  |  |  |  |  |  | 0 |
| AM00366 |  |  | |  |  |  |  |  |  | 0 |
| AM00378 |  |  | |  |  |  |  |  |  | 0 |
| AM00391R |  |  | |  |  |  |  |  |  | 0 |
| AM00408 |  |  | |  |  |  |  |  |  | 0 |
| AM00417R |  |  | |  |  |  |  |  |  | 0 |
| AM00429 |  |  | |  |  |  |  |  |  | 0 |
| AM00429R |  |  | |  |  |  |  |  |  | 0 |
| AM00439R |  |  | |  |  |  |  |  |  | 0 |
| AM00446 |  |  | |  |  |  |  |  |  | 0 |
| AM00459R |  |  | |  |  |  |  |  |  | 0 |
| AM00463R |  |  | |  |  |  |  |  |  | 0 |
| AM00464 |  |  | |  |  |  |  |  |  | 0 |
| AM00464R |  |  | |  |  |  |  |  |  | 0 |
| AM00496 |  |  | |  |  |  |  |  |  | 0 |
| AM00523 |  |  | |  |  |  |  |  |  | 0 |
| AM00529 |  |  | |  |  |  |  |  |  | 0 |
| AM00539R |  |  | |  |  |  |  |  |  | 0 |
| AM00558 |  |  | |  |  |  |  |  |  | 0 |
| AM00597 |  |  | |  |  |  |  |  |  | 0 |
| AM00598 |  |  | |  |  |  |  |  |  | 0 |
| AM00612 |  |  | |  |  |  |  |  |  | 0 |
| AM00626R |  |  | |  |  |  |  |  |  | 0 |
| AM00647R |  |  | |  |  |  |  |  |  | 0 |
| AM00683 |  |  | |  |  |  |  |  |  | 0 |
| AM00717R |  |  | |  |  |  |  |  |  | 0 |
| AM00733 |  |  | |  |  |  |  |  |  | 0 |
| AM00737 |  |  | |  |  |  |  |  |  | 0 |
| AM00749 |  |  | |  |  |  |  |  |  | 0 |
| AM00761R |  |  | |  |  |  |  |  |  | 0 |
| AM00763 |  |  | |  |  |  |  |  |  | 0 |
| AM00787 |  |  | |  |  |  |  |  |  | 0 |
| AM00823 |  |  | |  |  |  |  |  |  | 0 |
| AM00826 |  |  | |  |  |  |  |  |  | 0 |
| AM00830 |  |  | |  |  |  |  |  |  | 0 |
| AM00859 |  |  | |  |  |  |  |  |  | 0 |
| AM00894 |  |  | |  |  |  |  |  |  | 0 |
| AM00904 |  |  | |  |  |  |  |  |  | 0 |
| AM00951 |  |  | |  |  |  |  |  |  | 0 |
| AM00954 |  |  | |  |  |  |  |  |  | 0 |
| AM00969 |  |  | |  |  |  |  |  |  | 0 |
| AM01005 |  |  | |  |  |  |  |  |  | 0 |
| AM01033 |  |  | |  |  |  |  |  |  | 0 |
| AM01037 |  |  | |  |  |  |  |  |  | 0 |
| AM01043 |  |  | |  |  |  |  |  |  | 0 |
| AM01048 |  |  | |  |  |  |  |  |  | 0 |
| AM01053 |  |  | |  |  |  |  |  |  | 0 |
| AM01093 |  |  | |  |  |  |  |  |  | 0 |
| AM01108 |  |  | |  |  |  |  |  |  | 0 |
| AM01112 |  |  | |  |  |  |  |  |  | 0 |
| AM01159 |  |  | |  |  |  |  |  |  | 0 |
| AM01171 |  |  | |  |  |  |  |  |  | 0 |
| AM01177 |  |  | |  |  |  |  |  |  | 0 |
| AM01178 |  |  | |  |  |  |  |  |  | 0 |
| AM01193 |  |  | |  |  |  |  |  |  | 0 |
| AM01226 |  |  | |  |  |  |  |  |  | 0 |
| AM01228 |  |  | |  |  |  |  |  |  | 0 |
| AM01232 |  |  | |  |  |  |  |  |  | 0 |
| AM01252 |  |  | |  |  |  |  |  |  | 0 |
| AM01255 |  |  | |  |  |  |  |  |  | 0 |
| AM01296 |  |  | |  |  |  |  |  |  | 0 |
| AM01304 |  |  | |  |  |  |  |  |  | 0 |
| AM01313 |  |  | |  |  |  |  |  |  | 0 |
| AM01324 |  |  | |  |  |  |  |  |  | 0 |
| AM01334 |  |  | |  |  |  |  |  |  | 0 |
| AM01335 |  |  | |  |  |  |  |  |  | 0 |
| AM01340 |  |  | |  |  |  |  |  |  | 0 |
| AM01365 |  |  | |  |  |  |  |  |  | 0 |
| AM01391 |  |  | |  |  |  |  |  |  | 0 |
| AM01394 |  |  | |  |  |  |  |  |  | 0 |
| AM01416 |  |  | |  |  |  |  |  |  | 0 |
| AM01423 |  |  | |  |  |  |  |  |  | 0 |
| AM01427 |  |  | |  |  |  |  |  |  | 0 |
| AM01447 |  |  | |  |  |  |  |  |  | 0 |
| AM01474 |  |  | |  |  |  |  |  |  | 0 |
| AM01493 |  |  | |  |  |  |  |  |  | 0 |
| AM01519 |  |  | |  |  |  |  |  |  | 0 |
| AM01524 |  |  | |  |  |  |  |  |  | 0 |
| AM01529 |  |  | |  |  |  |  |  |  | 0 |
| AM01542 |  |  | |  |  |  |  |  |  | 0 |
| AM01552 |  |  | |  |  |  |  |  |  | 0 |
| AM01558 |  |  | |  |  |  |  |  |  | 0 |
| AM01596 |  |  | |  |  |  |  |  |  | 0 |
| AM01607 |  |  | |  |  |  |  |  |  | 0 |
| AM01619 |  |  | |  |  |  |  |  |  | 0 |
| AM01631 |  |  | |  |  |  |  |  |  | 0 |
| AM01634 |  |  | |  |  |  |  |  |  | 0 |
| AM01636 |  |  | |  |  |  |  |  |  | 0 |
| AM01646 |  |  | |  |  |  |  |  |  | 0 |
| AM01667 |  |  | |  |  |  |  |  |  | 0 |
| AM01686 |  |  | |  |  |  |  |  |  | 0 |
| AM01722 |  |  | |  |  |  |  |  |  | 0 |
| AM01735 |  |  | |  |  |  |  |  |  | 0 |
| AM01737 |  |  | |  |  |  |  |  |  | 0 |
| AM01760 |  |  | |  |  |  |  |  |  | 0 |
| AM01763 |  |  | |  |  |  |  |  |  | 0 |
| AM01799 |  |  | |  |  |  |  |  |  | 0 |
| AM01804 |  | Eip93F | |  |  |  |  |  |  | 0 |
| AM01818 |  |  | |  |  |  |  |  |  | 0 |
| AM01833 |  |  | |  |  |  |  |  |  | 0 |
| AM01838 |  |  | |  |  |  |  |  |  | 0 |
| AM01845 |  |  | |  |  |  |  |  |  | 0 |
| AM01867 |  |  | |  |  |  |  |  |  | 0 |
| AM01877 |  |  | |  |  |  |  |  |  | 0 |
| AM01880 |  |  | |  |  |  |  |  |  | 0 |
| AM01881 |  |  | |  |  |  |  |  |  | 0 |
| AM01889 |  |  | |  |  |  |  |  |  | 0 |
| AM01892 |  |  | |  |  |  |  |  |  | 0 |
| AM01901 |  |  | |  |  |  |  |  |  | 0 |
| AM01911 |  |  | |  |  |  |  |  |  | 0 |
| AM01921 |  |  | |  |  |  |  |  |  | 0 |
| AM01939 |  |  | |  |  |  |  |  |  | 0 |
| AM01973 |  |  | |  |  |  |  |  |  | 0 |
| AM01986 |  |  | |  |  |  |  |  |  | 0 |
| AM01994 |  |  | |  |  |  |  |  |  | 0 |
| AM01996 |  |  | |  |  |  |  |  |  | 0 |
| AM02006 |  |  | |  |  |  |  |  |  | 0 |
| AM02013 |  | SP1070 | |  |  |  |  |  |  | 0 |
| AM02061 |  |  | |  |  |  |  |  |  | 0 |
| AM02062 |  |  | |  |  |  |  |  |  | 0 |
| AM02070 |  |  | |  |  |  |  |  |  | 0 |
| AM02110 |  |  | |  |  |  |  |  |  | 0 |
| AM02118 |  |  | |  |  |  |  |  |  | 0 |
| AM02120 |  |  | |  |  |  |  |  |  | 0 |
| AM02125 |  |  | |  |  |  |  |  |  | 0 |
| AM02128 |  |  | |  |  |  |  |  |  | 0 |
| AM02135 |  |  | |  |  |  |  |  |  | 0 |
| AM02144 |  |  | |  |  |  |  |  |  | 0 |
| AM02150 |  |  | |  |  |  |  |  |  | 0 |
| AM02154 |  |  | |  |  |  |  |  |  | 0 |
| AM02158 |  |  | |  |  |  |  |  |  | 0 |
| AM02160 |  |  | |  |  |  |  |  |  | 0 |
| AM02161 |  |  | |  |  |  |  |  |  | 0 |
| AM02162 |  |  | |  |  |  |  |  |  | 0 |
| AM02189 |  |  | |  |  |  |  |  |  | 0 |
| AM02196 |  |  | |  |  |  |  |  |  | 0 |
| AM02201 |  |  | |  |  |  |  |  |  | 0 |
| AM02224 |  |  | |  |  |  |  |  |  | 0 |
| AM02239 |  |  | |  |  |  |  |  |  | 0 |
| AM02247 |  |  | |  |  |  |  |  |  | 0 |
| AM02259 |  |  | |  |  |  |  |  |  | 0 |
| AM02265 |  |  | |  |  |  |  |  |  | 0 |
| AM02281 |  |  | |  |  |  |  |  |  | 0 |
| AM02318 |  |  | |  |  |  |  |  |  | 0 |
| AM02324 |  |  | |  |  |  |  |  |  | 0 |
| AM02333 |  |  | |  |  |  |  |  |  | 0 |
| AM02366 |  |  | |  |  |  |  |  |  | 0 |
| AM02367 |  |  | |  |  |  |  |  |  | 0 |
| AM02377 |  |  | |  |  |  |  |  |  | 0 |
| AM02387 |  |  | |  |  |  |  |  |  | 0 |
| AM02396 |  |  | |  |  |  |  |  |  | 0 |
| AM02412 |  |  | |  |  |  |  |  |  | 0 |
| AM02419 |  |  | |  |  |  |  |  |  | 0 |
| AM02439 |  |  | |  |  |  |  |  |  | 0 |
| AM02442 |  |  | |  |  |  |  |  |  | 0 |
| AM02481 |  |  | |  |  |  |  |  |  | 0 |
| AM02488 |  |  | |  |  |  |  |  |  | 0 |
| AM02496 |  |  | |  |  |  |  |  |  | 0 |
| AM02522 |  |  | |  |  |  |  |  |  | 0 |
| AM02530 |  |  | |  |  |  |  |  |  | 0 |
| AM02537 |  |  | |  |  |  |  |  |  | 0 |
| AM02540 |  |  | |  |  |  |  |  |  | 0 |
| AM02542 |  |  | |  |  |  |  |  |  | 0 |
| AM02560 |  |  | |  |  |  |  |  |  | 0 |
| AM02567 |  | Eip93F | |  |  |  |  |  |  | 0 |
| AM02585 |  |  | |  |  |  |  |  |  | 0 |
| AM02588 |  |  | |  |  |  |  |  |  | 0 |
| AM02617 |  |  | |  |  |  |  |  |  | 0 |
| AM02625 |  |  | |  |  |  |  |  |  | 0 |
| AM02687 |  |  | |  |  |  |  |  |  | 0 |
| AM03347 |  |  | |  |  |  |  |  |  | 0 |
| AM03480 |  |  | |  |  |  |  |  |  | 0 |
| AM03972 |  |  | |  |  |  |  |  |  | 0 |
| AM04053 |  |  | |  |  |  |  |  |  | 0 |
| AM04060 |  |  | |  |  |  |  |  |  | 0 |
| AM04205 |  |  | |  |  |  |  |  |  | 0 |
| AM04409 |  |  | |  |  |  |  |  |  | 0 |
| AM04435 |  |  | |  |  |  |  |  |  | 0 |
| AM04456 |  |  | |  |  |  |  |  |  | 0 |
| AM04639 |  |  | |  |  |  |  |  |  | 0 |
| AM04828 |  |  | |  |  |  |  |  |  | 0 |
| AM04865 |  |  | |  |  |  |  |  |  | 0 |
| AM05215 |  |  | |  |  |  |  |  |  | 0 |
| AM05255 |  |  | |  |  |  |  |  |  | 0 |
| AM05638 |  |  | |  |  |  |  |  |  | 0 |
| AM05946 |  |  | |  |  |  |  |  |  | 0 |
| AM05960 |  |  | |  |  |  |  |  |  | 0 |
| AM06206 |  |  | |  |  |  |  |  |  | 0 |
| AM06589 |  |  | |  |  |  |  |  |  | 0 |
| AM06657 |  |  | |  |  |  |  |  |  | 0 |
| AM06712 |  |  | |  |  |  |  |  |  | 0 |
| AM07023 |  |  | |  |  |  |  |  |  | 0 |
| AM07063 |  |  | |  |  |  |  |  |  | 0 |
| AM07565 |  |  | |  |  |  |  |  |  | 0 |
| AM07675 |  |  | |  |  |  |  |  |  | 0 |
| AM07800 |  |  | |  |  |  |  |  |  | 0 |
| AM07823 |  |  | |  |  |  |  |  |  | 0 |
| AM08466 |  |  | |  |  |  |  |  |  | 0 |
| AM08914 |  |  | |  |  |  |  |  |  | 0 |
| AM09067 |  |  | |  |  |  |  |  |  | 0 |
| AM09212 |  |  | |  |  |  |  |  |  | 0 |
| AM09265 |  |  | |  |  |  |  |  |  | 0 |
| AM09506 |  |  | |  |  |  |  |  |  | 0 |
| AM09529 |  |  | |  |  |  |  |  |  | 0 |
| AM09580 |  |  | |  |  |  |  |  |  | 0 |
| AM09624 |  |  | |  |  |  |  |  |  | 0 |
| AM09699 |  |  | |  |  |  |  |  |  | 0 |
| AM09801 |  |  | |  |  |  |  |  |  | 0 |
| AM10092 |  |  | |  |  |  |  |  |  | 0 |
| AM10109 |  |  | |  |  |  |  |  |  | 0 |
| AM10160 |  |  | |  |  |  |  |  |  | 0 |
| AM10164 |  |  | |  |  |  |  |  |  | 0 |
| AM10179 |  |  | |  |  |  |  |  |  | 0 |
| AM10180 |  |  | |  |  |  |  |  |  | 0 |
| AM10186 |  |  | |  |  |  |  |  |  | 0 |
| AM10534 |  |  | |  |  |  |  |  |  | 0 |
| AM10638 |  |  | |  |  |  |  |  |  | 0 |
| AM10778 |  |  | |  |  |  |  |  |  | 0 |
| AM10888 |  |  | |  |  |  |  |  |  | 0 |
| AM10893 |  |  | |  |  |  |  |  |  | 0 |
| AM11125 |  |  | |  |  |  |  |  |  | 0 |
| AM11156 |  |  | |  |  |  |  |  |  | 0 |
| AM11202 |  |  | |  |  |  |  |  |  | 0 |
| AM11405 |  |  | |  |  |  |  |  |  | 0 |
| AM11517 |  |  | |  |  |  |  |  |  | 0 |
| AM11565 |  |  | |  |  |  |  |  |  | 0 |
| AM11881 |  |  | |  |  |  |  |  |  | 0 |
| AM11885 |  |  | |  |  |  |  |  |  | 0 |
| AM12020 |  |  | |  |  |  |  |  |  | 0 |
| AM12039 |  |  | |  |  |  |  |  |  | 0 |
| AM12095 |  |  | |  |  |  |  |  |  | 0 |
| AM12251 |  |  | |  |  |  |  |  |  | 0 |
| AM12297 |  |  | |  |  |  |  |  |  | 0 |
| AM12699 |  |  | |  |  |  |  |  |  | 0 |
| AM12723 |  |  | |  |  |  |  |  |  | 0 |
| AM12743 |  |  | |  |  |  |  |  |  | 0 |
| AM12765 |  |  | |  |  |  |  |  |  | 0 |
| AM12813 |  |  | |  |  |  |  |  |  | 0 |
| AM12840 |  |  | |  |  |  |  |  |  | 0 |
| AM12844 |  |  | |  |  |  |  |  |  | 0 |
| AM12844R |  |  | |  |  |  |  |  |  | 0 |
| AM12845 |  |  | |  |  |  |  |  |  | 0 |
| AM12845R |  |  | |  |  |  |  |  |  | 0 |
| AM12850R |  |  | |  |  |  |  |  |  | 0 |
| AM12852R |  |  | |  |  |  |  |  |  | 0 |
| AM12864 |  |  | |  |  |  |  |  |  | 0 |
| AM12864R |  |  | |  |  |  |  |  |  | 0 |
| AM12877R |  |  | |  |  |  |  |  |  | 0 |
| AM12899 |  |  | |  |  |  |  |  |  | 0 |

**S8. Comparative studies gene lists.** Significantly-regulated transcripts in this study were compared to previously published expression studies in honey bees. The list of overlapping transcripts are included in this file.
