# Supplementary figures and images for: Individual Variation in Pheromone Response Correlates with Reproductive Traits and Brain Gene Expression in Worker Honey Bees
Source: PLoS One. 2010 Feb 9;5(2):e9116. doi: 10.1371/journal.pone.0009116 (PMC2817734; doi:10.1371/journal.pone.0009116)

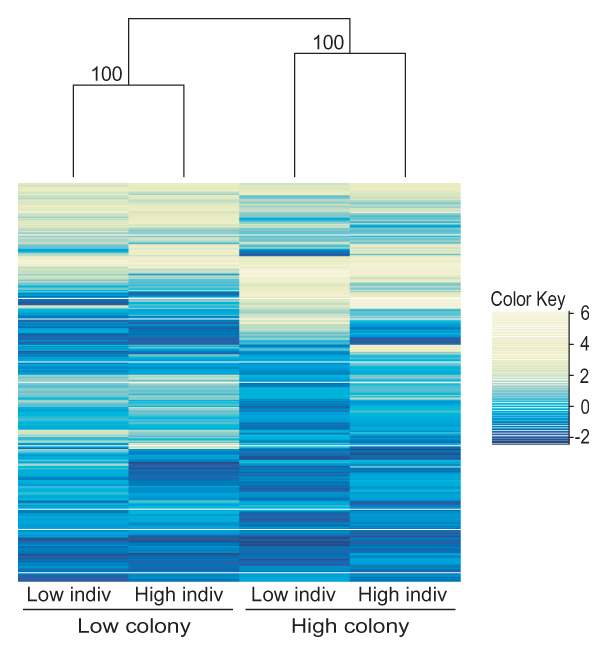

Supplement: Figure S1 — Hierarchical clustering. Hierarchical clustering of the 960 significant retinue response genes (FDR<0.01) reveals that behavioral groups cluster based primarily on colony-level differences and not on individual retinue response behavior. (1.19 MB TIF) [file pone.0009116.s003.tif]
